# A KLK6 Activity-Based Probe Reveals a Role for KLK6 Activity in Pancreatic Cancer Cell Invasion

**DOI:** 10.1021/jacs.2c07378

**Published:** 2022-11-22

**Authors:** Leran Zhang, Scott Lovell, Elena De Vita, Pravin Kumar Ankush Jagtap, Daniel Lucy, Andrea Goya Grocin, Svend Kjær, Annabel Borg, Janosch Hennig, Aubry K. Miller, Edward W. Tate

**Affiliations:** † Department of Chemistry, Molecular Sciences Research Hub, Imperial College London, London W12 0BZ, U.K.; ‡ Department of Life Sciences, University of Bath, Bath BA2 7AX, U.K.; § Structural and Computational Biology Unit, European Molecular Biology Laboratory, Heidelberg 69117, Germany; ∥ Chair of Biochemistry IV, Biophysical Chemistry, University of Bayreuth, Bayreuth 95447, Germany; ⊥ Structural Biology Science Technology Platform, The Francis Crick Institute, London NW1 1AT, U.K.; # Cancer Drug Development Group, German Cancer Research Center (DKFZ), Heidelberg 69120, Germany

## Abstract

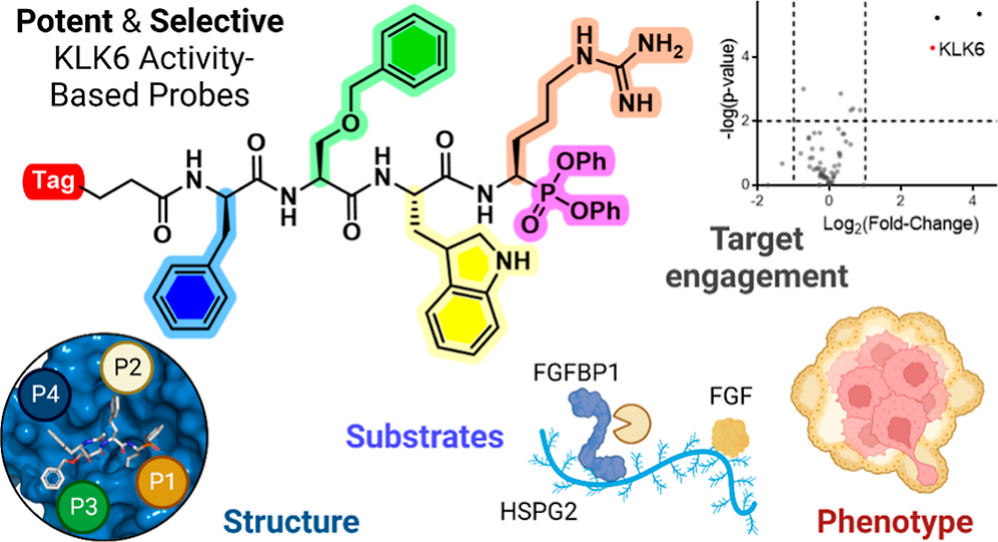

Pancreatic cancer has the lowest survival rate of all common cancers due to late diagnosis and limited treatment options. Serine hydrolases are known to mediate cancer progression and metastasis through initiation of signaling cascades and cleavage of extracellular matrix proteins, and the kallikrein-related peptidase (KLK) family of secreted serine proteases have emerging roles in pancreatic ductal adenocarcinoma (PDAC). However, the lack of reliable activity-based probes (ABPs) to profile KLK activity has hindered progress in validation of these enzymes as potential targets or biomarkers. Here, we developed potent and selective ABPs for KLK6 by using a positional scanning combinatorial substrate library and characterized their binding mode and interactions by X-ray crystallography. The optimized KLK6 probe IMP-2352 (*k*_obs_/*I* = 11,000 M^–1^ s^–1^) enabled selective detection of KLK6 activity in a variety of PDAC cell lines, and we observed that KLK6 inhibition reduced the invasiveness of PDAC cells that secrete active KLK6. KLK6 inhibitors were combined with N-terminomics to identify potential secreted protein substrates of KLK6 in PDAC cells, providing insights into KLK6-mediated invasion pathways. These novel KLK6 ABPs offer a toolset to validate KLK6 and associated signaling partners as targets or biomarkers across a range of diseases.

## Introduction

Kallikrein-related peptidases (KLKs) compose a family of 15 secreted serine proteases which orchestrate a protease cross-activation network known as the KLK activome, with a variety of roles in homeostasis and disease.^1^ Dysregulation of this network is linked with neurodegenerative diseases, skin diseases and cancer,^2^ and active KLKs are recognized as potential biomarkers and drug targets in these diseases. Elevated levels of specific KLKs correlate with poor prognosis in many cancer types,^2^ notably KLK3 (or prostate-specific antigen, PSA) which is a biomarker for prostate cancer diagnosis and treatment monitoring, with recent reports also revealing important roles for KLK2 and KLK14.^3−5^

The five-year survival rate of pancreatic cancer is 10%, the lowest rate among all common cancers,^6^ and there is an urgent need for early detection and reliable treatments for this genetically heterogeneous disease. Elevated KLK mRNA levels correlate with worse outcome in pancreatic ductal adenocarcinoma (PDAC),^7^ promoting metastasis by degrading the extracellular matrix.^8−11^ KLK6 and KLK10^12^ co-expression is a marker of poor prognosis,^7,13^ with KLK6 protein highly expressed in invasive PDAC tissue compared to normal tissue.^7^ However, a current lack of approaches to specifically detect active KLKs has limited previous studies to measuring total mRNA or protein levels rather than functional KLK activity.^7,13−16^ KLKs are secreted from the cell as inactive pro-KLKs and must be subsequently cleaved to generate the active form, while endogenous protease inhibitors can further modulate KLK activity.^17^ Probes to detect and quantify specific KLK activities would enable exploration of specific KLK proteolytic activities as a biomarker for cancer prognosis or progression and validation of KLKs as potential drug targets, as well as assist identification of selective KLK inhibitors, which is particularly challenging due to the high homology between family members and substrate primary sequence specificities which can overlap with other proteases.^18^

**Figure 1:**
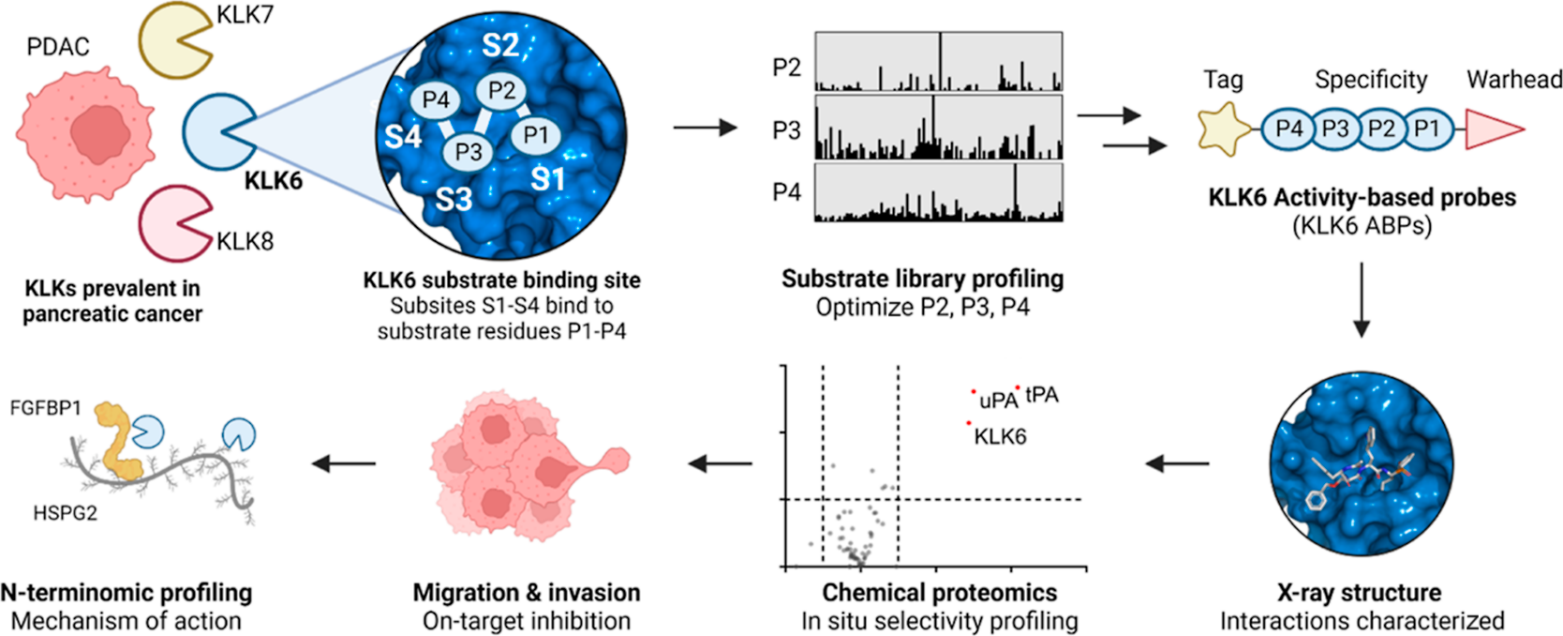
KLK6 ABP development and applications. Designs for ABPs selective for KLK6 over other KLKs abundant in PDAC (KLK7 and KLK8) were based on the preferred natural and unnatural amino acids associated with each KLK6 subsite (S1–S4) discovered using a combinatorial positional scanning library. On-target activity of an optimized biotinylated ABP (bABP **17**, IMP-2352) was analyzed by X-ray crystallography and chemical proteomics; the probe was shown to inhibit PDAC cell invasion and migration and used to identify potential KLK6 substrates mediating this phenotype in PDAC cells in combination with N-terminal proteome analysis (N-terminomics).

Here, we report discovery and development of the first selective and potent activity-based probes (ABPs) and inhibitors for KLK6 and their application in cellular models of PDAC.^7,13,19^ These KLK ABPs consist of three components (Figure 1). First, an electrophilic diphenylphosphonate warhead that binds irreversibly to the active site serine residue on the KLK, and which bears a side chain designed to extend into the protease S1 pocket. Second, a specificity region based on a tripeptide with amino acids targeting the S2 to S4 binding pockets; this sequence is derived from a positional scanning substrate combinatorial library featuring a wide range of unnatural amino acids which can impart selectivity and potency by exploiting interactions unavailable to endogenous natural substrates.^20^ Finally, a fluorophore or biotin tag is attached via a flexible linker, allowing analysis by fluorescence or streptavidin binding, respectively. Activity-based protein profiling (ABPP) using these probes would permit quantification of active KLK6 directly from a complex biological sample, a methodology previously applied to generate ABPs selective for proteases such as cathepsins^21,22^ or elastases,^23,24^ and in our recent disclosure of selective ABPs for KLK2, KLK3, and KLK14 in the context of prostate cancer.^4^ To the best of our knowledge, these are the first selective KLK6 probes stringently profiled for selectivity de novo in a complex biological system through liquid chromatography–tandem mass spectrometry (LC–MS/MS) analysis, which provides a further layer of validation over previously claimed KLK probes.^25−27^

## Results

### KLK6 is Overexpressed and Active in Capan-2 Pancreatic Cancer Cells

We first determined active serine hydrolases (the SH activome) in the supernatant of Capan-2 cells, which show high KLK expression based on Human Protein Atlas mRNA-seq data.^28^ Concentrated serum-free RPMI media (conditioned media) prepared from Capan-2 cells was analyzed by label-free quantitative chemical proteomics using the broad-spectrum SH ABP FP-biotin (10 μM, 1 h), compared against vehicle (DMSO) control. KLK6 was found to be active in this cell line, consistent with mRNA expression data (Figure S1)^13^ providing a model for selective KLK6 ABP development. Since KLK8 is typically co-expressed with KLK6 in multiple disease contexts, including in pancreatic cancer,^16,29−31^ initial probe development focused on an ABP selective toward KLK6 over KLK8, a challenging objective in view of their common trypsin-like specificity for Lys/Arg at P1.

### Profiling KLK6 Substrate Specificity Using a Combinatorial Positional Scanning Substrate Library

**Figure 2:**
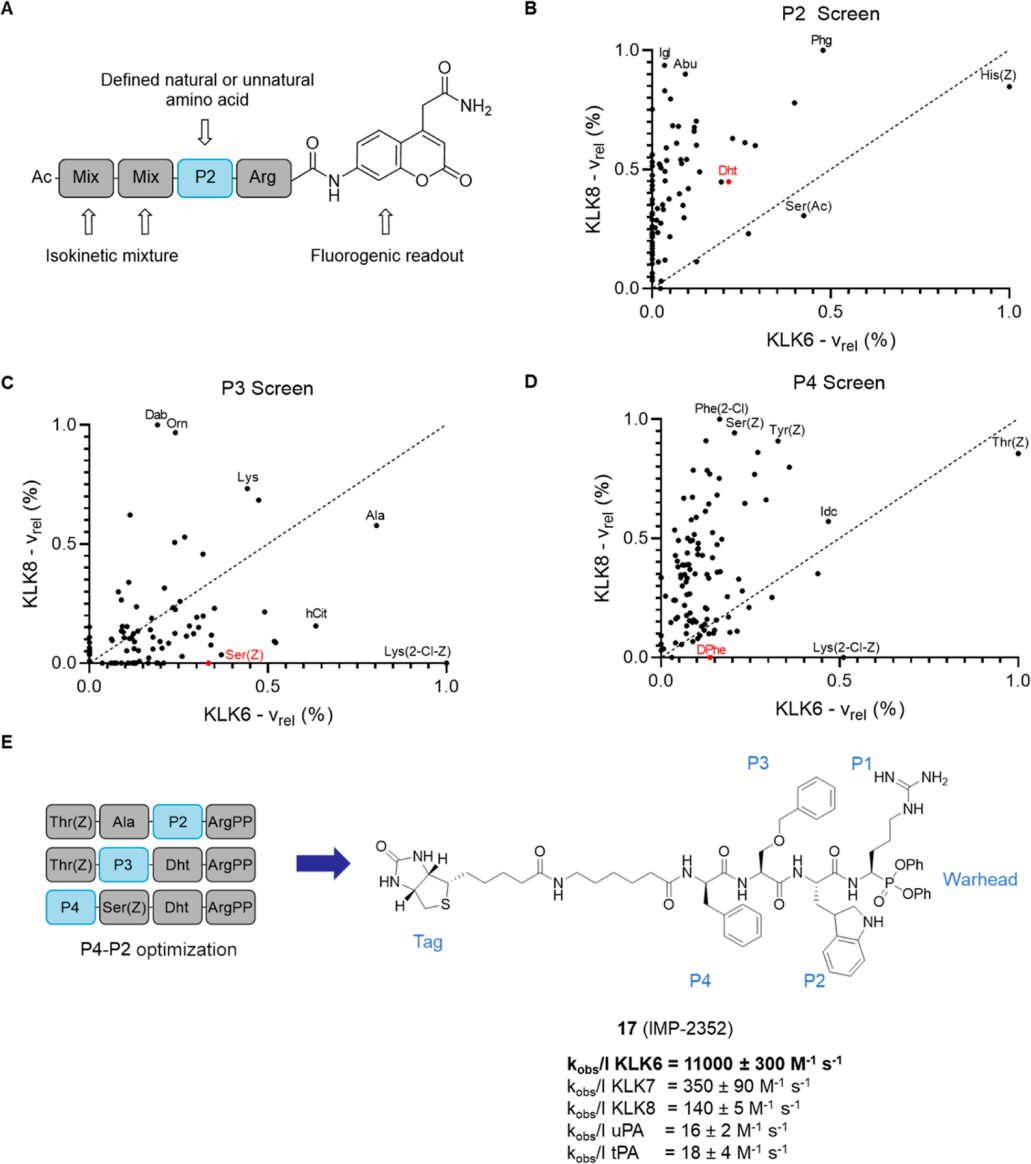
Design of selective KLK ABPs using a fluorogenic peptide library. (A) Structure of peptides in the library, exemplified by the P2 sub-library. The isokinetic mixture results in an equimolar ratio of all natural amino acids at the randomized positions but with norleucine replacing methionine and cysteine. (B–D) Scatter plots comparing the substrate specificities of KLK6 and KLK8 in the P2, P3, and P4 positions. The axes represent the cleavage velocity (*v*_rel_) of a peptide in the library, relative to the highest velocity (set to 1) for KLK6 (*x*-axis) and KLK8 (*y*-axis). Amino acids above the dashed line are preferred by KLK8, while amino acids below the dashed line are preferred by KLK6. The top three amino acids for each KLK are labeled in black, while the final optimized amino acid for the KLK6 ABP is labeled in red. (E) P2–P4 probe optimization strategy. All probes were tested for potency (*k*_obs_/*I*) using pseudo-first-order kinetics. Structure and selectivity of optimized biotinylated KLK6 ABP **17** (IMP-2352).

We first explored substrate scope and specificity of recombinant purified KLK6 (rhKLK6) using a fluorogenic peptide substrate library.^4,20^ Three sub-libraries (named P2, P3, and P4) were designed to investigate amino acids likely to be recognized in the S2, S3, and S4 binding pockets, respectively, with all peptides featuring N-terminal acetylation and a C-terminal P1 arginine (Figure 2A).^32^ Each library contained 106 individual combinatorial sub-libraries of 361 fluorogenic peptides, each with a defined natural or unnatural amino acid at the specified position and a combinatorial mixture of natural amino acids at the remaining positions, covering 114,798 sequences in total (Figure S2). For example, the P2 library has the general sequence Ac–Xaa–Xaa–P2–Arg–ACC, where “Ac”is the acetylated N-terminus, Xaa is introduced as an equimolar mixture of all natural amino acids with norleucine in place of methionine and cysteine, “P2” is one of 106 defined natural or unnatural amino acids, “Arg” is the P1 arginine, and “ACC” is an aminocarbamoyl methylcoumarin fluorophore. The rate of cleavage for each fluorogenic substrate library can be used to infer amino acid tolerance at each specified position (P2, P3, or P4). A screen of all 318 combinatorial substrates against KLK6 revealed a preference for aromatic amino acids at P2 [e.g., l-His(Z) and Phg], l-Ala and l-Lys(2-Cl-Z) at P3, and l-Thr(Z) and l-Lys(2-Cl-Z) at P4 (Figure 2B–D; see Figure S2 for full substrate specificity profiles, Figure S3 for amino acid identities).

### Highly Selective KLK6 ABP

A first-generation ABP was designed using the top amino acid hits from the positional scanning library. This template sequence was systematically optimized over three iterations of synthesis and testing guided by substrate specificity data for both KLK6 and KLK8 (Figure 2E). 21 KLK6 ABPs with varied combinations of the most selective amino acids were synthesized in total, creating a targeted structure–activity relationship matrix (Table 1, Figures S4 and S5). The first two rounds of optimization used a P1 racemic arginine–mimetic diphenylphosphonate warhead [Phg(4-guan)PP], thanks to its ready synthetic accessibility, and in round one, probes (**1** to **8**) were initially capped with 4-pentynoic acid, giving an N-terminal alkyne (Yn) tag. The most potent ABP from round one (**5**) contained P2 Dht, which was carried into designs for round two (**9** to **15**), which carried an N-terminal biotin–Ahx (6-aminohexanoic acid) tag, allowing them to be used directly in cell-based experiments. ABP **11** was chosen as the optimal sequence from this round [DPhe–Cha–Dht–Phg(4-guan)], based on selectivity over KLK8 (Table 1). In the final round of optimization, the P1 position was switched to a racemic arginine-based warhead design, ArgPP (**16** to **21**), giving a six-fold increase in potency (**11** vs **16**). An optimal balance of potency and selectivity was found for DPhe–Ser(Z)–Dht–ArgPP (P4 to P1), with potency driven primarily by P2 l-Dht and P3 l-Ser(Z), and P4 d-Phe providing selectivity, as the only d-amino acid tolerated by KLK6 but not by KLK8. Biotinylated (bABP **17**), alkyne-tagged (YnABP **19**), and Cy5-tagged (fABP **21**) ABPs were synthesized and validated in biochemical assays. bABP **17** is highly potent for KLK6 (*k*_obs_/*I* = 11,000 M^–1^ s^–1^), selective over KLK7 (>30-fold) and KLK8 (>70-fold), and >600-fold selective over two serine proteases particularly highly expressed in PDAC: urokinase plasminogen activator (uPA) and tissue plasminogen activator (tPA)^33^ (Figure 2E). ABPs were synthesized as a 50/50 mixture of diastereoisomers epimeric at the warhead, which were separated by HPLC to yield the paired active ABP and control inactive isomer (**17**/**18** and **19**/**20**). The absolute stereochemistry of the active ABP was posited to mimic a natural P1 l-amino acid, and this was later confirmed for **16** and **17** by X-ray crystallography (see below, Figures 4 and S10).

**Table 1:** *k*_obs_/*I* Values for KLK6 ABP Analogues^a^

code	compound sequence	KLK6 *k*_obs_/*I* (M^–1^ s^–1^)	95% CI	KLK8 *k*_obs_/*I* (M^–1^ s^–1^)	95% CI
round 1					
1	Yn–Thr(Z)–Ala–Phg–Phg(4-guan)PP	220	210–230		
2	Yn–Thr(Z)–Chg–His(Z)–Phg(4-guan)PP	30	24–36		
3	Yn–Thr(Z)–Cha–His(Z)–Phg(4-guan)PP	46	40–52		
4	Yn–Asp(Chx)–Cha–His(Z)–Phg(4-guan)PP	54	51–57		
5	Yn–Thr(Z)–Ala–Dht–Phg(4-guan)PP	250	240–260		
6	Yn–Thr(Z)–Ala–Tyr(Z)–Phg(4-guan)PP	7	5–9		
7	Yn–Thr(Z)–Ala–Ser(Z)–Phg(4-guan)PP	43	33–53		
8	Yn–Thr(Z)–Ala–Dab(Z)–Phg(4-guan)PP	11	7–15		
round 2					
9	biotin–Ahx–Thr(Z)–Ala–Dht–Phg(4-guan)PP	220	210–230		
10	biotin–Ahx–Thr(Z)–Cha–Dht–Phg(4-guan)PP	420	400–440	71	68–84
11	biotin–Ahx–DPhe–Cha–Dht–Phg(4-guanPP	210	200–220	0	0
12	biotin–Ahx–DhPhe–Cha–Dht–Phg(4-guan)PP	140	110–170	0	0
13	biotin–Ahx–DPhe–Lys–Dht–Phg(4-guan)PP	250	230–270		
14	biotin–Ahx–DPhe–DLys–Dht–Phg(4-guan)PP	550	520–580	73	68–78
15	biotin–Ahx–DPhe–Cha–Ser(Ac)–Phg(4-guan)PP	150	140–160	20	14–26
round 3					
16	biotin–Ahx–DPhe–Cha–Dht–ArgPP	1300	1230–1370	150	141–159
17	biotin–Ahx–DPhe–Ser(Z)–Dht–ArgPP	11000	10700–11300	140	135–145
18	biotin–Ahx–DPhe–Ser(Z)–Dht–DArgPP	0	0		
19	Yn–DPhe–Ser(Z)–Dht–ArgPP	3000	2880–3120		
20	Yn–DPhe–Ser(Z)–Dht–DArgPP	0	0		
21	Cy5–DPhe–Ser(Z)–Dht–ArgPP	1000	900–1100	40	34–46

a The chemical structures of each amino acid abbreviation are listed in Figure S3. Definitions of each tag are as follows: Yn = 4-pentynoic acid, biotin = d-biotin, and Cy5 = cyanine-5 with a triazole linker. Phg(4-guan)PP = diphenyl (amino(4-guanidinophenyl)methyl)phosphonate and ArgPP = diphenyl (1-amino-4-guanidinobutyl)phosphonate.

### KLK6 ABPs Selectively Engage Endogenous Active KLK6

**Figure 3:**
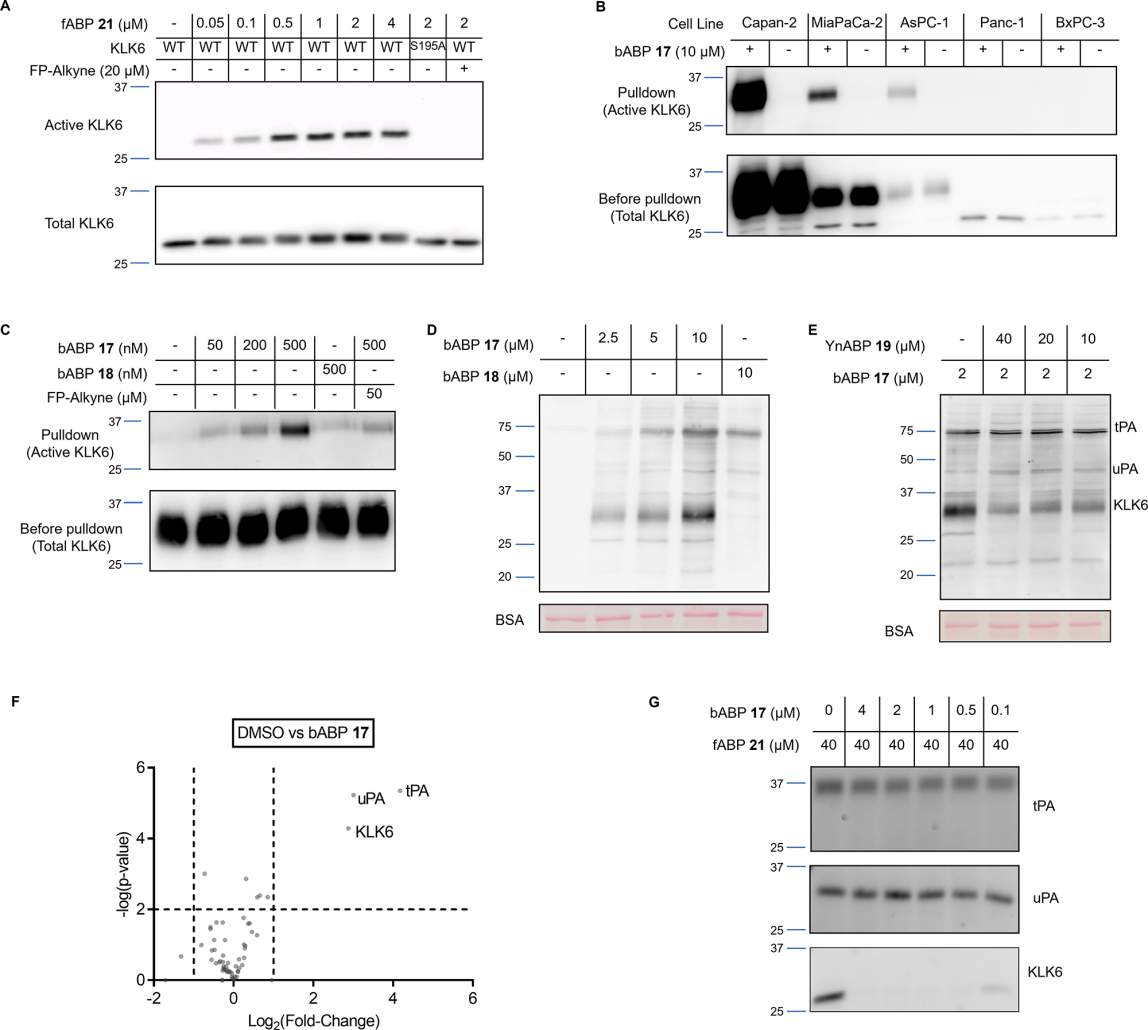
ABP validation. (A) In-gel fluorescence showing active rhKLK6 labeled by fABP **21** after 1 h incubation. A KLK6 western blot of the gel shows total KLK6. WT = wild-type, S195A = mutated inactive KLK6. (B) KLK6 blot of a pulldown experiment with bABP **17** (IMP-2352) on conditioned media from a panel of PDAC cell lines. Pulldown lanes show enriched proteins after streptavidin binding. Before pulldown shows total KLK6 in each cell line. (C) KLK6 blot of a pulldown experiment with bABP **17** (IMP-2352) at different concentrations on Capan-2 conditioned media. The inactive control **18** shows no labeling. 2 h preincubation with FP-alkyne reduces the labeling of bABP **17** (IMP-2352). (D) NeutrAvidin-HRP blot of Capan-2 media after incubation of compounds **17** (IMP-2352) and **18** for 1 h. (E) NeutrAvidin-HRP blot of Capan-2 media after incubation of the KLK6-selective alkyne probe (YnABP) **19** for 2 h, followed by bABP **17** (IMP-2352) for 1 h. This competition experiment shows that YnABP **19** selectively inhibits KLK6 over tPA and uPA. (F) KLK6 ABP selectivity and on-target validation using LC–MS/MS. Capan-2 conditioned media was treated with bABP **17** (IMP-2352) or DMSO for 1 h, followed by a chemical proteomics workflow to enrich for labeled proteins. The volcano plot compares the changes of protein quantification between DMSO and ABP-treated media, showing the targets of bABP **17** (IMP-2352). (G) Recombinant tPA, uPA, or KLK6 were treated with bABP **17** (IMP-2352) for 2 h, followed by fABP **21** for 1 h. In-gel fluorescence shows selective inhibition of KLK6 over tPA and uPA by bABP **17**.

Activity-based target engagement of the optimized ABPs was first tested for fABP **21** on recombinant wild-type KLK6 or inactive KLK6[S195A] mutated at the active site serine residue. In-gel fluorescence showed dose-dependent labeling of active KLK6, while the mutant was not labeled (Figure 3A), and labeling was blocked by preincubation with the broad-spectrum serine hydrolase inhibitor, fluorophosphonate-alkyne (FP-alkyne). We next quantified KLK6 activity in the supernatant of a panel of pancreatic cancer cells using bABP **17** (IMP-2352) (Figure 3B). Conditioned media was incubated with bABP **17** (10 μM) or DMSO for 2 h, and labeled proteins enriched on magnetic streptavidin-loaded beads, eluted, and analyzed by western blot against a KLK6 antibody. KLK6 was detected in all five PDAC cell lines before pulldown (Figure 3B); however, active KLK6 was only enriched from Capan-2, MIA PaCa-2, and AsPC-1 conditioned media. The KLK6 antibody detected two distinct bands at 31 and 25 kDa, but only the 31 kDa band appears to represent active KLK6 based on pulldown analysis. PNGase F treatment shifted the 31 kDa band to 28 kDa, showing that the 31 kDa band is the glycosylated form of KLK6 (Figure S6);^34^ this deglycosylated form retained activity (Figure S7), leading us to hypothesize that the residual 25 kDa band is a cleaved and inactivated form of KLK6.^32^ In Capan-2 conditioned media, bABP **17** (IMP-2352) detected active KLK6 in a dose-dependent manner from as little as 50 nM probe (Figure 3C), and probe labeling was reduced by FP-alkyne preincubation, while the inactive epimeric probe **18** showed no KLK6 labeling. Together, these data demonstrate that bABP **17** (IMP-2352) efficiently labels endogenous active KLK6.

Next, we applied chemical proteomics to explore target engagement and probe selectivity de novo in Capan-2 conditioned media; chemical proteomics provides a unique insight into potential on- and off-targets of an inhibitor or probe in a complex biological system.^35^ Suitable conditions for chemical proteomic LC–MS/MS analysis of probe targets were established in Capan-2 conditioned media incubated with bABP **17** (IMP-2352) or inactive control **18**, with labeled proteins visualized by blotting against NeutrAvidin-HRP. bABP **17** (IMP-2352) strongly labeled a main band at 31 kDa, the molecular weight of KLK6 (Figure 3D), and this band was efficiently outcompeted by YnABP **19**, consistent with engagement of active KLK6 (Figure 3E). While **18** did not label this band, it weakly labeled a few additional bands to the same extent as **17** (IMP-2352), providing an indication of non-specifically labeled proteins. Only three proteases were significantly enriched by **17** (IMP-2352; 2 μM, 1 h) over DMSO control: KLK6, along with two off-targets, uPA and tPA (Figure 3F). Consistent with western blotting (Figure 3D), **18** showed labeling of tPA and tPA but not KLK6 under LC–MS/MS analysis (Figure S8). We previously established that **17** (IMP-2352) is poorly active against uPA and tPA (Figure 2E), suggesting that these off-targets are enriched due to their high abundance in this system. Based on probe competition experiments and assignment of labeled bands at the predicted molecular weights of tPA and uPA, it appears that YnABP **19** also does not substantively engage endogenous tPA and uPA at concentrations as high as 40 μM (Figure 3E). We confirmed the selectivity of bABP **17** (IMP-2352) by competitive ABPP, through incubation with recombinant tPA, uPA, or KLK6 followed by addition of fABP **21** to label residual protease activity. **17** (IMP-2352) was shown to selectively inhibit KLK6 over the two off-targets (Figure 3G). Taken together, these data suggest that bABP **17** (IMP-2352), YnABP **19**, and fABP **21** are highly selective for KLK6 and unlikely to functionally inhibit other proteases, including uPA and tPA; these compounds thus offer the first selective KLK6 inhibitors and ABPs suitable for phenotypic studies.

### KLK6 ABPs Interact with Specific KLK6 Subsites through Unnatural Amino Acid Side Chains

**Figure 4:**
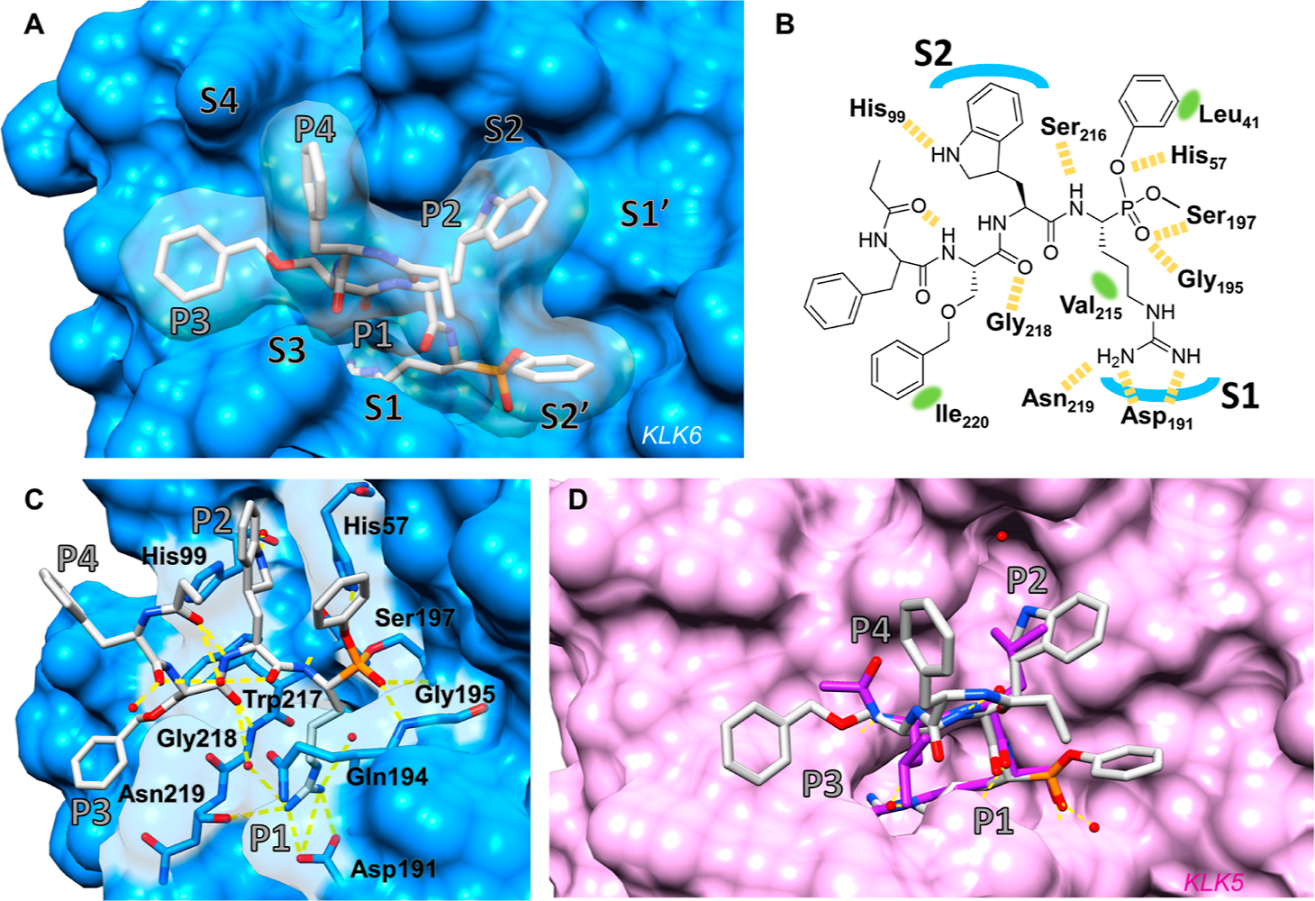
Structure-based studies. (A) Co-crystal structure of bABP **17** IMP-2352 (gray) with KLK6 (blue) determined to 1.6 Å [Protein data bank (PDB): 7QFV, chain B] illustrating the binding mode of subsites P1–P4 of bABP **17** inside the S1–S4 binding pockets of KLK6. (B) 2D illustration of IMP-2352 and its interactions with KLK6. Yellow dashed line = hydrogen bond, green area = proximity to lipophilic area. (C) Polar interactions including water network engaged by IMP-2352 in the active site of KLK6 following covalent binding of Ser197. (D) Superimposition of IMP-2352 (PDB: 7QFV) with co-crystal structure of leupeptin (purple) with KLK5 (pink) (PDB:2PSX). Oxygens, red; nitrogen, blue; phosphorus, orange; and hydrogen bonds, yellow.

We next investigated the binding mode of KLK6 ABPs by X-ray crystallography, to illuminate the unnatural amino acid interactions underpinning biochemical potency and specificity. Structures were solved for recombinant KLK6 covalently modified with bABP **16** or **17** (PDB: 7QFT, 7QFV) to 1.5 and 1.6 Å resolution, respectively, revealing extensive interactions across the S1–S4 pockets of KLK6 (Figures 4A and S10). Both structures identified density at covalent bond length to the active site serine residue (Ser197) resulting in an (*R*) configuration at the new phosphorous stereocenter, while the P1 arginine side chain of the probe forms multiple hydrogen bonds (H-bonds) with the S1 pocket of KLK6, confirming the proposed stereochemistry at the alpha stereocenter (Figure 4B,C). Polar interactions observed between His57/Gly195 and oxygens of the phosphonate are consistent with transition-state mimicry typical for an aryl phosphonate warhead.^36^ The position of the warhead phenyl group is less defined across the various model chains, as expected since this moiety has not been optimized for binding but points toward a hydrophobic region of the KLK6 surface created by Leu41. This hints that further optimization on this side of the molecule could provide extended binding toward the S1′/S2′ pockets, which are ligandable with small molecules.^25,37^ Interactions with the main chain of the ABP are seen with Ser216 and Gly218,^36^ whereas the linker and label moieties are unresolved in these structures in line with the intent that they should minimally impact target binding by design. The P2 L-Dht side chain is well-poised in the S2 pocket, with the indoline nitrogen facing a water molecule buried in this pocket that mediates a network of H-bonds to His99 and to Tyr94/Ala96, providing an explanation for the increase in potency imparted by this amino acid (Figure 4C). The indoline stereocenter of l-Dht was introduced as an epimeric mixture, and the electron density at this amino acid is less well-defined compared to other parts of the molecule (Figure S9), suggesting that both enantiomers of l-Dht may interact with the S2 pocket. The S3 pocket is relatively shallow in KLK6 compared, for example, to the structurally closely related KLK5 (Figure 4D) and mostly occupied by polar backbone interactions such as the contact with Gly218 and with a buried water molecule. The P3 l-Ser(Z) side chain contacts an extended hydrophobic patch extending out of the S3 pocket (Ile220), offering an explanation for the increase in potency observed on switching from P3 l-Cha to P3 l-Ser(Z) (Table 1), since bABP **16** shows a less optimal fit in this area of the protein with a less defined binding mode for P3 that varies between the two KLK6 chains in the crystal structure (Figure S10). Interestingly, P4 d-Phe appears to be solvent exposed, leaving the S4 pocket mostly unoccupied; given the important role of this residue in imparting selectivity, we hypothesize that this side chain deters engagement with other proteases that do not tolerate a P4 d-amino acid. A strong intramolecular H-bond between the carbonyl of the last resolved amide in the ABP chain (through which the linker is attached) and the amide NH of P3, facilitated by intramolecular water bridges formed between carbonyl groups in the backbone (Figure 4C), suggests a role in maintaining the binding conformation and potential for future optimization through backbone cyclization. This constraining effect was also observed for bABP **16** (Figure S10). These structures present the first binding modes extending into the S3/S4 region for KLK6, and a comparison with the previously reported structure of the non-selective covalent inhibitor leupeptin bound to KLK5 (PDB: 2PSX) supports the hypothesis that these pockets are important for KLK6 selectivity (Figure 4D). While the anion formed from the leupeptin aldehyde superimposes on the bABP **17** phosphonate oxygen and S1/S2 pocket disposition is broadly conserved between the enzymes, the S3 and S4 pockets of KLK5 are wider and narrower, respectively, than those seen in KLK6, resulting in divergent trajectories for the P3 and P4 residues. The prominent KLK6 Ile220 is a Tyr residue in KLK5 and is directed out of the active site, resulting in a shallower surface with reduced hydrophobicity. Although for any covalent ligand the binding pose of the molecule prior to reaction with the active site serine may differ from the final resting position in the crystal structure, these structures provide a plausible explanation for the exquisite potency and selectivity for KLK6, which can be achieved in this ABP series.

### KLK6 Inhibition Reduces Invasion and Migration of PDAC Cells

**Figure 5:**
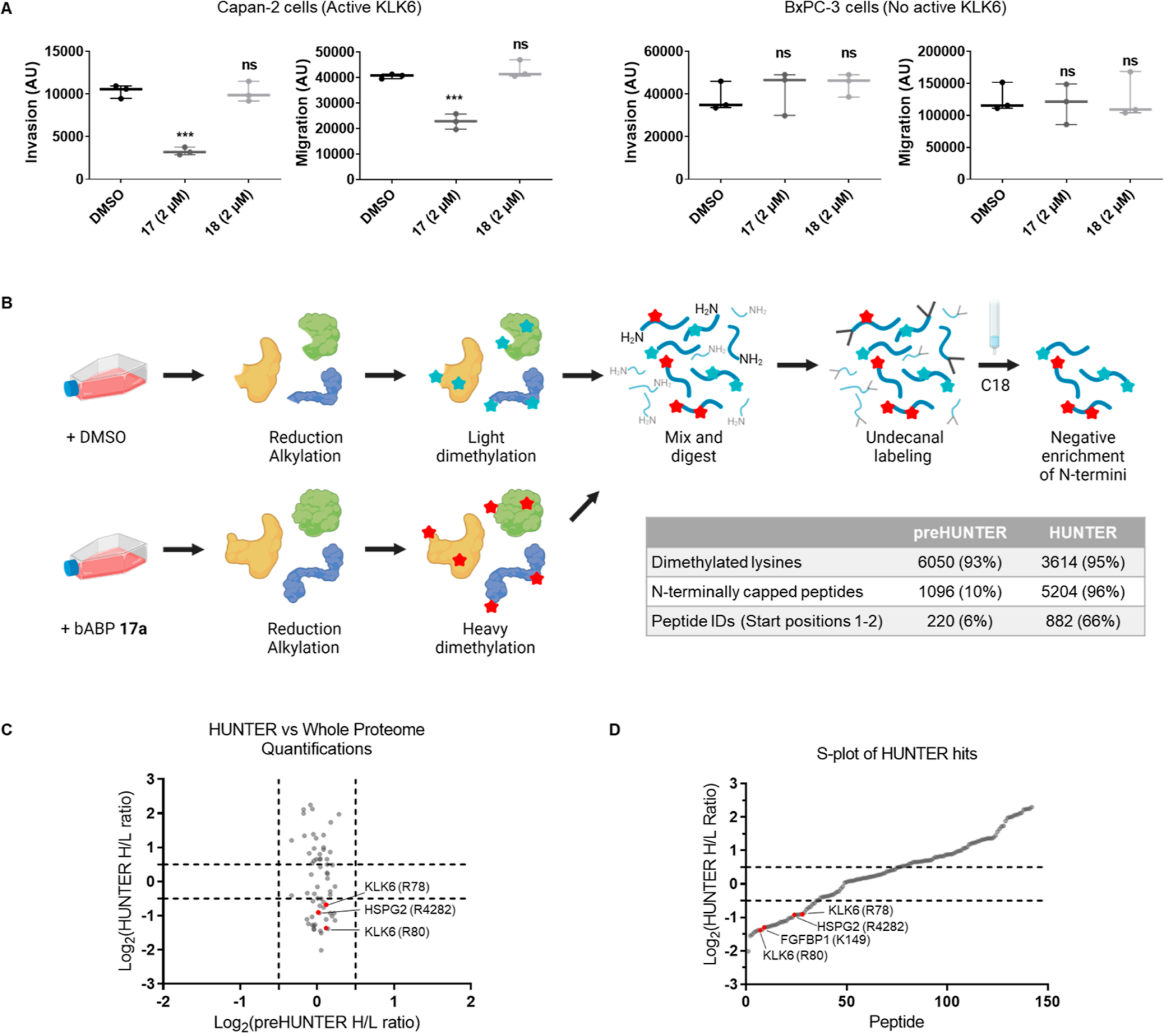
Applications of bABP **17** in cells. (A) Boyden chamber assays measuring invasion and migration of PDAC cells. Arbitrary units (AU) represent the area of cells in the image, quantified using ImageJ. Statistical significance was calculated against the DMSO control using a one-way ANOVA and Tukey’s test. *** = significant, ns = not significant. (B) HUNTER workflow for enriching N-terminal peptides from conditioned media. Capan-2 cells were grown in the presence of bABP **17** (IMP-2352) or DMSO for 24 h. Cell media was collected, concentrated, and used for HUNTER enrichment. The proteome was reduced and alkylated with TCEP and CAA for 45 min and dimethylated with heavy or light formaldehyde for 4 h. The proteome was mixed and digested using trypsin for 16 h. Newly generated free amines were labeled with undecanal and negatively enriched using a C18 column. The flow through was dried in vacuo, stage tipped, and submitted for LC–MS/MS analysis. The table shows pre-HUNTER data prior to undecanal labeling compared with the final HUNTER processed data. (C) Comparison of heavy/light ratios between the HUNTER-enriched and pre-HUNTER samples. (D) S-plot showing the Log_2_(*H*/*L* ratios) of the HUNTER significantly enriched peptides. Peptides are ordered on the *x*-axis based on their ratios. Hit substrates of interest are labeled.

It has been previously reported that KLK6 is involved in cancer cell invasion and migration,^38−41^ and so with multiple complementary lines of evidence supporting identification of the first selective and potent KLK6 ABPs, we proceeded to determine the impact of KLK6 activity and inhibition on proliferation, migration, and invasion of PDAC cells. bABP **17** (IMP-2352) and bABP **18** showed no effect on proliferation or cell viability when tested from 0.13 to 2 μM in Capan-2 cells by live cell imaging of cell death and proliferation (Figure S11), consistent with the role of KLKs as extracellular proteases and a recent report of non-toxicity of a protein-based KLK6 inhibitor.^40^ Next, the effect of KLK6 inhibition on migration and invasion of Capan-2 cells was assessed using a Boyden chamber assay. Capan-2 cells (which express active KLK6) or BxPC-3 cells (no active KLK6) were grown in the presence of vehicle (DMSO), bABP **17** (2 μM), or inactive isomer **18** (2 μM) for 24 h, and cells were stained with CellTracker CMHC (4-chloromethyl-7-hydroxycoumarin) to image nuclei by fluorescence microscopy. The cell area in each image was integrated to calculate parameters for migration and invasion (Figure 5A), with each data point calculated from an average of four images obtained from two separate Boyden chamber inserts. bABP **17** (IMP-2352) reduced both invasion and migration of Capan-2 cells but had no effect on the KLK6 negative cell line BxPC-3. **18** showed no effect in either line, relative to DMSO control, confirming the requirement for inhibition. Furthermore, bABP **17** (IMP-2352) did not label any intracellular protease when treated in live cells or in cell lysate (Figure S12); this is in line with the lack of intracellular activity of autoinhibited pro-KLK6, and the likely poor cellular uptake of the probe, and consistent with KLK6 inhibition restricted to the extracellular environment. Taken together, these data suggest a role for KLK6 in both migration and invasion of PDAC cells which depends on KLK6 activity.

### KLK6 Probes Enable Identification of Putative Endogenous KLK6 Substrates through N-Terminomics

By combining selective KLK6 inhibition with N-terminomic analysis, we sought to identify endogenous KLK6 substrates which may be involved in migration or invasion. N-terminomics focuses proteomic analysis on termini generated de novo on protease cleavage through enrichment and quantification of N-terminal tryptic peptides,^42^ and here, we applied a recently developed method termed high-efficiency undecanal-based N-termini enrichment (HUNTER)^43^ (Figure 5B). Conditioned media from Capan-2 cells grown in the presence of DMSO or bABP **17** (IMP-2352) was treated with TCEP and chloroacetamide to reduce and cap thiols and disulfide bonds, thereby enhancing accessibility of primary amines for subsequent isotope labeling with heavy (ABP-treated) or light (DMSO-treated) formaldehyde and sodium cyanoborohydride, introducing a dimethylation label at all free amines including free N-termini for quantification. Following sample clean up and trypsinization, heavy and light labeled peptides were combined, and peptides bearing free amines newly formed by trypsin (i.e., arising from internal sequences rather than a protein N-terminus) were depleted by undecanylation with undecanal and sodium cyanoborohydride and passage through a C18 column. LC–MS/MS analysis revealed 94% dimethylation efficiency, with HUNTER increasing the proportion of protein N-terminal peptides >10-fold post-enrichment, with 96% of peptides bearing heavy or light dimethylation, or N-terminal acetylation typical of standard protein N-termini (Figure 5C).

Peptides enriched in DMSO or ABP-treated (i.e., KLK6-inhibited) samples with Log_2_ heavy/light (*H*/*L*) ratios <0.5 or >0.5, respectively, were selected for further consideration since both classes of peptide have the potential to identify KLK6 cleavage sites (Figure 5E). A neo-N-terminal peptide depleted on KLK6 inhibition (i.e., more abundant in the DMSO condition) may be considered a potential KLK6 cleavage site at the P′-side (i.e., toward the protein C-terminus); preferred KLK6 cleavage specificity derived from our positional scanning data (Figure S2) was used to analyze the P-side sequence upstream of each experimentally identified P′ site, to retain those bearing P1, P2, or P3 amino acids compatible with the observed library specificity (88%, 30/34). Although trypsin shares Lys/Arg P1 specificity with KLK6, peptide cleavage sites with P1 lysine are very likely to have occurred endogenously due to KLK6 rather than during proteomic sample preparation because trypsin cleavage at lysines is prevented by dimethylation in the HUNTER protocol. Conversely, N-terminal peptides enriched under ABP treatment may represent the special case of a KLK6 cleavage site proximal to the N-terminus of the protein which have been suppressed by KLK6 inhibition, although the exact cleavage site is not defined because these peptides are standard protein N-termini and are not neo-N termini generated by KLK6. Whole proteome quantitative analysis using heavy and light dimethylation in pre-HUNTER samples demonstrated that overall protein abundance is not significantly altered by ABP treatment, whereas the corresponding HUNTER-enriched N-terminal peptides changed significantly, further supporting the conclusion that these are bona fide KLK6 sites rather than artifacts of changes in protein expression (Figure 5D).

Following this analysis, KLK6 autocleavage sites at positions 79 and 81 were confirmed as hits; these cleavage events have been previously characterized as part of a negative feedback loop,^32^ providing further validation for both bABP **17** (IMP-2352) and the N-terminomic workflow (Tables 2 and S13). Biochemical assays on two putative novel substrates with reported roles in invasion and migration, fibroblast growth factor binding protein (FGFBP1) and heparan sulfate proteoglycan 2 (HSPG2),^44,45^ provided preliminary evidence that these proteins are cleaved by KLK6 in proportion to protease exposure (Figure S14). Western blot data using polyclonal antibodies were consistent with FGFBP1 cleavage at Lys149–Leu150, while inactive KLK6 showed no cleavage under these conditions, and co-incubation with **17** (IMP-2352) inhibited cleavage, confirming activity-dependent proteolysis.

**Table 2:** Putative KLK6 Substrates and Cleavage Sites Identified in the HUNTER Analysis of Capan-2 Conditioned Media^a^

gene	protein name	P4–P1	peptide ID	cleavage position (P1′)	Log_2_(*H*/*L* ratio)
ACT/POTE	actin/POTE	DAPR	AVFPSIVGRPR	29	–0.60
ALDOA	fructose–bisphosphate aldolase A	IAHR	IVAPGKGILAADESTGSIAKR	23	–0.76
C22orf42	uncharacterized protein C22orf42	ARSR	LNEPISSQVLGLLR	237	–1.36
CST3	cystatin-C	KPPR	LVGGPMDASVEEEGVRR	35	–1.26
CST3	cystatin-C	KPPR	LVGGPMDASVEEEGVR	35	–1.41
EIF4A1	eukaryotic initiation factor 4A-I	EVQK	LQMEAPHIIVGTPGR	147	–1.38
EPS8	epidermal growth factor receptor kinase substrate 8	DALR	MISNADPSIPPPPR	201	–1.55
**FGFBP1**	**fibroblast growthfactor-bindingprotein 1**	**SSLK**	**LVSSTLFGNTKPR**	**150**	**–0.92**
FLNA	filamin-A	VKAR	VANPSGNLTETYVQDR	1297	–1.21
FLNB	filamin-B	APLK	IFAQDGEGQR	513	–1.01
GNG12	guanine nucleotide-binding protein subunit gamma-12	-SSK	TASTNNIAQAR	5	–1.30
HSP90A	heat shock protein HSP 90-beta	DPSK	LDSGKELKIDIIPNPQER	65	–0.85
HSPA 1A/1B/6	heat shock 70 kDa protein 1A/1B/6	NVLR	IINEPTAAAIAYGLDR	117	–1.10
HSPA8	heat shock cognate 71 kDa protein	NVLR	IINEPTAAAIAYGLDKKVGAER	172	–0.92
HSPA8	heat shock cognate 71 kDa protein	DTER	LIGDAAKNQVAMNPTNTVFDAKR	50	–1.14
**HSPG2**	**heparan sulfate proteoglycan 2**	**GEAR**	**LVSEDPINDGEWHR**	**4282**	**–0.71**
KLK10	kallikrein-10	MLLK	LARPVVLGPR	143	–1.11
**KLK6**	**kallikrein-6**	**LRQR**	**ESSQEQSSVVR**	**81**	**–2.01**
KLK6	kallikrein-6	RPAK	LSELIQPLPLER	118	–1.51
**KLK6**	**kallikrein-6**	**HNLR**	**QRESSQEQSSVVR**	**79**	**–1.14**
LAMC2	laminin subunit gamma-2	SVSR	LQGVSDQSFQVEEAKR	858	–0.90
LOXL4	lysyl oxidase homolog 4	TKLR	LVGPESKPEEGR	34	–1.29
NME 1/2	nucleoside diphosphate kinase A/B	NCER	TFIAIKPDGVQR	7	–1.03
PCDH1	protocadherin-1	HLYK	LEVGAPYLR	92	–0.68
PLAT	tissue-type plasminogen activator	RGAR	SYQGCSEPR	36	–1.38
PLEC	plectin	GVAR	LSAEAEKVLALPEPSPAAPTLR	1066	–1.18
RARRES1	retinoic acid receptor responder protein 1	SALR	VLAEVQEGR	82	–1.24
ST14	matriptase	TVQR	TQDNSCSFGLHAR	209	–0.92
TKT	transketolase	DQQK	LQALKDTANR	12	–0.57

a Sites of particular interest are highlighted in bold.

## Conclusions

**Figure 6:**
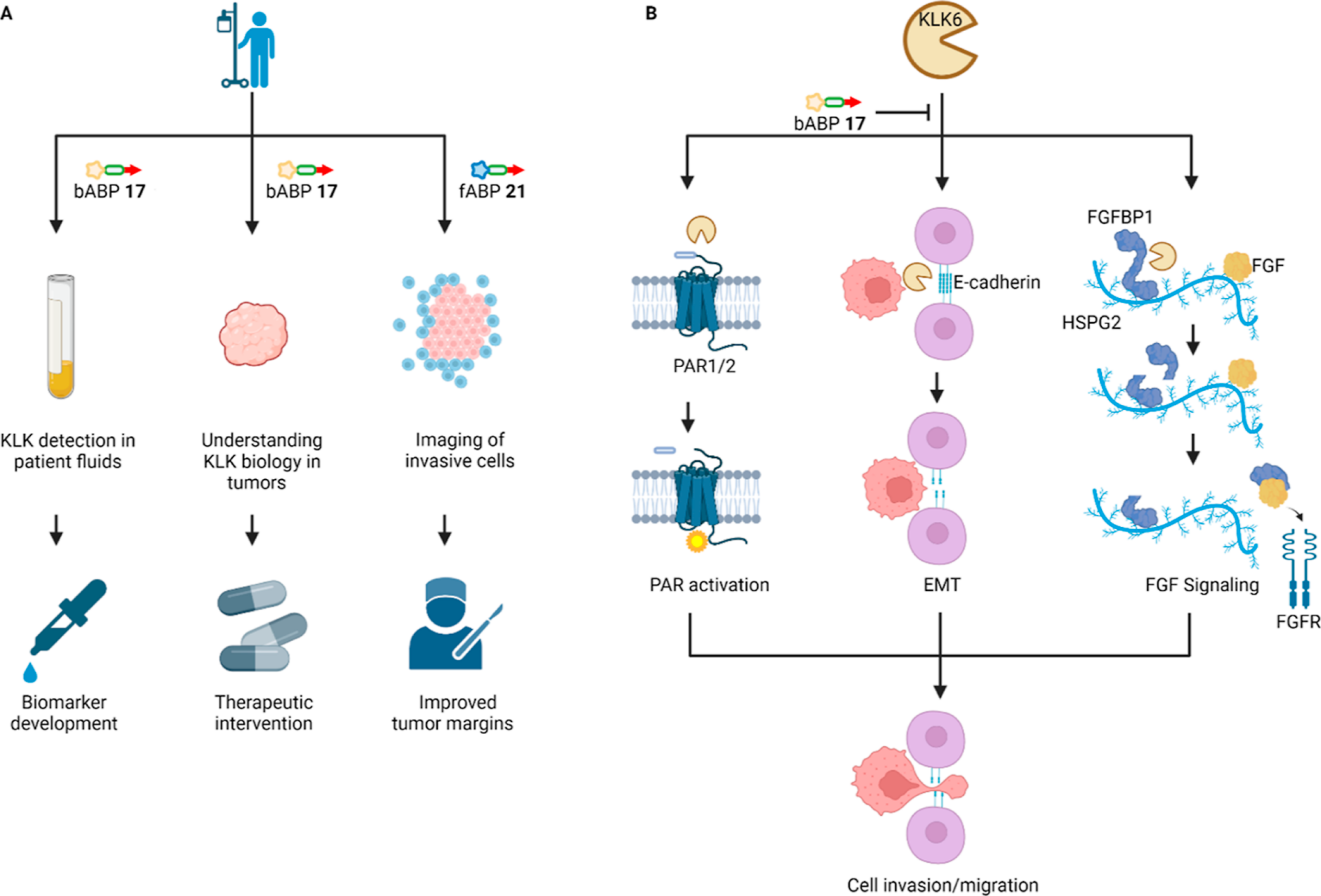
Summary of the roles of KLK6 in PDAC and potential avenues of further research. (A) Potential future clinical applications of the KLK ABP technology. bABP **17** could be used in patient biofluids or tumor samples to further validate KLK6 as a biomarker or therapeutic target,^7^ or fABP **21** used as a probe to image active KLK6, for example, in the context of fluorescence-guided surgery. (B) Potential roles for KLK6-mediated invasion and migration in PDAC, which might be targeted for future therapeutic intervention. In addition to activation of PAR^49,50,53,54^ and cleavage of ECM proteins such as E-cadherin,^51,52^ we hypothesize a further pro-invasion role for the KLK6–FGFBP1 interaction, whereby the heparin-binding domain drives FGFBP1 in close proximity to FGF on the heparan sulfate chain of HSPG2. FGFBP1 may then be cleaved by KLK6, releasing the active domain that interacts with FGF to promote FGF signaling.

Chemical proteomics offers a gold standard approach for quantification of selectivity for ABPs; however, novel probes are often used directly in phenotypic experiments without prior identification of off-targets in the system of interest, as in the case of previously reported KLK6 probes,^25,27,37,40^ reducing confidence in the on-target mode of action. The studies presented here establish bABP **17** (IMP-2352) as the first selective KLK6 ABP, and one of the most thoroughly validated protease ABPs till date, with on-target binding and activity explored through a combination of chemical proteomics, phenotypic assays, X-ray crystallography, and N-terminomics. We envisage a range of future applications for these selective KLK6 ABPs (Figure 6A), including detection of KLK6 activity as a potential biomarker, supporting future validation studies of KLK6 as a target for therapeutic intervention in PDAC and even as in-situ imaging tools (e.g., fABP **21**).

The combination of validated selective probes with HUNTER N-terminomics provides insights into potential roles of KLK6 in PDAC invasion. The KLK6–FGFBP1 interaction is of particular interest due to the roles of FGF signaling in PDAC.^46^ Interestingly, a previous in vivo study in which skin was treated with a carcinogen under conditions leading to tumorigenesis, KLK6 and FGFBP1 were two out of only four proteins identified as upregulated, suggesting that there may be a significant cooperative effect between KLK6 and FGFBP1.^47^ Both FGFBP1 and FGF2 contain heparin-binding domains, leading to the hypothesis that FGF2 and FGFBP1 are driven into a “local reservoir” by heparin binding.^48^ We further hypothesize that removal of the FGFBP1 heparin binding domain by KLK6 may promote FGF2 binding, supported by the previous observation that heparin competes with FGF2 for FGFBP1 binding.^47^ Future studies could investigate the contribution of this specific cleavage event in promoting cell migration, alongside previously hypothesized mechanisms of action for KLK6-dependent proteolysis in the tumor microenvironment, including activation of protease activated receptors (PAR)^49,50^ and cleavage of extracellular matrix proteins such as E-cadherin^51,52^ (Figure 6B).

In conclusion, the optimized KLK6 probes presented here are powerful tools to systematically elucidate the specificity and function of KLK6 both in vitro and in cellulo. The present work offers a framework for future studies on other serine hydrolases, from probe discovery to in-depth optimization and characterization by structural and activity-based proteomics approaches, leading to selective and potent ABPs, and inhibitors and matched controls suitable for driving N-terminomic profiling and phenotypic analyses. This paradigm has the potential to shed light on the cryptic function of currently poorly understood proteases that have been found to be involved in tumor progression.

## Data Availability

The crystal structures have been submitted to the Protein Data Bank under accession code 7QFT and 7QFV. The mass spectrometry proteomics data have been deposited to the ProteomeXchange Consortium via the PRIDE^55^ partner repository with the dataset identifier PXD035111.

## Supporting Information Available

Additional results and information on materials and methods for western blots, chemical proteomics, phenotypic assays, X-ray crystallography, N-terminomics, detailed chemical synthesis for all synthesized compounds, substrate library screening results, chemical structures of amino acids, enzyme inhibition assays, HPLC, HRMS, and NMR spectra (PDF)
