# Supplementary material for: A KLK6 Activity-Based Probe Reveals a Role for KLK6 Activity in Pancreatic Cancer Cell Invasion
Source: J Am Chem Soc. 2022 Nov 22;144(49):22493–504. doi: 10.1021/jacs.2c07378 (PMC9756341; doi:10.1021/jacs.2c07378)
Supplement: Supplementary file 1 — ja2c07378_si_001.pdf [file ja2c07378_si_001.pdf]

A KLK6 activity-based probe reveals a role for KLK6 activity in pancreatic cancer cell invasion

Leran Zhang[1], Scott Lovell[2], Elena De Vita[1], Pravin Kumar Ankush Jagtap[3,4], Daniel Lucy[1], Andrea Goya Grocin[1], Svend Kjær[5], Annabel Borg[5], Janosch Hennig[3,4], Aubry K. Miller[6] and Edward W. Tate[*,1].

(1) Department of Chemistry, Molecular Sciences Research Hub, Imperial College London, London, W12 0BZ, United Kingdom.

(2) Department of Life Sciences, University of Bath, Bath, BA2 7AX, United Kingdom.

(3) Structural and Computational Biology Unit, European Molecular Biology Laboratory, 69117 Heidelberg, Germany

(4) Chair of Biochemistry IV, Biophysical Chemistry, University of Bayreuth, 95447 Bayreuth, Germany

(5) Structural Biology Science Technology Platform, The Francis Crick Institute, NW1 1AT, United Kingdom

(6) Cancer Drug Development Group, German Cancer Research Center (DKFZ), 69120 Heidelberg, Germany

* Correspondence should be addressed to E.W.T. (e.tate@imperial.ac.uk)

Supporting information

Table of Contents

1. Supplementary figures and tables 2

S1 Target discovery experiment 2

S2. Positional scanning combinatorial substrate library screenings 3

S3. Positional scanning combinatorial substrate library amino acid legend 5

S4. Fluorogenic substrate K_M values 6

S5. Probe optimisation K_{obs}/I 6

S6. KLK6 glycosylation experiments 8

S7. Activity of deglycosylated KLK6. 8

S8. Chemical proteomics using the inactive control ABP 9

S9. X-ray crystallography electron density 10

S10. X-ray co-crystal structure of bABP 16 with KLK6 11

S11. Incucyte assays for proliferation and toxicity. 12

S12. Probe treatment in live Capan-2 cells and cell lysate 13

S13. HUNTER substrate profile (positive log(H/L ratio)) 14

S14. In vitro cleavage of FGFBP1 and HSPG2 by KLK6. 16

S15. X-ray crystallography data collection and refinement statistics. Statistics for the highest-resolution shell are shown in parentheses. 17

2. Experimental 19

Materials and methods 19

Synthesis of ABPs 21

Synthesis of purified fluorogenic substrates 23

Synthesis of H_2N-ArgPP 24

Synthesis of H_2N-Phg(4-guan)PP 25

Biological experiments 27

3. Compound Characterisation (Structure, HPLC and HRMS) 34

4. Original gels and blots 63

5. References 66

1. Supplementary figures and tables

S1 Target discovery experiment

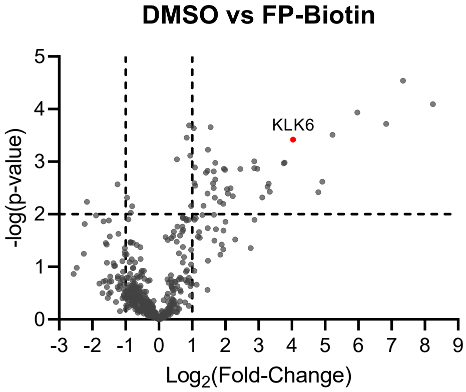

Significantly enriched proteins

AKR1B1	Aldose reductase
ALDOA	Fructose-bisphosphate aldolase A
ANP32A	Acidic leucine-rich nuclear phosphoprotein 32 family member A
ANXA3	Annexin A3
APEH	Acylamino-acid-releasing enzyme
BTD	Biotinidase
CST3	Cystatin-C
CTSC	Dipeptidyl peptidase 1
CTSH	Cathepsin H
DPP4	Dipeptidyl peptidase 4
ESD	S-formylglutathione hydrolase
FAM3C	Protein FAM3C
FDPS	Farnesyl pyrophosphate synthase
GGH	Gamma-glutamyl hydrolase
IAH1	Isoamyl acetate-hydrolyzing esterase 1 homolog
KLK6	Kallikrein-6
LCN2	Neutrophil gelatinase-associated lipocalin
LGALS3BP	Galectin-3-binding protein
LYPLAL1	Lysophospholipase-like protein 1
MDH1	Malate dehydrogenase, cytoplasmic
OVCA2	Ovarian cancer-associated gene 2 protein
PAFAH1B3	Platelet-activating factor acetylhydrolase IB subunit gamma
PFN1	Profilin-1
PLAT	Tissue-type plasminogen activator
PLAU	Urokinase-type plasminogen activator
PREP	Prolyl endopeptidase
PSMA4	Proteasome subunit alpha type-4
PSMA6	Proteasome subunit alpha type-6
PSMB1	Proteasome subunit beta type-1
PSMB4	Proteasome subunit beta type-4
PSMB5	Proteasome subunit beta type-5
PSMB6	Proteasome subunit beta type-6
PSMB7	Proteasome subunit beta type-7
SIAE	Sialate O-acetylesterase
ST14	Suppressor of tumorigenicity 14 protein
TIMP1	Metalloproteinase inhibitor 1
TIMP2	Metalloproteinase inhibitor 2
TPI1	Triosephosphate isomerase
TPP1	Tripeptidyl-peptidase 1
YWHAE	14-3-3 protein epsilon
YWHAG	14-3-3 protein gamma
YWHAZ	14-3-3 protein zeta/delta

Figure S1. Serine hydrolase profile of Capan-2 conditioned media. DMSO or FP-biotin (10 µM) were incubated with conditioned media from Capan-2 cells (300 µg total protein) in technical replicates. The chemical proteomics workflow included a precipitation, a pulldown with NeutrAvidin resin, on-bead trypsin digestion and stage tipping, the peptides were analysed using LC-MS/MS (Label-free quantification). MaxQuant was used to convert the raw mass traces into protein IDs, while Perseus was used for statistics. The volcano plot shows the changes in protein quantification between the DMSO and FP-biotin treated samples vs statistical significance. The significantly enriched proteins (top right section of the volcano plot) are represented in the table.

S2. Positional scanning combinatorial substrate library screenings

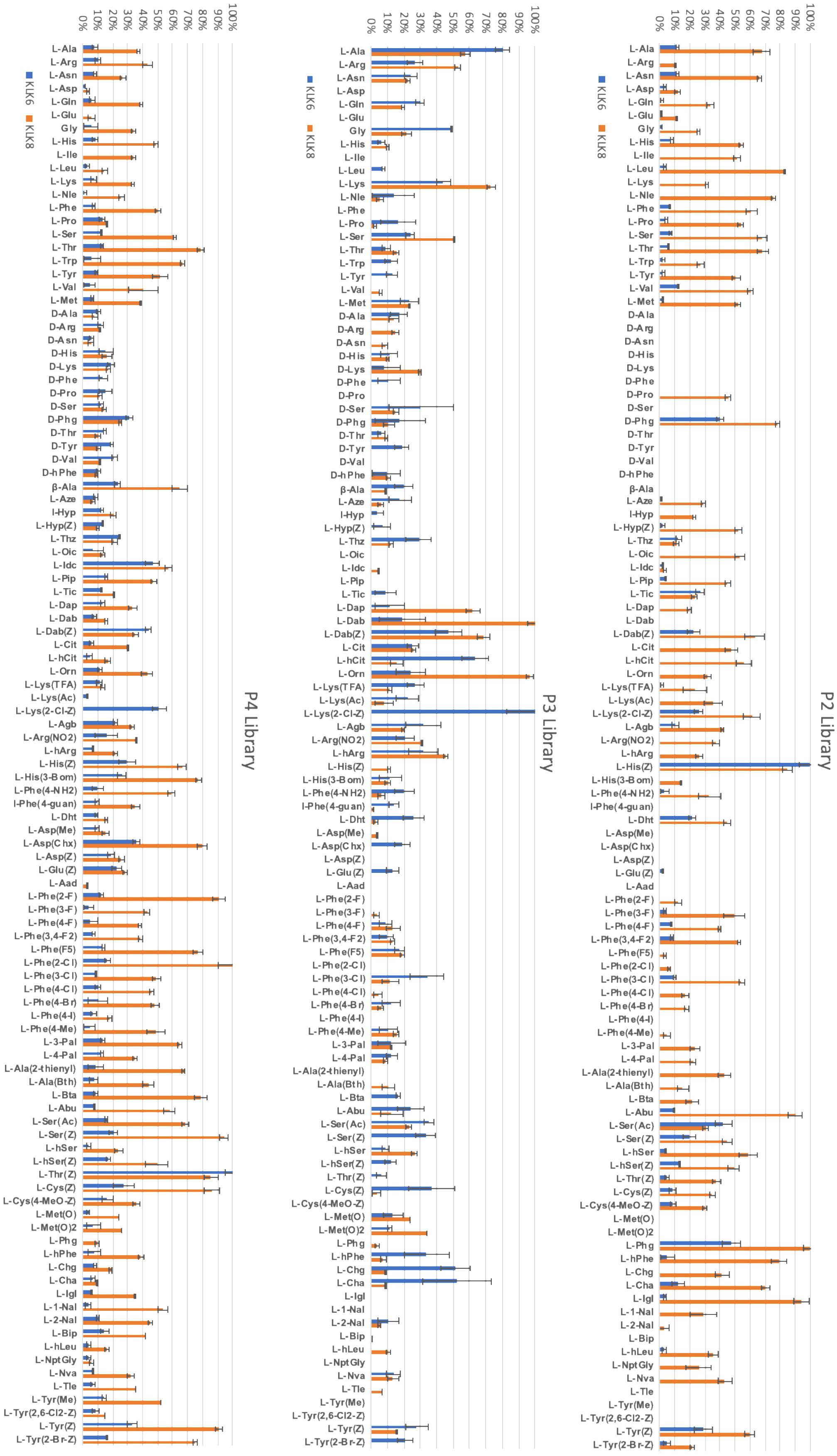

Figure S2. Positional scanning combinatorial substrate library screening of KLK6 (blue) and KLK8 (orange). The P2, P3 and P4 sublibraries were incubated with recombinant human KLK6 and KLK8 in triplicates. Relative fluorescence units (RFU) was measured over time (s) and the initial rate of cleavage for each substrate (RFU/s) was calculated and presented on a bar chart. The substrate with the highest rate for each sublibrary was normalised as 100%. The highest RFU/s value (100%) for each bar chart is as follows: KLK6 P2 = 24,600 RFU/s, KLK6 P3 = 12,700 RFU/s, KLK6 P4 = 15,600 RFU/s, KLK8 P2 = 952 RFU/s, KLK8 P3 = 1640 RFU/s and KLK8 P4 = 762 RFU/s. The Error bars indicate standard deviation between the three replicates.

S3. Positional scanning combinatorial substrate library amino acid legend

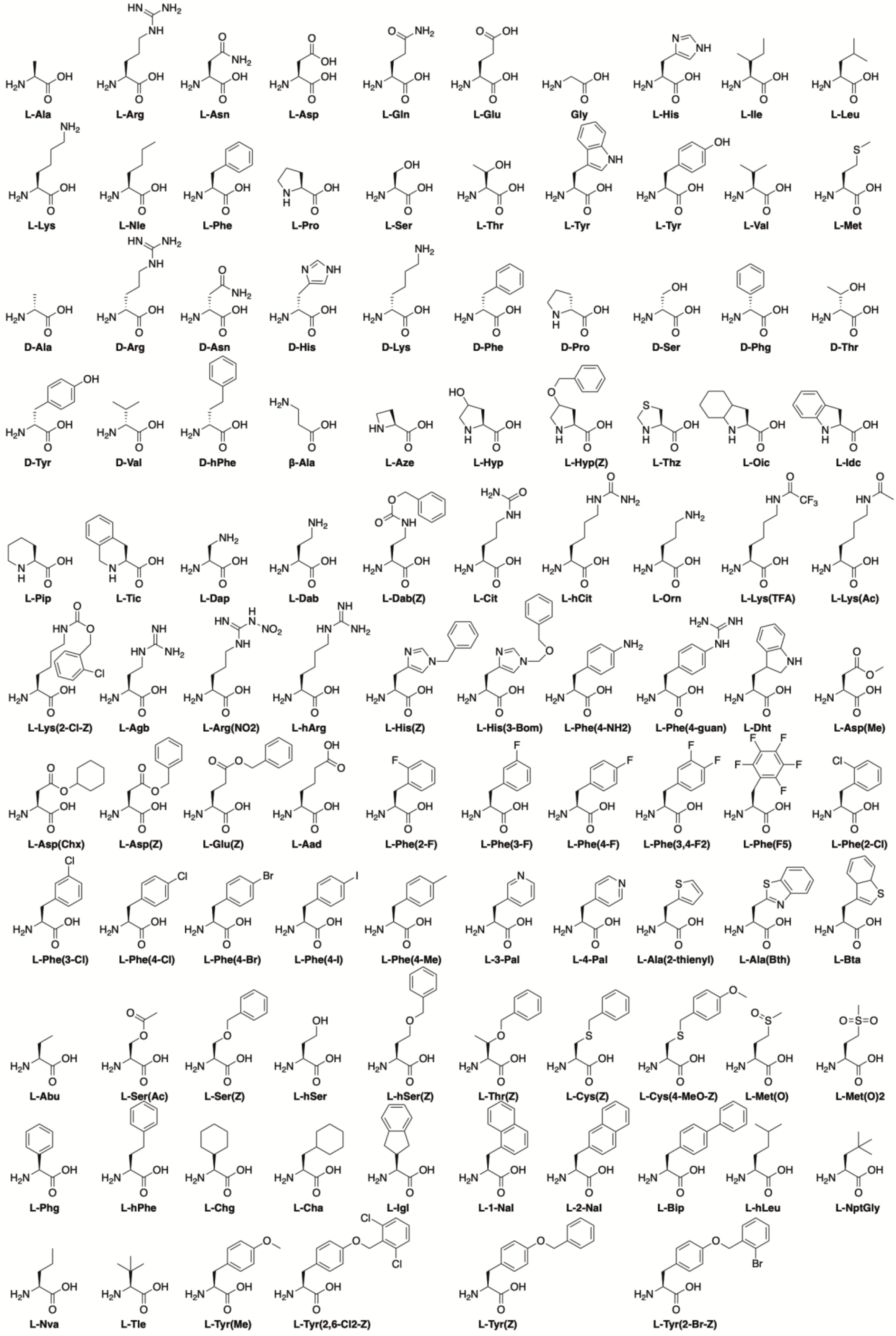

S4. Fluorogenic substrate K_M values

tPA Assays

CAS	Compound Sequence	tPA K_M (μM)	95% CI
**102601-58-1**	Z-GGR-AMC	1200	± 300

uPA Assays

CAS	Compound Sequence	uPA K_M (μM)	95% CI
**102601-58-1**	Z-GGR-AMC	190	± 20

KLK6 Assays

Code	Compound Sequence	KLK6 K_M (μM)	95% CI
**22**	Ac-Thr(Z)-Ala-Phg-R-ACC	86	± 8
**23**	Ac-DPhe-Ser(Z)-Dht-R-ACC	180	± 20

KLK7 Assays

Code	Compound Sequence	KLK7 K_M (μM)	95% CI
**24**	Ac-Lys-His-Leu-Phe-ACC	56	± 8

KLK8 Assays

CAS	Compound Sequence	KLK8 K_M (μM)	95% CI
**65147-04-8**	Boc-VPR-AMC	290	± 20

S5. Probe optimisation k_{obs}/I

tPA Assays

Code	Compound Sequence	tPA k_{obs}/I (M^{-1} s^{-1})	95% CI
**17**	Biotin-Ahx-DPhe-Ser(Z)-Dht-Arg	27	± 4
**21**	Cy5-DPhe-Ser(Z)-Dht-Arg	8	± 1

uPA Assays

Code	Compound Sequence	uPA k_{obs}/I (M^{-1} s^{-1})	95% CI
**17**	Biotin-Ahx-DPhe-Ser(Z)-Dht-Arg	18	± 2
**21**	Cy5-DPhe-Ser(Z)-Dht-Arg	0	-

KLK7 Assays

Code	Compound Sequence	KLK7 k_{obs}/I (M^{-1} s^{-1})	95% CI
**17**	Biotin-Ahx-DPhe-Ser(Z)-Dht-Arg	350	± 90
**21**	Cy5-DPhe-Ser(Z)-Dht-Arg	26	± 4

KLK8 Assays

Code	Compound Sequence	KLK8 k_{obs}/I (M^{-1} s^{-1})	95% CI
**10**	Biotin-Ahx-Thr(Z)-Cha-Dht-Phg(4-guan)	71	± 13
**11**	Biotin-Ahx-DPhe-Cha-Dht-Phg(4-guan)	0	-
**12**	Biotin-Ahx-DHPhe-Cha-Dht-Phg(4-guan)	0	-
**14**	Biotin-Ahx-DPhe-DLys-Dht-Phg(4-guan)	73	± 5
**15**	Biotin-Ahx-DPhe-Cha-Ser(Ac)-Phg(4-guan)	20	± 5
**16**	Biotin-Ahx-DPhe-Cha-Dht-Arg	150	± 9
**17**	Biotin-Ahx-DPhe-Ser(Z)-Dht-Arg	140	± 5
**21**	Cy5-DPhe-Ser(Z)-Dht-Arg	40	± 6

KLK6 Assays

Code	Compound Sequence	KLK6 k_{obs}/I (M^{-1} s^{-1})	95% CI
**1**	Yn-Thr(Z)-Ala-Phg-Phg(4-guan)	220	± 10
**2**	Yn-Thr(Z)-Chg-His(Z)-Phg(4-guan)	30	± 6
**3**	Yn-Thr(Z)-Cha-His(Z)-Phg(4-guan)	46	± 6
**4**	Yn-Asp(Chx)-Cha-His(Z)-Phg(4-guan)	54	± 3
**5**	Yn-Thr(Z)-Ala-Dht-Phg(4-guan)	250	± 10
**6**	Yn-Thr(Z)-Ala-Tyr(Z)-Phg(4-guan)	7	± 2
**7**	Yn-Thr(Z)-Ala-Ser(Z)-Phg(4-guan)	43	± 10
**8**	Yn-Thr(Z)-Ala-Dab(Z)-Phg(4-guan)	11	± 4
**9**	Biotin-Ahx-Thr(Z)-Ala-Dht-Phg(4-guan	220	± 10
**10**	Biotin-Ahx-Thr(Z)-Cha-Dht-Phg(4-guan)	420	± 20
**11**	Biotin-Ahx-DPhe-Cha-Dht-Phg(4-guan	210	± 10
**12**	Biotin-Ahx-DhPhe-Cha-Dht-Phg(4-guan)	140	± 30
**13**	Biotin-Ahx-DPhe-Lys-Dht-Phg(4-guan)	250	± 20
**14**	Biotin-Ahx-DPhe-DLys-Dht-Phg(4-guan)	550	± 30
**15**	Biotin-Ahx-DPhe-Cha-Ser(Ac)-Phg(4-guan)	150	± 10
**16**	Biotin-Ahx-DPhe-Cha-Dht-Arg	1300	± 70
**17**	Biotin-Ahx-DPhe-Ser(Z)-Dht-Ar	11000	± 300
**18**	Biotin-Ahx-DPhe-Ser(Z)-Dht-DArg	0	-
**19**	Yn-DPhe-Ser(Z)-Dht-Arg	3000	± 120
**20**	Yn-DPhe-Ser(Z)-Dht-DArg	0	-
**21**	Cy5-DPhe-Ser(Z)-Dht-Arg	1000	± 100

S6. KLK6 glycosylation experiments

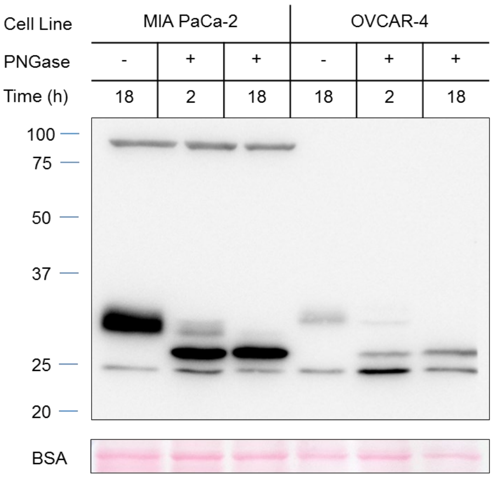

Figure S6. Conditioned media from MIA PaCa-2 and OVCAR-4 cells (10 µg per condition) were incubated with PNGase F (1:20 dilution) at 37 °C for 2 or 18 h. The proteins were analysed by western blot using a KLK6 antibody (Abcam). The glycosylated KLK6 (31 kDa) was deglycosylated by PNGase in a time dependent fashion into a 28 kDa band.

S7. Activity of deglycosylated KLK6

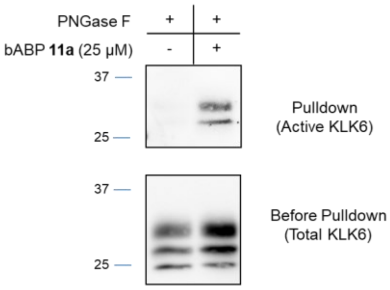

Figure S7. Conditioned media from MIA PaCa-2 cells (200 µg per condition) were incubated with PNGase F (1:100) at 37 °C for 3 h. Next, bABP **11** (25 µM) was added and the sample was shaken at room temperature for 2 h. The labelled proteins were pulled down with magnetic streptavidin and eluted by boiling at 95 °C. 10 µg of sample was taken before pulldown and 10 µg of supernatant was kept for analysis. The proteins were analysed via western blot using a KLK6 antibody (Abcam). The bands in the "before pulldown" and "supernatant" lanes show glycosylated KLK6 (31 kDa), deglycosylated KLK6 (28 kDa) and inactive KLK6 (25 kDa). Both the 31 kDa and 28 kDa bands were pulled down, meaning that these were active. Therefore we can confirm that KLK6 that has been deglycosylated by PNGase retains activity.

S8. Chemical proteomics using the inactive control ABP

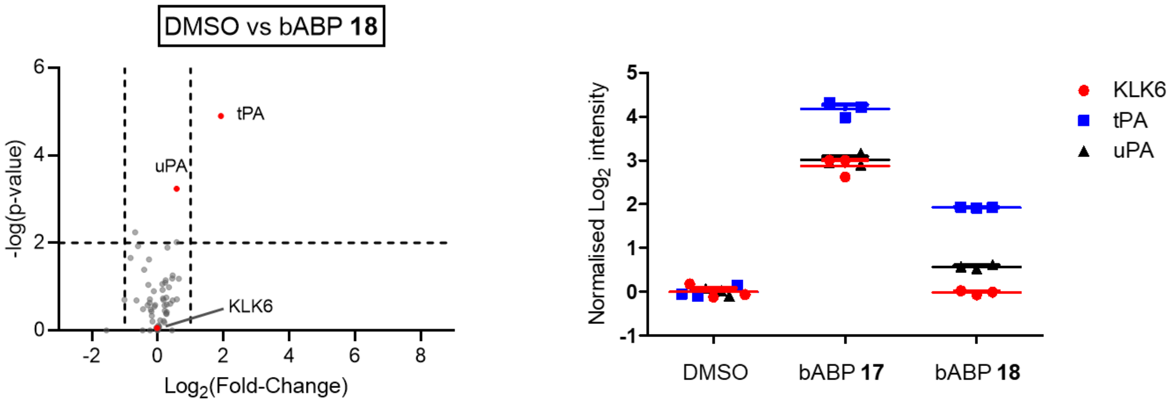

Figure S8. Target analysis of the inactive control **18** using LC-MS/MS. Capan-2 conditioned media was treated with **18** or DMSO for 1 h, followed by a chemical proteomics workflow to enrich for labeled proteins. Samples were analysed on a Thermo Q-Exactive using data-dependent acquisition. Raw data were processed using MaxQuant and Perseus. The volcano plot and graph show the differences in protein quantification between the DMSO and **18** treated conditions. Enrichment was only observed for uPA and tPA, not KLK6.

S9. X-ray crystallography electron density

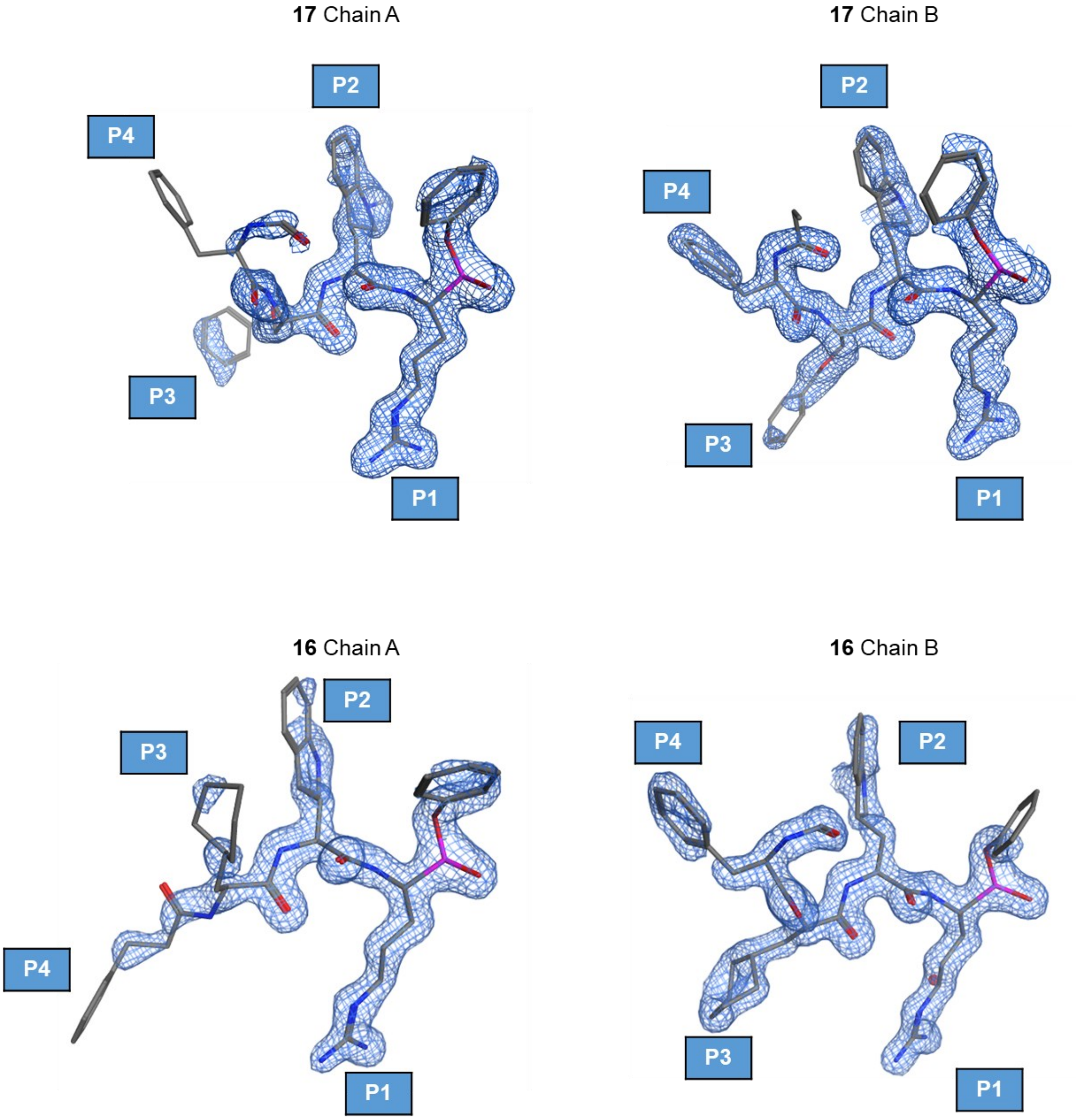

Figure S9. Electron density maps of bABP **16** and bABP **17** from the KLK6 co-crystal structure. Each unit cell contained two KLK6 chains, both with bABP **16** or bABP **17** covalently attached in the active site (chain A and chain B) Carbon atoms are in grey, oxygens are red, nitrogens are blue and the phosphorus is violet. The figure shows 2mFo-DFc maps contoured at 1 sigma.

S10. X-ray co-crystal structure of bABP 16 with KLK6

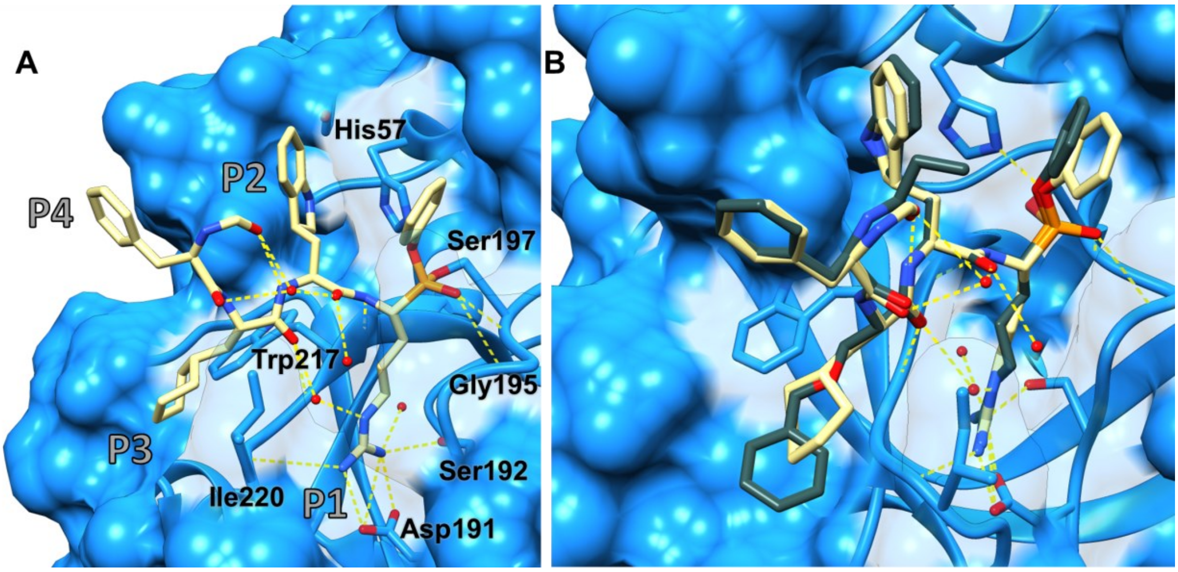

Figure S10. (A) Co-crystal structure of bABP **16** (yellow) with KLK6 (blue) determined to 1.5 Å (Protein data bank (PDB): 7QFT, chain B) illustrating the binding mode of subsites P1–P4 of bABP **16** inside the S1–S4 binding pockets of KLK6. Polar interactions are shown as yellow dotted lines. (B) Superimposition of bABP **17** (green) onto the co-crystal structure of PDB 7QFT, chain B, showing the strong intramolecular network of H-bonds within the backbone chain of both probes. Oxygens, red; Nitrogen, blue; phosphorus, orange; hydrogen bonds, yellow.

S11. Incucyte assays for proliferation and toxicity

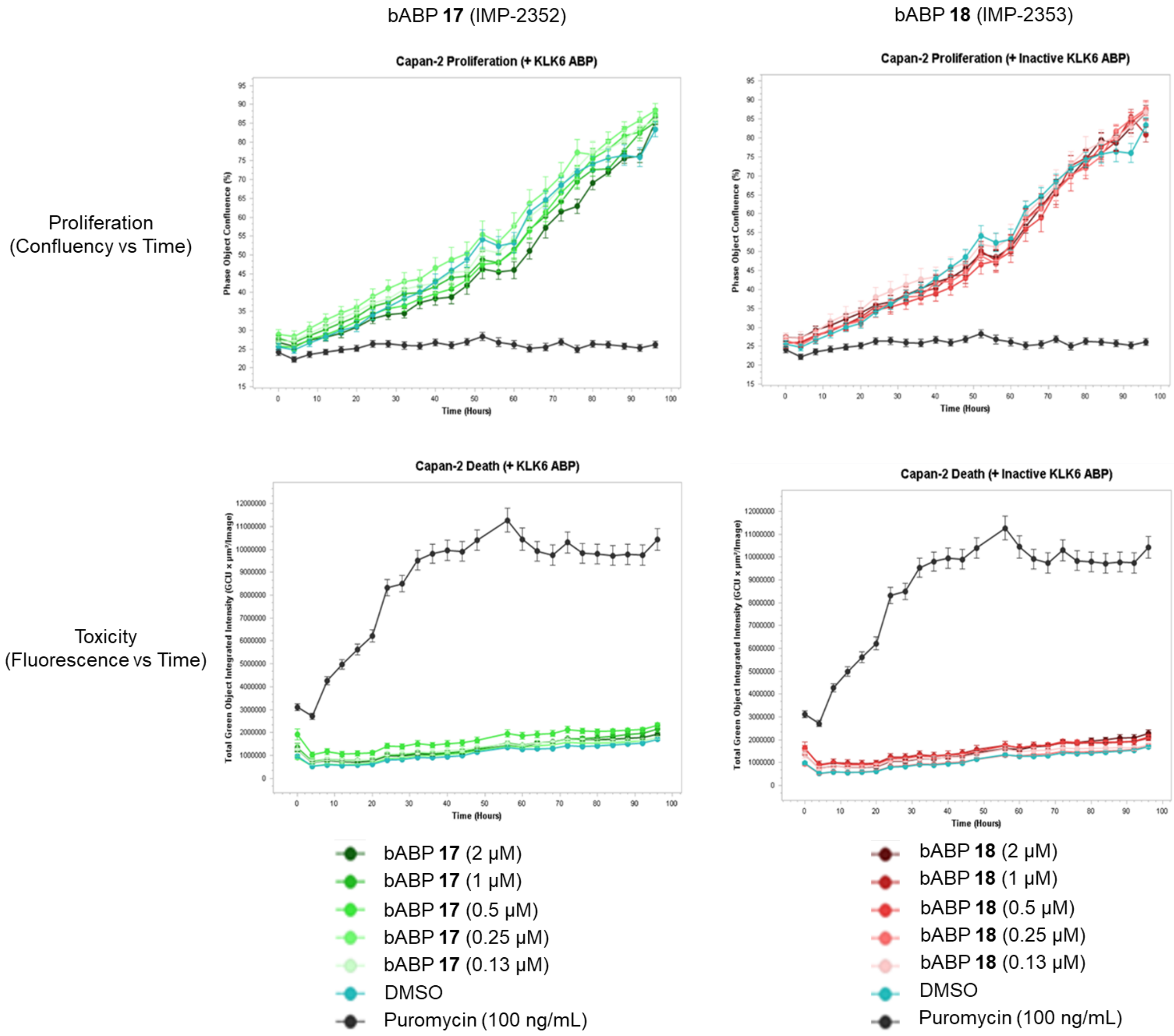

Figure S11. The KLK6 probe **17** and inactive control **18** were tested in Capan-2 cells over five concentrations (2 µM to 0.13 µM) for effects on proliferation (upper row) and toxicity (lower row). Proliferation was quantified using the Incucyte software by measuring percentage confluency. Sytox green was used as a marker for cell death. Cell death was quantified by measuring total green fluorescence of each well. A puromycin control (50 µg/mL) was used and this is represented by the black curve. Neither the KLK6 inhibitor nor the inactive control had any effect on proliferation or cell death. Thus, the compounds were non-toxic and KLK6 inhibition had no effect on proliferation in Capan-2.

S12. Probe treatment in live Capan-2 cells and cell lysate

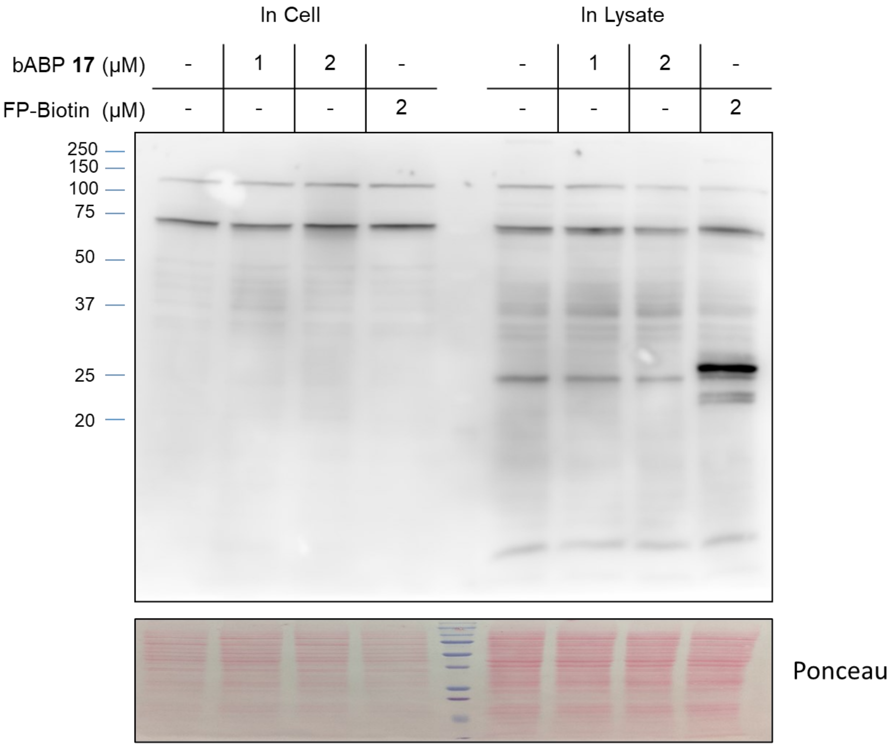

Figure S12. Capan-2 cells were grown to 80% confluency, followed by the addition of bABP **17** or FP-biotin for 2 h. Cells were lysed, boiled with Laemmli buffer and analysed using a NeutrAivdin-HRP western blot (in cell blot). Standard Capan-2 lysate was also treated with the same amounts of bABP **17** and FP-biotin, followed by the same analysis (in lysate blot).

S13. HUNTER substrate profile (positive log(H/L ratio))

Gene	Protein Name	Log_2 (H/L ratio)
ACT/POTE	Actin/POTE	1.36
AIMP1	Aminoacyl tRNA synthase complex-interacting multifunctional protein 1	0.89
AIMP2	Aminoacyl tRNA synthase complex-interacting multifunctional protein 2	0.60
AMHR2	Anti-Muellerian hormone type-2 receptor	2.07
BCCIP	BRCA2 and CDKN1A-interacting protein	0.70
CARHSP1	Calcium-regulated heat stable protein 1	1.36
CCDC6	Coiled-coil domain-containing protein 6	1.72
CLSTN1	Calsyntenin-1	1.24
CST6	Cystatin-M	0.65
CTSH	Cathepsin H	0.54
DCDC5	Doublecortin domain-containing protein 5	0.99
DEK	Protein DEK	1.32
DSG2	Desmoglein-2	0.66
EEF1A1	(Putative) Elongation factor 1-alpha 1	2.04
EEF1D	Elongation factor 1-delta	2.11
EEF1D	Elongation factor 1-delta	2.00
EFHD2	EF-hand domain-containing protein D2	0.83
ENO1	Alpha-enolase	0.66
GAPDH	Glyceraldehyde-3-phosphate dehydrogenase	0.61
H1F0	Histone H1.0	0.76
HIST1H2A	Histone H2A	0.52
HIST1H3A	Histone H3	1.74
HMGA1	High mobility group protein HMG-I/Y	1.28
HNRNPA1	Heterogeneous nuclear ribonucleoprotein A1	0.83
HNRNPK	Heterogeneous nuclear ribonucleoprotein K	1.07
IFT74	Intraflagellar transport protein 74 homolog	0.91
LGALS3BP	Galectin-3-binding protein	0.67
LRP11	Low-density lipoprotein receptor-related protein 11	0.82
MAP4	Microtubule-associated protein 4	1.21
MAPRE1	Microtubule-associated protein RP/EB family member 1	0.68
MCM7	DNA replication licensing factor MCM7	0.67
MVP	Major vault protein	1.27
NSFL1C	NSFL1 cofactor p47	1.36
PFDN2	Prefoldin subunit 2	0.80
PFKP	ATP-dependent 6-phosphofructokinase, platelet type	0.50
PLAU	Urokinase-type plasminogen activator	1.39
PLEC	Plectin	0.80
PPM1G	Protein phosphatase 1G	1.11
RPL13	60S ribosomal protein L13	1.56
RPL13	60S ribosomal protein L13	1.88
RPL15	60S ribosomal protein L15	1.23
RPL17	60S ribosomal protein L17	1.07
RPL24	60S ribosomal protein L24	1.65
RPL30	60S ribosomal protein L30	2.00
RPL31	60S ribosomal protein L31	1.19
RPL4	60S ribosomal protein L4	1.97
RPL6	60S ribosomal protein L6	2.13
RPL7A	60S ribosomal protein L7a	2.24
RPS20	40S ribosomal protein S20	2.22

RPS20	40S ribosomal protein S20	1.45
RPS29	40S ribosomal protein S29	0.89
SGK2	Serine/threonine-protein kinase Sgk2	0.71
SIRT2	NAD-dependent protein deacetylase sirtuin-2	0.71
SRSF2	Serine/arginine-rich splicing factor 2	0.88
ST14	Suppressor of tumorigenicity 14 protein	0.87
TAGLN2	Transgelin-2	1.34
TCOF1	Treacle protein	2.30
TGM2	Protein-glutamine gamma-glutamyltransferase 2	0.54
TIMP1	Metalloproteinase inhibitor 1	2.07
TIMP2	Metalloproteinase inhibitor 2	2.25
TRABD	TraB domain-containing protein	1.33
TUBA 1A/4A/1B/1C/3C/D/3E/8	Tubulin alpha-1A/4A/1B/1C/3C/D/3E/8	2.23
TUBB 4A/4B/8	Tubulin beta-4A/4B/8	0.97
ZNF282	Zinc finger protein 282	0.55

S14. *In vitro* cleavage of FGFBP1 and HSPG2 by KLK6

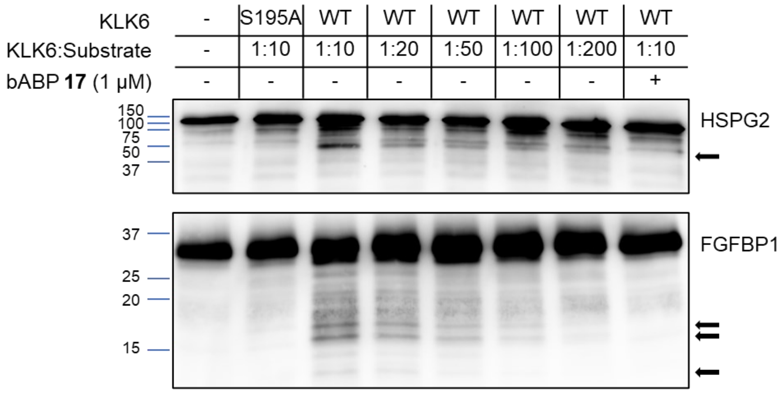

Figure S14. Follow up studies on the hit substrates of interest. Recombinant FGFBP1 and HSPG2 were incubated with KLK6 at different molar ratios for 16 h at 37 °C. Negative controls include DMSO, an inactive KLK6 mutant (S195A) and KLK6 in the presence of bABP **17** (IMP-2352). Arrows represent putative cleavage products made by KLK6. FGFBP1 was sourced as a mixture of two truncated forms (Lys24-Cys234 and Asn29-Cys234), with a C-terminal 10-his tag. Inhibition of KLK6 by bABP **17** prevented cleavage of both substrates. The extent of cleavage is less pronounced compared similar work reported previously on KLK7 and KLK14[1,2], which may be due to low rates of cleavage, differences in enzyme stability, visualisation sensitivity or the buffer system used.

S15. X-ray crystallography data collection and refinement statistics. Statistics for the highest-resolution shell are shown in parentheses.

	KLK6 + **17**	KLK6 + **16**
**Wavelength**	0.97	0.97
**Resolution range**	36.27 - 1.56 (1.62 - 1.56)	64.36 - 1.47 (1.52 - 1.47)
**Space group**	P 1	P 1
**Unit cell**	38.43 40.08 65.21 82.08 84.95 70.87	38.37 39.97 65.07 81.90 84.95 70.71
**Total reflections**	170350 (16327)	198302 (19538)
**Unique reflections**	49455 (4689)	58208 (5062)
**Multiplicity**	3.4 (3.5)	3.4 (3.4)
**Completeness (%)**	95.70 (90.66)	94.68 (83.38)
**Mean I/sigma(I)**	7.63 (1.05)	9.43 (1.46)
**Wilson B-factor**	18.30	16.13
**R-merge**	0.1 (1.11)	0.083 (1.12)
**R-meas**	0.12 (1.311)	0.099 (1.33)
**R-pim**	0.063 (0.69)	0.05 (0.71)
**CC1/2**	0.99 (0.38)	0.99 (0.46)
**CC***	0.99 (0.74)	0.99 (0.80)
**Reflections used in**	49412 (4689)	57643 (5061)
**Reflections used for R-free**	2470 (235)	2881 (252)
**R-work**	0.18 (0.29)	0.17 (0.35)
**R-free**	0.21 (0.34)	0.20 (0.44)
**CC(work)**	0.97 (0.64)	0.97 (0.62)
**CC(free)**	0.95 (0.64)	0.96 (0.44)
**Number of non-hydrogen**	3842	3926
**macromolecules**	3360	3377
**ligands**	118	109

**solvent**	364	440
**Protein residues**	444	444
**RMS(bonds)**	0.007	0.01
**RMS(angles)**	1.11	1.40
**Ramachandran favored (%)**	98.64	98.86
**Ramachandran allowed (%)**	1.14	1.14
**Ramachandran outliers (%)**	0.23	0.00
**Rotamer outliers (%)**	0.00	0.00
**Clashscore**	3.29	5.20
**Average B-factor**	22.90	20.80
**macromolecules**	21.46	19.04
**ligands**	35.05	31.69
**solvent**	32.27	31.58
**Number of TLS groups**	16	

2. Experimental

Materials and methods

Reagents were obtained from VWR, Sigma Aldrich, Sartorius, Fluorochem, AGTC, Novabiochem, TCI, Alfa Aesar, Iris Biotech, Thermofisher and Bachem. ^1H and ^{13}C NMR spectra were measured on 400 MHz Bruker AV instruments. Cells were obtained directly from the ATCC. A Waters LC-MS system (2767 sample manager, 2998 UV detector and 3100 mass detector) was used for the analysis and purification of peptides. The analytical column was an Xbridge C_{18} 4.6 mm x 100 mm. The preparative column used to purify the peptides was an Xbridge C_{18} 19 mm x 100 mm. For analytical LC-MS, the flow rate was 1.2 mL/min, while preparative runs were at 20 mL/min. Injection volumes were 20 µL for analytical runs and 1 to 5 mL for preparative runs. Solvent gradients started at 5:95 MeCN:H_2O (for substrates) or 20:80 MeCN:H_2O (for probes), finishing at 98:2 MeCN:H_2O in 10 mins. This was followed by 98:2 MeCN:H_2O wash for 3 mins and re-equilibration of the column to the starting eluent for 5 mins. All solvents contained an additive (0.1% formic acid). High resolution mass was recorded on a Micromass Autospec Premier through the Imperial College London mass spectrometry services. Active recombinant FGFBP1 (Code 1593-FB), HSPG2 (Code 2364-ER), uPA (Code 1310-SE), tPA (Code 7449-SE) and KLK8 (Code 2025-SE) were obtained from BioTechne. KLK6 was obtained from the group of Aubry Miller (DKFZ) and KLK7 was obtained from the group of Judith Clements (QUT). The manufacturer's recommended buffers were used with uPA and tPA but not KLK8. The KLK6 assay buffer was as follows: 50 mM Tris 150 mM NaCl, 1 mM EDTA, 0.05% Tween20, pH 7.5. The KLK7/8 buffer was as follows: 100 mM Na_2PO_4, 0.01% Tween20, pH 8.5. Fluorescence measurements were measured using an Envision plate reader. Fluorescence data was processed using Graphpad Prism 8. Cell proliferation and toxicity assays were performed using a Sartorius Incucyte. Western blots were imaged using a LAS4000 and in-gel fluorescence results were obtained using a Typhoon 9000.

General methods for peptide preparation

Peptides were prepared using standard fluorenylmethoxycarbonyl (Fmoc) solid phase peptide synthesis. Reactions were monitored using the ninhydrin test. All fluorogenic substrates (combinatorial and purified substrates) were synthesised on rink amide resin following a procedure published by Drag and coworkers[3]. Fluorogenic substrates in the library were dissolved in DMSO at 10 mM based on the average molecular weight of the 400 peptide sequences per sample. Optimised substrates were purified using preparative HPLC-MS (5-95% MeCN gradient). Inhibitors and probes were synthesised on 2-chlorotrityl chloride resin.

Loading onto 2-chlorotrityl resin. 70 mg of resin was used per peptide. 2-Chlorotrityl chloride resin (1.42 mmol/g loading) was swelled in dry DCM for 5 min. The DCM was removed, followed by the addition of the appropriate fmoc-protected amino acid for the P2 position (5 eq). The mixture was dissolved in DCM (1 mL per 70 mg resin) and the loading reaction was activated with DIPEA (10 eq) The reaction mixture was agitated for 2 h. The solvent was removed, and unreacted sites were capped with the addition of MeOH and 1 h agitation. The resin was washed with DMF (3x), DCM (3x), MeOH (3x) and Et_2O (3x) and desiccated. Resin loading was determined by adding 1 mg of resin to 20%

piperidine in DMF (3 mL) and leaving for 20 min. The absorbance at 290 nm was recorded and the loading was determined with the following equation, where A is absorbance and m is the mass of the resin in mg.

$$\mathbf{loading}\left(\frac{\mathbf{mmol}}{\mathbf{g}}\right)=\frac{\mathbf{A_{290nm}}}{(\mathbf{m_{resin}})(\mathbf{1.65})}$$

Coupling of amino acids. The resin with a preloaded P2 fmoc-protected amino acid was swollen in DMF. The fmoc group was removed with the addition of 20% piperidine in DMF (2 x 10 minutes). The resin was washed with DMF (3x), DCM (3x) and DMF (3x). The next (P3 position) fmoc-protected amino acid (5 eq) was premixed in a falcon tube with HATU (5 eq) and DIPEA (10 eq) in DMF (1.5 mL per 70 mg resin). The mixture was added into the resin, followed by a 1 h agitation. The solution was drained, and the resin was washed with DMF (3x), DCM (3x) and DMF (3x). The coupling procedure was repeated to add the P4 position, linkers and tags.

Cleavage from resin. The resin was swollen in DCM. The peptide was cleaved from the resin using a solution of HFIP (25% in DCM) and agitation for 2 h. The solvent was removed through a stream of nitrogen and the peptide was precipitated using cold diethyl ether. The sample was centrifuged (4000 rpm, 3 min) and the supernatant was removed. The pellet was redissolved in a 2:1 solution of MeCN:H_2O (3 mL) and freeze dried overnight, yielding the desired peptide.

Coupling of the warhead to the peptide. The warhead (10 mg, 1.2 eq) was weighed into a small glass container. In a separate container, the peptide (1 eq) was preactivated with HATU (1.1 eq) and collidine (5 eq) in DMF (1 mL). The activated peptide solution was added into the warhead and the reaction was stirred at room temperature for 3 h. Reaction progress was monitored by LC-MS. The DMF was removed using a stream of nitrogen and the protecting groups were removed through the addition of a solution (1 mL) containing 95% TFA, 2.5% triisopropylsilane and 2.5% H_2O. The reaction was stirred at room temperature for 1 h. The solvent was removed using a stream of nitrogen and the crude product was purified using preparative HPLC-MS (20%-98% MeCN gradient). For fully optimised probes and inhibitors, the two diastereoisomers corresponding to the warhead were separated during HPLC, yielding an active compound and an inactive control.

Click reaction to synthesise fluorescent ABPs. An ABP with a warhead, specificity region and alkyne linker was synthesised using the above procedure. A stock solution of $CuSO_4$ (1 mL, 7.5 eq.) and a stock solution of sodium ascorbate (1 mL, 7.5 eq.) were degassed using nitrogen. The alkyne-ABP (1 eq.) and azido-fluorophore (1 eq.) were dissolved in DMF (800 µL) and degassed using nitrogen. 100 µL of the $CuSO_4$ and sodium ascorbate stocks were added into the reaction mixture, followed by stirring at RT for 16 h to give the final equivalents of $CuSO_4$ and sodium ascorbate at 0.75 eq. each. The solvent was removed using a stream of nitrogen and the fluorophore-tagged ABP was purified using preparative HPLC-MS (20%-98% MeCN gradient).

Synthesis of ABPs

Yn-Thr(Z)-Ala-Phg-Phg(4-guan)-DPP. 1 was obtained *via* the general peptide synthesis method at 0.1 mmol scale (yield 1.5 mg). HRMS (ESI *m/z*) calculated for $C_{47}H_{51}N_7O_8P$ $[M+H]^+$ 872.3537, found 872.3532.

Yn-Thr(Z)-Chg-His(Z)-Phg(4-guan)-DPP. 2 was obtained via the general peptide synthesis method at 0.1 mmol scale (yield 1.1 mg). HRMS (ESI *m/z*) calculated for $C_{57}H_{65}N_9O_8P$ $[M+H]^+$ 1034.4694, found 1034.4728.

Yn-Thr(Z)-Cha-His(Z)-Phg(4-guan)-DPP. 3 was obtained via the general peptide synthesis method at 0.1 mmol scale (yield 4.6 mg). HRMS (ESI *m/z*) calculated for $C_{58}H_{67}N_9O_8P$ $[M+H]^+$ 1048.4850, found 1048.4894.

Yn-Asp(Chx)-Cha-His(Z)-Phg(4-guan)-DPP. 4 was obtained via the general peptide synthesis method at 0.1 mmol scale (yield 1.2 mg). HRMS (ESI *m/z*) calculated for $C_{57}H_{69}N_9O_9P$ $[M+H]^+$ 1054.4956, found 1054.4943.

Yn-Thr(Z)-Ala-Dht-Phg(4-guan)-DPP. 5 was obtained via the general peptide synthesis method at 0.1 mmol scale (yield 2.2 mg). HRMS (ESI *m/z*) calculated for $C_{50}H_{56}N_8O_8P$ $[M+H]^+$ 927.3959, found 927.3973.

Yn-Thr(Z)-Ala-Tyr(Z)-Phg(4-guan)-DPP. 6 was obtained via the general peptide synthesis method at 0.1 mmol scale (yield 1.8 mg). HRMS (ESI *m/z*) calculated for $C_{55}H_{59}N_7O_9P$ $[M+H]^+$ 992.4112, found 992.4139.

Yn-Thr(Z)-Ala-Ser(Z)-Phg(4-guan)-DPP. 7 was obtained via the general peptide synthesis method at 0.1 mmol scale (yield 1.5 mg). HRMS (ESI *m/z*) calculated for $C_{49}H_{55}N_7O_9P$ $[M+H]^+$ 916.3799, found 916.3807.

Yn-Thr(Z)-Ala-Dab(Z)-Phg(4-guan)-DPP. 8 was obtained via the general peptide synthesis method at 0.1 mmol scale (yield 2.0 mg). HRMS (ESI *m/z*) calculated for $C_{51}H_{58}N_8O_{10}P$ $[M+H]^+$ 973.4014, found 973.4023.

Biotin-Ahx-Thr(Z)-Ala-Dht-Phg(4-guan)-DPP. 9 was obtained via the general peptide synthesis method at 0.1 mmol scale (yield 6 mg). HRMS (ESI *m/z*) calculated for $C_{61}H_{77}N_{11}O_{10}PS$ $[M+H]^+$ 1186.5313, found 1186.5330.

Biotin-Ahx-Thr(Z)-Cha-Dht-Phg(4-guan)-DPP. 10 was obtained via the general peptide synthesis method at 0.1 mmol scale (yield 3.2 mg). HRMS (ESI *m/z*) calculated for $C_{67}H_{87}N_{11}O_{10}PS$ $[M+H]^+$ 1268.6096, found 1268.6115.

Biotin-Ahx-DPhe-Cha-Dht-Phg(4-guan)-DPP. 11 was obtained via the general peptide synthesis method at 0.1 mmol scale (yield 4.3 mg). HRMS (ESI *m/z*) calculated for $C_{65}H_{83}N_{11}O_9PS$ $[M+H]^+$ 1224.5834, found 1224.5854.

Biotin-Ahx-DhPhe-Cha-Dht-Phg(4-guan)-DPP. 12 was obtained via the general peptide synthesis method at 0.1 mmol scale (yield 1.9 mg). HRMS (ESI *m/z*) calculated for $C_{66}H_{85}N_{11}O_9PS$ $[M+H]^+$ 1238.5990, found 1238.5977.

Biotin-Ahx-DPhe-Lys-Dht-Phg(4-guan)-DPP. 13 was obtained via the general peptide synthesis method at 0.1 mmol scale (yield 8.7 mg). HRMS (ESI *m/z*) calculated for $C_{62}H_{79}N_{12}O_9NaPS$ $[M+Na]^+$ 1221.5449, found 1221.5450.

Biotin-Ahx-DPhe-Dlys-Dht-Phg(4-guan)-DPP. 14 was obtained via the general peptide synthesis method at 0.1 mmol scale (yield 3.4 mg). HRMS (ESI *m/z*) calculated for $C_{62}H_{80}N_{12}O_9PS$ $[M+H]^+$ 1199.5630, found 1199.5641.

Biotin-Ahx-DPhe-Cha-Ser(Ac)-Phg(4-guan)-DPP. 15 was obtained via the general peptide synthesis method at 0.1 mmol scale (yield 5.1 mg). HRMS (ESI *m/z*) calculated for $C_{59}H_{78}N_{10}O_{11}PS$ $[M+H]^+$ 1165.5304, found 1165.5309.

Biotin-Ahx-DPhe-Cha-Dht-Arg-DPP. 16 was obtained via the general peptide synthesis method at 0.1 mmol scale (yield 5.7 mg). HRMS (ESI *m/z*) calculated for $C_{62}H_{85}N_{11}O_9PS$ $[M+H]^+$ 1190.5990, found 1190.6025.

Biotin-Ahx-DPhe-Ser(Z)-Dht-Arg-DPP. 17 was obtained via the general peptide synthesis method at 0.1 mmol scale (yield 5.3 mg). HRMS (ESI *m/z*) calculated for $C_{63}H_{81}N_{11}O_{10}PS$ $[M+H]^+$ 1214.5626, found 1214.5638.

Biotin-Ahx-DPhe-Ser(Z)-Dht-DArg-DPP. 18 was obtained via the general peptide synthesis method at 0.1 mmol scale (yield 6.7 mg). HRMS (ESI *m/z*) calculated for $C_{63}H_{81}N_{11}O_{10}PS$ $[M+H]^+$ 1214.5626, found 1214.5629.

Yn-DPhe-Ser(Z)-Dht-Arg-DPP. 19 was obtained via the general peptide synthesis method at 0.1 mmol scale (yield 5 mg). HRMS (ESI *m/z*) calculated for $C_{52}H_{60}N_8O_8P$ $[M+H]^+$ 955.4272, found 955.4285.

Yn-DPhe-Ser(Z)-Dht-DArg-DPP. 20 was obtained via the general peptide synthesis method at 0.1 mmol scale (yield 4 mg). HRMS (ESI *m/z*) calculated for $C_{52}H_{60}N_8O_8P$ $[M+H]^+$ 955.4272, found 955.4318.

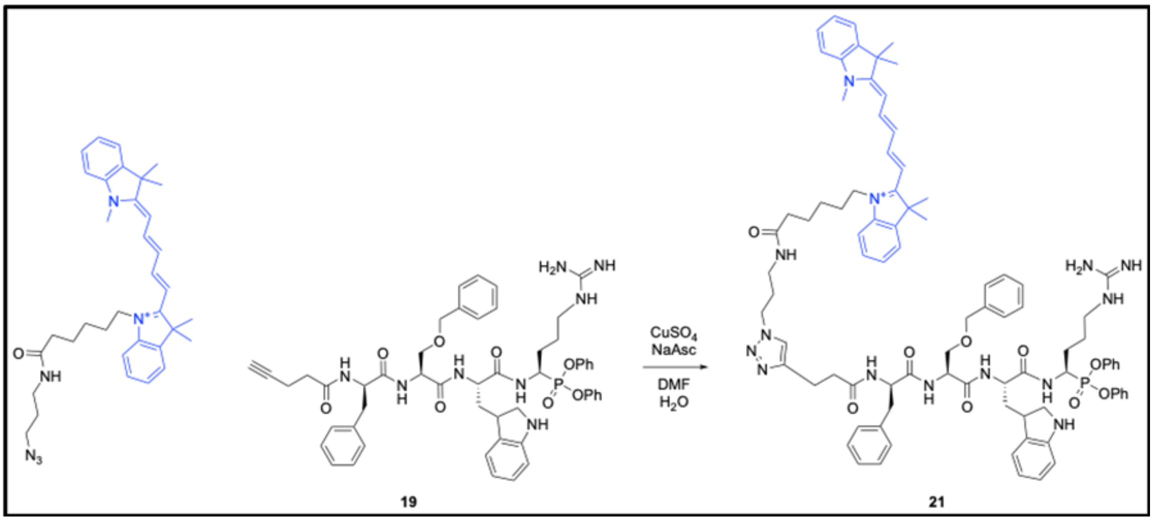

Scheme 1. Copper catalysed azide-alkyne cycloaddition reaction for the synthesis of KLK6_fABP **21**.

Cy5-DPhe-Ser(Z)-Dht-Arg-DPP. 21 was obtained from **19** and azido-Cy5 with the click reaction procedure (yield 2 mg). HRMS (ESI *m/z*) calculated for $C_{87}H_{105}N_{14}O_9P$ $[M+H]^+$ 1520.7916, found 1520.7897.

Synthesis of purified fluorogenic substrates

Ac-Thr(Z)-Ala-Phg-Arg-ACC. 22 was obtained via the general peptide synthesis method at 0.03 mmol scale (yield 3.9 mg). HRMS (ESI, *m/z*) calculated for $C_{41}H_{50}N_9O_9$ [M+H]+ 812.3731, found 812.3760.

Ac-DPhe-Ser(Z)-Dht-Arg-ACC. 23 was obtained via the general peptide synthesis method at 0.03 mmol scale (yield 7 mg). HRMS (ESI, *m/z*) calculated for $C_{49}H_{57}N_{10}O_9$ [M+H]+ 929.4310, found 929.4326.

Ac-Lys-His-Leu-Phe-ACC. 24 was obtained via the general peptide synthesis method at 0.03 mmol scale (yield 7.5 mg). HRMS (ESI, *m/z*) calculated for $C_{40}H_{52}N_9O_8$ [M+H]+ 786.3939, found 786.3947.

Synthesis of H_2N-ArgPP

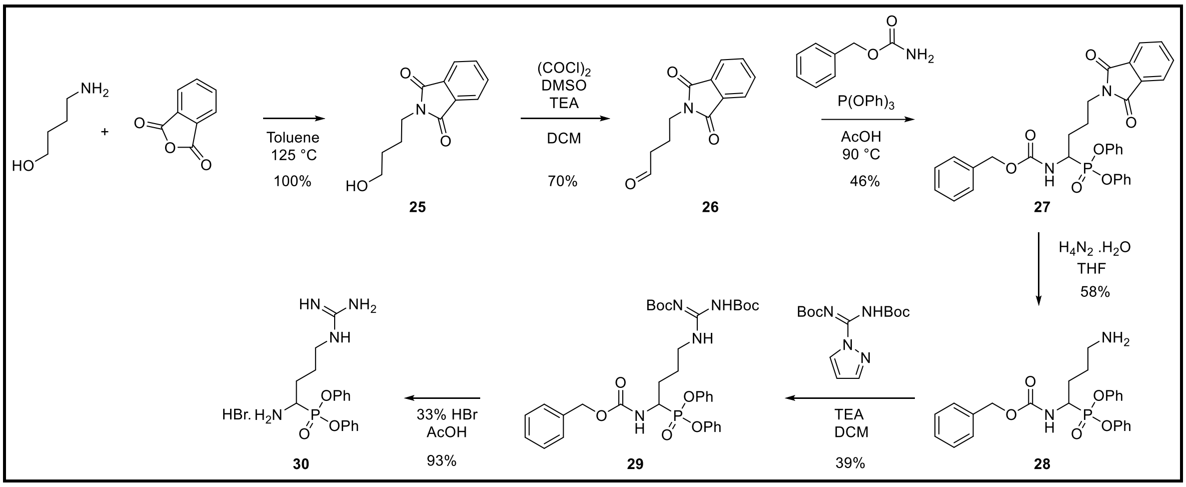

2-(4-hydroxybutyl)isoindoline-1,3-dione (25). To a solution of phthalic anhydride (4.5 g, 30.5 mmol, 1 eq.) in toluene (100 mL) was added 4-aminobutan-1-ol (2.8 mL, 30.5 mmol, 1 eq.) and triethylamine (4.2 mL, 30.5 mmol, 1 eq.) before heating to 125 °C for 3 h. The organic solvents were removed under reduced pressure to afford **25** as a colourless oil (6.7 g, 30.5 mmol, 100%). ^{1}H NMR (400 MHz, $CDCl_3$) δ 7.91 – 7.82 (m, 2H), 7.80 – 7.71 (m, 2H), 3.83 – 3.66 (m, 4H), 1.85 – 1.78 (m, 2H), 1.70 – 1.60 (m, 2H). The analytical data is consistent that reported in the literature[4].

4-(1,3-dioxoisoindolin-2-yl)butanal (26). To a solution of $(COCl)_2$ (2.34 mL, 27.4 mmol, 2 eq.) in DCM (102 mL) was added a solution of DMSO (3.9 mL, 54.8 mmol, 4 eq.) in DCM (27 mL) at -78 C over a period of 30 min. After 5 mins a solution of 2-(4-hydroxybutyl)isoindoline-1,3-dione (3 g, 13.7 mmol, 1 eq.) in DCM (27 mL) was added dropwise over 30 mins. After a further 40 mins, triethylamine (11.4 mL, 82.2 mmol, 6 eq.) was added dropwise over 10 min and the reaction was warmed to 0 °C for 1 h. The reaction was quenched with water (70 mL) and the organic layer was isolated. The organic layer was washed with water (2 × 50 mL) and brine (1 × 50 mL). The organics were dried ($MgSO_4$), filtered and removed under reduced pressure to yield **26** as a colourless liquid (2.1 g, 9.6 mmol, 70%). ^{1}H NMR (400 MHz, Chloroform-d) δ 9.76 (s, 1H), 7.87 – 7.77 (m, 2H), 7.71 (dd, J = 5.4, 3.0, 2H), 3.73 (t, *J* = 6.8, 2H), 2.53 (t, *J* = 7.2, 2H), 2.00 (p, *J* = 7.0, 2H). The analytical data is consistent that reported in the literature[4].

Benzyl (4-(1,3-dioxoisoindolin-2-yl)-1-(diphenoxyphosphoryl)butyl)carbamate (27). Benzyl carbamate (1.04 g, 6.9 mmol, 1 eq.), 4-(1,3-dioxoisoindolin-2-yl)butanal (1.5 g, 6.9 mmol, 1 eq.) and triphenyl phosphite (1.5 mL, 6.9 mmol, 1 eq.) were dissolved in acetic acid (35 mL) and stirred at 90 °C for 2 h. The solvent was removed under reduced pressure and the compound was purified via flash column chromatography (1.87 g, 3.2 mmol, 46%) to afford **27** as a white powder. ^{1}H NMR (400 MHz, $CDCl_3$) δ 7.83 (dd, *J* = 5.5, 3.1, 2H), 7.71 (dd, *J* = 5.5, 3.1, 2H), 7.38 – 7.00 (m, 15H), 5.19 – 5.01 (m, 2H), 4.61 – 4.47 (m, 1H), 3.74 (t, *J* = 6.8, 2H), 2.14 – 1.76 (m, 4H). The analytical data is consistent that reported in the literature[4].

Benzyl (4-amino-1-(diphenoxyphosphoryl)butyl)carbamate (28). To a solution of benzyl (4-(1,3-dioxoisoindolin-2-yl)-1-(diphenoxyphosphoryl)butyl)carbamate (1.86 g, 3.2 mmol, 1 eq.) in THF (30 mL) was added hydrazine (310 µL, 6.4 mmol, 2 eq.). The reaction was stirred at RT for 20 h. The precipitate was filtered away and discarded. The solvent was removed under reduced pressure. The residue was redissolved in $CHCl_3$, washed with brine, dried with $MgSO_4$, filtered and concentrated under reduced pressure to yield **28** (0.85 g, 1.87 mmol, 58%) as a white powder. The crude compound was used without further purification.

Diphenyl N-benzyloxycarbonylamino-(3-(*N,N'*-bis(tert-butyloxycarbonyl)guanyl)propyl) - methane-phosphonate (29). To a solution of *N,N'*-Di-Boc-1H-pyrazole-1-carboxamidine (580 mg, 1.87 mmol, 1 eq.) and Benzyl (4-amino-1-(diphenoxyphosphoryl)butyl)carbamate (850 mg, 1.87 mmol, 1 eq.) in DCM (20 mL) was added triethylamine (521 µL, 3.74 mmol, 2 eq.). The reaction was stirred at RT for 16 h. The solvent was removed under reduced pressure and the residue was redissolved in EtOAc. The solution was washed with 1N HCl, saturated $NaHCO_3$ and brine. The organic layer was dried with $MgSO_4$, filtered and concentrated under reduced pressure. Purification by flash column chromatography afforded **29** (510 mg, 0.73 mmol, 39%) as a white powder. ^{1}H NMR (400 MHz, $CDCl_3$) δ 11.48 (s, 1H), 8.37 (s, 1H), 7.40 – 7.02 (m, 15H), 5.23 – 5.03 (m, 2H), 4.52 (m, 1H), 3.63 – 3.29 (m, 2H), 2.18 – 1.70 (m, 4H), 1.57 (s, 18H). The analytical data is consistent that reported in the literature[4].

Diphenyl (1-amino-4-guanidinobutyl)phosphonate hydrobromide (30). Diphenyl *N*-benzyloxycarbonyl-amino-(3-(*N,N'*-bis(tert-butyloxycarbonyl)guanyl)propyl)-methanephosphonate (510 mg, 0.73 mmol, 1 eq.) was dissolved in 33% HBr in AcOH (3.6 mL)The reaction was stirred at RT for 2 h and the solvent was removed under reduced pressure. The residue was washed with cold Et_2O and dried under high vacuum to afford **30** (300 mg, 0.68 mmol, 93%) as a yellow powder. ^{1}H NMR (400 MHz, MeOD) δ 7.59 – 7.16 (m, 10H), 4.25 (dt, J = 14.2, 7.0, 1H), 3.38 – 3.30 (m, 2H), 2.36 – 2.05 (m, 2H), 2.03 – 1.87 (m, 2H). 13C NMR (101 MHz, Methanol-d_4) δ 129.9, 129.8, 126.0, 120.4, 120.3, 120.3, 40.4, 25.5, 25.2. (HRMS (ESI *m/z*) calculated for $C_{17}H_{24}N_4O_3P$ $[M+H]^+$ 363.1581, found 363.1580.

Synthesis of H_2N-Phg(4-guan)PP

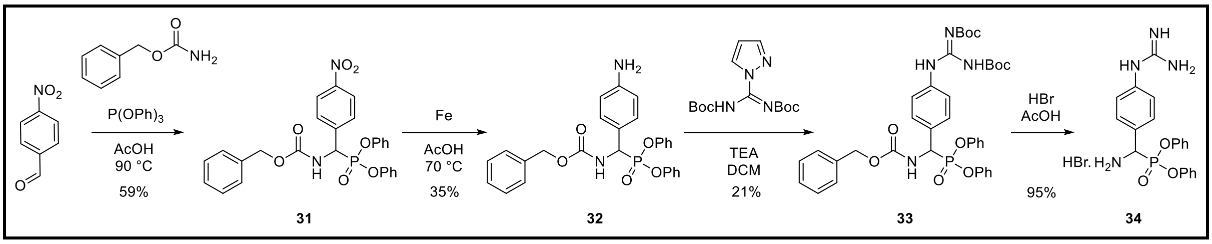

Benzyl ((diphenoxyphosphoryl)(4-nitrophenyl)methyl)carbamate 31. Benzyl carbamate (3.02 g, 20 mmol, 1 eq.), 4-nitrobenzayldehyde (3.02 g, 20 mmol, 1 eq.) and triphenyl phosphite (5.2 mL, 20 mmol, 1 eq.) were dissolved in acetic acid (35 mL) and stirred at 90 °C for 2 h. The solvent was removed under reduced pressure and a minimum amount of MeOH was added to dissolve the residue.

The mixture was stored at -20 °C for 16 h to allow the product to precipitate. The product was filtered, washed with cold MeOH and cold Et_2O to yield **31** (6.1g, 11.8 mmol, 59%) as a white powder. ^{1}H NMR (400 MHz, DMSO-d6) δ 9.13 (d, *J* = 9.9, 1H), 8.29 (d, *J* = 8.4, 2H), 7.96 (d, *J* = 8.1, 2H), 7.50 – 7.27 (m, 9H), 7.22 (dt, *J* = 10.3, 5.2, 2H), 7.06 (dd, *J* = 13.0, 8.1, 4H), 5.89 (dd, *J* = 23.7, 10.1, 1H), 5.17 (d, *J* = 12.5, 1H), 5.08 (d, *J* = 12.4, 1H). The analytical data is consistent that reported in the literature[4].

Benzyl ((4-aminophenyl)(diphenoxyphosphoryl)methyl)carbamate 32. Benzyl ((diphenoxyphosphoryl)-(4-nitrophenyl)methyl)carbamate (6.1 g, 11.8 mmol, 1 eq.) and iron (5.95 g, 106.2 mmol, 9 eq.) were dissolved in acetic acid (46 mL). The mixture was stirred at 70 °C for 2 h. The solvent was removed under reduced pressure and the crude solid was dissolved in EtOAc. The mixture was centrifuged at 4000 rpm for 5 min and the supernatant was separated. The supernatant was concentrated under reduced pressure to yield **32** (4 g) as a brown powder. The crude product was used without further purification.

Benzyl((diphenoxyphosphoryl)(4-(*N*, *N'* di-Boc)guanidinophenyl)methyl)carbamate 33. To a solution of *N*,*N'*-Di-Boc-1H-pyrazole-1-carboxamidine (2.5 g, 8.4 mmol, 1 eq.) and Benzyl ((4-aminophenyl)-(diphenoxyphosphoryl)methyl)carbamate (4 g, 8.4 mmol, 1 eq.) in DCM (20 mL) was added triethylamine (2.3 mL, 16.4 mmol, 2 eq.). The reaction was stirred at RT for 16 h. The solvent was removed under reduced pressure and the residue was redissolved in EtOAc. The solution was washed with aqueous 1N HCl, aqueous saturated $NaHCO_3$ and brine. The organic layer was dried with $MgSO_4$, filtered and concentrated under reduced pressure. Purification by flash column chromatography yielded the **33** (1.3 mg, 1.8 mmol, 21%) as a white powder. ^{1}H NMR (400 MHz, DMSO-d6) δ 11.41 (s, 1H), 10.03 (s, 1H), 8.90 (d, *J* = 10.1, 1H), 7.65 – 7.54 (m, 4H), 7.41 – 7.30 (m, 9H), 7.21 (t, *J* = 7.4, 2H), 7.06 (d, *J* = 8.1, 2H), 6.99 (d, *J* = 8.2, 2H), 5.60 (dd, *J* = 22.2, 10.1, 1H), 5.15 (d, *J* = 12.5, 1H), 5.07 (d, *J* = 12.5, 1H), 1.52 (s, 9H), 1.41 (s, 9H). The analytical data is consistent that reported in the literature[4].

Diphenyl (amino(4-guanidinophenyl)methyl)phosphonate hydrobromide 34. Benzyl((diphenoxyphosphoryl)(4-(N, N' di-Boc)guanidinophenyl)methyl)carbamate (1.3 g, 1.8 mmol, 1 eq.) was dissolved in 4 mL of a solution of HBr (33% in acetic acid). The reaction was stirred at RT for 2 h and the solvent was removed under reduced pressure. The residue was dissolved in a minimum amount of MeOH and excess Et_2O was added, followed by storage at -20 °C for 16 h. The precipitate was filtered, wash with cold Et_2O and dried under high vacuum to yield **34** (820 mg, 1.72 mmol, 95%) as a yellow powder. ^{1}H NMR (400 MHz, DMSO-d6) δ 9.90 (s, 1H), 9.47 (s, 3H), 7.74 (d, *J* = 8.1, 2H), 7.57 (s, 4H), 7.47 – 7.32 (m, 5H), 7.26 (m, 2H), 7.15 (d, *J* = 8.0, 2H), 7.03 (d, *J* = 8.0, 2H), 5.74 (d, *J* = 18.4, 1H). The analytical data is consistent that reported in the literature[4].

Biological experiments

Substrate screening assays. All screening assays were performed in triplicates. For the 20 natural amino acids, the triplicates were performed on the same 96 well plate. For the 81 unnatural amino acids, the triplicates were performed on three separate plates. As a control, the top hit from the natural amino acid screen was added as part of the unnatural amino acid screen, thereby allowing all the results to be presented in the same bar chart. 25 µL of assay buffer was added into each well, followed by 1 µL of fluorogenic peptide (10 mM stock). The reaction was initiated via the addition of 25 µL of enzyme to a final concentration of 10 nM for KLK6 and 1.5 nM for KLK8. At room temperature, fluorescence was read once every 30 s for 30 min from an Envision plate reader at excitation/emission wavelengths of 380nm/440nm. The data was analysed using Graphpad Prism 8. The initial slopes of each plot were calculated and presented in a bar chart, which represents the substrate specificity of the KLK. Error bars represent the standard deviation between the three replicates.

Enzyme kinetics to calculate K_M. Purified fluorogenic substrates were measured in triplicates. 72 µL of assay buffer was added into the top well of a 96 well plate, followed by 3 µL of substrate to a final substrate concentration of 1 mM and a DMSO concentration of 4%. 25 µL of assay buffer (with 4% DMSO) was added to all of the wells below. The substrate was serially diluted downwards by pipetting 50 µL. Thus, a concentration range from 1mM to 58 µM was established. The reaction was initiated via the addition of enzyme (25 µL) to a final concentration of 1.5 nM for KLK6, 34 nM for KLK7, 1 nM for KLK8, 2 nM for tPA and uPA. The final substrate concentration range was therefore 500 µM to 29 µM with 2% DMSO. Less soluble substrates had a concentration range of 250 µM to 15 µM. At room temperature, fluorescence was read once every 30 s for 30 min from an Envision plate reader at excitation/emission wavelengths of 380nm/440nm. The data was analysed with Graphpad Prism 8, using the Michaelis-Menten model.

Enzyme kinetics to calculate k_{obs}/I. All ABPs and inhibitors were measured in triplicates. A 2x stock of fluorogenic substrate was prepared in assay buffer to a final DMSO concentration of 1%. The fluorogenic substrates and final assay concentrations were: **22** (5 µM) for KLK6, **24** (5 µM) for KLK7, Boc-VPR-AMC (30 µM) for KLK8 and Z-GGR-AMC (60 µM) for uPA and tPA. 73.5 µL of this buffer was added to the leftmost wells of a 96 plate, followed by the addition of the ABP or inhibitor (1.5 µL). Final concentrations of ABP or inhibitor depends on its potency. DMSO was added into the buffer stock to match the concentration of the leftmost wells. 25 µL of this modified buffer was added into the rest of the wells in the same row. The test compound was serially diluted rightwards by pipetting 50 µL. A DMSO control was also included. The assay was initiated by the addition of enzyme (25 µL) to a final concentration of 1.5 nM for KLK6, 34 nM for KLK7, 1 nM for KLK8, 2 nM for uPA and tPA. At room temperature, fluorescence was read once every 30 s for 30 min from an Envision plate reader at excitation/emission wavelengths of 380nm/440nm. The data was analysed with Graphpad Prism 8, using a pseudo first order kinetics model. The observed rate of inhibition (k_{obs}) was calculated by plotting a one-phase association. The k_{obs} value was plotted against inhibitor concentration. The slope of this plot was multiplied by a substrate competition factor (1 + [S]/K_M), where [S] is the substrate

concentration used and K_M is the concentration of substrate required to reach half of the maximum rate. This gives the final k_{obs}/I value.

Cell culture. Cells were cultured in their recommended culture media supplemented with 10% (v/v) foetal bovine serum at 37 °C and 5% CO_2.

Preparation of conditioned media. Cells were grown in standard growth media to 80% confluency in a T175 flask. The medium was removed, and the cells were washed with serum-free, phenol-red free RPMI. The cells were incubated with the same RPMI for 2 h. The medium was replaced once again with serum-free, phenol-red free RPMI and the cells were incubated for 48 h. The condition medium was collected and filtered through a 0.2 µm syringe filter. The medium was concentrated (25 mL to 0.75 mL) using a 3 kDa Mw cutoff spin filter to a final concentration of around 1 mg/mL. The protein concentration was measured using a BioRad DC™ protein assay.

Pulldown experiments (western blot). Conditioned media was diluted with PBS to exactly 1 mg/mL. 200 µg of total protein was used per condition. The ABP or DMSO was added at a 1:100 dilution and the samples were shaken at RT for 1 h. The proteins were precipitated using the MeOH/H_2O/$CHCl_3$ method. The pellet was washed twice with MeOH and dried in air for 5 min. The protein was resuspended in 2% SDS in PBS to a concentration of 10 mg/mL. PBS was added to gradually dilute the protein back to 1 mg/mL and the samples were centrifuged (17,000 g, 5 min). 5 µg of protein was set aside as the "before pulldown" samples. Magnetic streptavidin beads were washed three times with PBS. The rest of the protein was added into the beads and shaken at RT for 2 h. 5 µg of the supernatant was set aside for analysis, while the rest was discarded. The beads were washed three times with 1% SDS in PBS. The beads were suspended in 1 × Laemmli loading buffer and boiled at 95 °C for 10 min. Loading buffer was also added to the "before pulldown" and "supernatant" samples and the samples were boiled at 95 °C for 5 min. The proteins were analysed via SDS-PAGE and western blot analysis.

SDS-PAGE. 10 well or 15 well 12% Bis-Tris polyacrylamide gels were hand casted using a National Diagnostics Protogel kit. The BioRad Precision Plus Protein™ All Blue ladder was used. A Tris/Glycine running buffer (7.2 mM Tris, 58.6 mM glycine, 0.06% SDS) was used. Samples were run at 80V for 10 min, followed by 180 V for 60 min. Gels were then taken for western blot or in-gel fluorescence.

Western Blot. The proteins were transferred from the gel into a nitrocellulose membrane at 100V for 60 min in transfer buffer (192 mM glycine, 25 mM tris, 20% MeOH in H_2O). The membrane was stained with Ponceau S to check for transfer efficiency. The membrane was washed with ultrapure water to remove the excess Ponceau S. A photo of the membrane was taken as a loading control. The membrane was blocked with BSA (5% in TBST) at room temperature for 1 h or at 4 °C overnight. The membrane was incubated with NeutrAvidin-HRP (Thermo 31030) or the primary antibody for KLK6 (Abcam ab190924), FGFBP1 (Abcam ab215353) or HSPG2 (BioTechne AF2364) in TBST at 4 °C overnight. Four TBST washes were performed (5 min each), followed by incubation with the secondary

antibody in TBST at room temperature for 1 h. The membrane was washed with TBST four times (5 min each). Chemiluminescence was imaged after the addition of the Immobilon-Crescendo HRP substrate.

Pulldown experiments (LC-MS/MS) Conditioned media was diluted with PBS to exactly 1 mg/mL. 300 µg of total protein was used per replicate. Each condition was performed in triplicate. The inhibitor or DMSO control (1:100 dilution) was incubated with the media at RT for 2 h with a gentle agitation. The ABP, inactive control or DMSO was added at a 1:100 dilution and the samples were shaken at RT for 1 h. The proteins were precipitated using the MeOH/H_2O/$CHCl_3$ method. The pellet was washed twice with MeOH and dried in air for 5 min. The proteins were resuspended in 2% SDS, 50 mM HEPES in H_2O (pH 8) to a concentration of 10 mg/mL. 50 mM HEPES was added to gradually dilute the protein back to 1 mg/mL and the samples were centrifuged (17,000 g, 5 min). A 1:1 mixture of NeutrAvidin agarose resin (15 µL per 300 µg protein) and control agarose resin (15 µL per 300 µg protein) was prepared and washed with 0.2% SDS, 50 mM HEPES. The test samples were added into the resin and the mixture was shaken at RT for 2 h. The supernatant was removed, and the beads were washed with 3 × 0.2% SDS, 50 mM HEPES and 3 × 50 mM HEPES. The beads were resuspended in 50 mM HEPES (50 µL). TCEP was added to a final concentration of 5 mM (1:20 dilution). Chloroacetamide was added to a final concentration of 10 mM (1:20 dilution). The mixture was shaken at RT for 30 min. To digest the peptides off the resin, 0.5 µL of trypsin (0.2 mg/mL stock) was added into each sample and the samples were shaken at 37 °C overnight. The supernatant was taken into a clean sample tube. 50 mM HEPES (50 µL) was added into the resin and the washings were combined with the original supernatant. Trifluoroacetic acid (0.8 µL) was added into the samples to deactivate the trypsin. The samples were centrifuged (17,000 g, 5 min) and desalted.

Desalting (stage-tipping). Stage tips were prepared using 3 layers of SDB-XC disks into 200 µL pipette tips. The stage tips were washed with MeOH (150 µL) and water (150 µL). The peptide samples were loaded into the stage tips, followed by a water wash (150 µL). The peptides were eluted with 60:40 MeCN:H_2O (60 µL) and the solvent was removed under reduced pressure at 45 °C using a Genevac EZ-2 evaporator. The peptides were resuspended in 0.5% TFA, 2% MeCN in H_2O (10 µL) and filtered through 3 layers of Durapore membrane. The peptides were stored at 4 °C until submission. Sample injection volume was between 1 µL to 3 µL.

LC-MS/MS methodology. Peptide samples prepared in the above procedure were run using a Thermofisher Q-Exactive LC-MS/MS equipped with a Thermo EASY-SPRAY column (500 mm x 75 µm inner diameter, 2 µm particle size). The gradient was for 70 min, from 5:95 MeCN:H_2O to 35:65 MeCN:H_2O with 0.1% formic acid. The flow rate was 250 nl/min. The chromatography was coupled to the Q-Exactive using an EASY-SPRAY source. The instrument was run at data-dependent mode and the resolution of the survey scans was 70,000 at m/z 200. Scans were acquired from 350 to 1650 m/z. The maximum ion injection time for the survey scan was 20 ms. The MS/MS scan was acquired at 35,000 at m/z 200. Up to the 10 most abundant isotopes with charge +2 from the survey scan were selected with a window of 2.0 m/z and fragmented by HCD with normalized collision energy of 31. The

maximum ion injection time for the MS/MS scan was 120 ms. The ion target value for MS was set to 10^6 and for MS/MS to 2×10^5. The intensity threshold was set to 8.3×10^2.

Sample preparation for N-terminomics. The N-terminal enrichment procedure was adapted from a reported protocol[5]. Capan-2 cells were grown to 80% confluency and washed with serum-free, phenol red-free RPMI (2 × 15 mL). The cells were incubated for 24 h (in 25 mL serum-free, phenol red-free RPMI) in the presence of the KLK6 ABP **17** (2 µM) or DMSO. The media was collected and filtered through a 0.2 µm syringe filter. The media was concentrated (25 mL to 0.75 mL) using a 3 kDa Mw cutoff spin filter to a final concentration of c.a. 1 mg/mL. The protein concentration was measured using a BioRad DC™ protein assay. The two conditions were split into three replicates for proteomics sample preparation. Each replicate contained 200 µg of total protein at 1 mg/mL. The samples were reduced and alkylated using TCEP and chloroacetamide. TCEP was added to a final concentration of 5 mM (1:20 dilution). Chloroacetamide was added to a final concentration of 10 mM (1:20 dilution). The samples were shaken for 45 min. The excess reagents were removed via MeOH/$CHCl_3$ precipitation. The pellet was washed with MeOH and dried in air for 5 min. The proteins were resuspended in HEPES (200 mM, pH 7) to 1 mg/mL. To ensure full solubilisation, the samples were shaken for 30 min and sonicated for 5 min. Formaldehyde was added to final concentration of 30 mM. Light formaldehyde was used for the DMSO treated samples, while heavy formaldehyde (CAS: 63101-50-8) was used for the ABP treated samples. Sodium cyanoborohydride was added to a final concentration of 15 mM. The samples were shaken for 1 h at 37 °C, followed by the addition of fresh formaldehyde and sodium cyanoborohydride. The samples were shaken for a further 3 h at 37 °C. To quench the reagents, tris buffer (4M, pH 6.8) was added to a final concentration of 500 mM. The samples were shaken at RT for 30 min. The proteins were precipitated again using MeOH/$CHCl_3$ and the pellet was washed with MeOH. The protein was resuspended in HEPES buffer (200 mM, pH 8), shaken for 30 min and sonicated for 5 min. 10 µL of trypsin (0.2 mg/mL stock) was added into each sample and the samples were shaken at 37 °C overnight. The heavy and light proteomes were combined and 5% of each sample was transferred into a fresh sample tube for whole proteome analysis and demethylation efficiency checks. Ethanol was added to a final percentage of 40%. To hydrophobically modify the newly generated N-termini, undecanal (10.8 µL) was added at a 20:1 w/w ratio of undecanal:total protein. Sodium cyanoborohydride was added to a final concentration of 30 mM. The pH was confirmed to be between pH 7 – 8 using pH paper and the samples were shaken for 1 h at 37 °C. The samples were acidified to pH 3 – 4 using TFA (4 µL). To remove the hydrophobically labelled peptides, C18 Sep-Pak columns (Waters WAT054960) were first preconditioned with MeOH (700 µL) and a solution of 0.1% TFA in 40% EtOH (700 µL). A nitrogen line was used to flow the mobile phase through the column. The peptide sample was filtered through the column and the filtrate was collected in a clean tube. The solvents were evaporated in a Speedvac at 45 °C overnight. The samples were resuspended in 0.5% TFA (100 µL). The N-terminally enriched samples and whole proteome samples were stage tipped as mentioned above.

Proteomic data analysis using label free quantification. The MS raw files were loaded into MaxQuant (Version 1.6.10.43). The spectral data was searched against the human FASTA file (UniProt, taxonomy 9606, accessed 3/12/2020). Trypsin/P was used as the digestion method, with three maximum missed cleavages. Cysteine carbamidomethylation was set as a fixed modification for all searches. For activity-based probe selectivity experiments, the probe adduct, along with methionine oxidation and N-terminal acetylation were set as variable modifications. For N-terminomics samples, heavy/light dimethylation, Gln/Glu to PyroGlu, methionine oxidation and N-terminal acetylation were set as variable modifications. Match between runs was switched on. LFQ intensity values or dimethylation efficiencies were processed using Perseus.

Proteomic data analysis using dimethyl labelling. The MS raw files were loaded into MaxQuant (Version 1.6.10.43). The spectral data was searched against the human FASTA file (UniProt, taxonomy 9606, accessed 3/12/2020). Trypsin/P was used as the digestion method, with three maximum missed cleavages. Heavy (34.06311 Da) or light (28.0313 Da) dimethylations (on N-terminus or Lys) were set as labels. Heavy/light dimethylation, Gln/Glu to PyroGlu, methionine oxidation and N-terminal acetylation were set as variable modifications. Requantify and match between runs were switched on.

Pulldown volcano plots using Perseus. The ProteinGroups text file generated from the MaxQuant search was imported to Perseus. The LFQ intensity values were chosen for quantification and the protein list was filtered against only ID by site, reverse and potential contaminants. The intensity values were transformed to $\log_2$. The mean values within each replicate (row) were subtracted, followed by the subtraction each condition (column) by the median. Volcano plots were made using a t-test to compare the DMSO conditions and the probe treated conditions ($n = 3$, FDR = 0.05, $s0 = 0.1$).

Dimethylation efficiency and % N-terminal peptide checks using Perseus. Evidence and Peptide text files generated from the MaxQuant search was imported into Perseus. The variable modifications were extracted from the evidence file and matched with the corresponding peptides in the peptide file using the peptide ID column. The dimethylation efficiency was calculated by the sum of the heavy and light lysine dimethylation counts divided by the total lysine count. The percentage of N-terminal peptides was also calculated by examining the percentage of peptides in the dataset that have starting positions 1 or 2.

HUNTER quantification using Perseus. The "ratio H/L normalised" values were extracted from the Peptide text file from the MaxQuant Search into Perseus. The rows were filtered against reverse and potential contaminants. Rows with two or more NaN's were filtered away. The ratio values were transformed to $\log_2$. The columns were subtracted by median, and rows were annotated as one condition. A one sample t-test was performed to isolate the significant hits ($p = 0.05$). The t-test positive hits that had $\log_2$ values lower than -0.5 or higher than 0.5 were kept. These hits were further filtered by searching for the P3 and P2 cleavage positions of each N-terminal peptide and comparing it with the substrate specificity data of KLK6 (**Figure S2**). For peptides with negative ratios, the P3 and P2

positions of the N-terminus was analysed. Hits with P2 or P3 amino acids that were not tolerated by KLK6 were filtered out. This analysis was not performed for peptides with positive ratios because the exact cleavage site of these hits were uncertain.

Expression and purification of KLK6. The full-length Kallikrein 6 protein with three point mutations (KLK6mut), with a 6xHis purification tag followed by TEV and enterokinase protease sites, inserted after the signal peptide, was expressed in mammalian Expi293F cells. In brief, the pXLG vector containing the KLK6 cDNA harbouring the following mutations (R78G, R80Q, N134Q) introduced to prevent proteolysis and N-linked glycosylation respectively[6], was transfected into 500 ml of Expi293F cells grown in suspension in FreeStyle 293F medium (ThermoFisher) using ExpiFectamine at 37°C, 8% CO_2 shaking at 125 rpm. The supernatant containing secreted mature KLK6mut protein was filtered and pH-adjusted with 25 ml Tris, pH 8.0 and NaCl was added to a final concentration of 100 mM. The supernatant was then passed over a 5 ml NiNTA Excel (Cytiva) column at 5 ml/min, washed with 10 CV running buffer (50 mM Tris-HCl, pH 8.0, 150 mM NaCl, 20 mM Imidazole) before elution with 250 mM imidazole in running buffer. The fractions containing eluted KLK6mut were pooled, 50 µl Enterokinase (800 U)(New England Biolabs, P8070S) was added and the cleavage mixture was dialysed overnight against the enterokinase buffer (50 mM Tris-HCl, pH 8.0, 150 mM NaCl, 2 mM $CaCl_2$). The following day, the cleaved KLK6mut protein was concentrated and applied to a Superdex75 (10/300) (Cytiva) size-exclusion column with a running buffer of 50 mM Tris-HCl, pH 8.0, 100 mM NaCl with a flow-rate of 0.5 ml/min. The KLK6mut protein eluted as a single peak and was concentrated to 8.5 mg/ml.

X-ray crystallography. Initial KLK6 crystals were obtained in a previously published condition with 0.1 M Tris-HCl pH 8.5, 18-24% PEG MME 2000, 0.20 M trimethylamine N-oxide and 5mM benzamidine[6]. Before crystallization, KLK6 at 10 mg/ml was incubated with 5 mM benzamidine. Crystals were set up with the SPT Labtech mosquito instrument in 96 well plates and were obtained by sitting drop vapour diffusion method by mixing 200 nL of protein and 200 nL mother liquor. Crystals appeared in 3-4 days at 20 °C and were then soaked in 1 mM of inhibitors for 2-3 days. The soaked crystals were cryoprotected in 0.1 M Tris-HCl pH 8.5, 18-24% PEG MME 2000, 0.20 M trimethylamine N-oxide, 30 % glycerol and the inhibitor and were snap frozen in liquid nitrogen. Multiple datasets were collected at the ID30A beamline at the European Synchrotron Radiation Facility (ESRF), Grenoble, France. Datasets were indexed and scaled with XDS[7] and the structures were solved by molecular replacement in Phaser[8] with the KLK6 structure published previously[6] (PDB ID: 3VFE) as the search model. Structures along with the inhibitor coordinates were built in the electron density with multiple rounds of model building and refinement in coot[9] and phenix.refine from the Phenix suite[10].

Incucyte (proliferation and toxicity) assays. Capan-2 cells were seeded in a sterile 96 well plate (10,000 cells per well). The outermost wells were left empty to mitigate edge effects. Cells were grown in growth media (10% FBS) overnight to adhere. The next day, the media was replaced with serum-free DMEM (117 µL) containing Sytox green (250 nM) or a DMSO control. The inhibitor or inactive control were serially diluted in a separate sterile 96 well plate as a 10x stock and were added into the cells

(13 µL per condition). Control conditions included puromycin (100 µg/mL), DMSO only, Sytox green only or DMSO + Sytox green. The final inhibitor concentrations ranged from 2 µM to 125 nM. The cells were placed into an Incucyte with the incubator set at 37 °C and 5% CO_2. Phase and green fluorescence were analysed every 4 h for 5 days.

Invasion/migration assays. Cell invasion was measured using Corning Fluoroblok inserts following a modified procedure[11]. Fluoroblok inserts were placed into the wells of a 24-well plate. Matrigel was suspended to 300 µg/mL in coating buffer (0.01M Tris, 0.7% NaCl, pH 8). The matrigel suspension (100 µL) was added into the Fluoroblok insert and the plate was left at 37 °C for 2 h. A migration control (with no matrigel) was also included. Cells were trypsinised and resuspended in serum-free DMEM. The leftover buffer in the chambers was decanted and 500 µL of cells (100,000 per well) were seeded into the apical chamber of the insert. DMEM with 10% FBS (750 µL) was added into the basal chamber as a chemoattractant. The plate was incubated at 37 °C and 5% CO_2 for 24 h. The chambers were removed and placed into a new 24-well plate containing 50 µM of CellTracker CMHC dye in HBSS. The cells were stained for 0.5 h and the chamber was removed and placed into the previous plate containing DMEM with 10% FBS. The cells were imaged for blue fluorescence (ex/em = 375/460) using a Celena S inverted fluorescence microscope. Percentage invasion was calculated by quantifying the cell area using ImageJ.

3. Compound Characterisation (Structure, HPLC and HRMS)

22 Ac-Thr(Z)-Ala-Phg-Arg-ACC

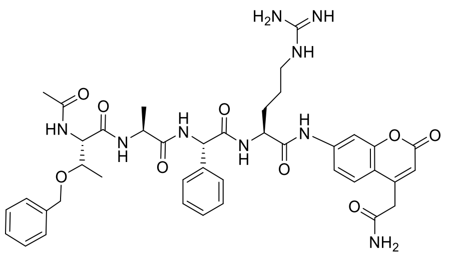

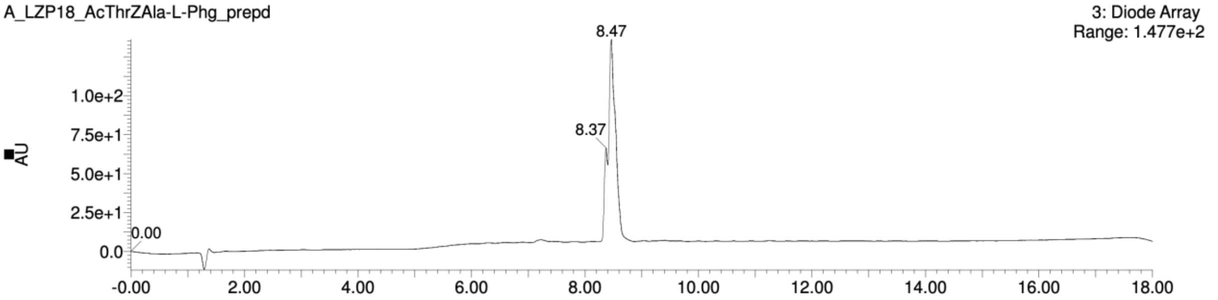

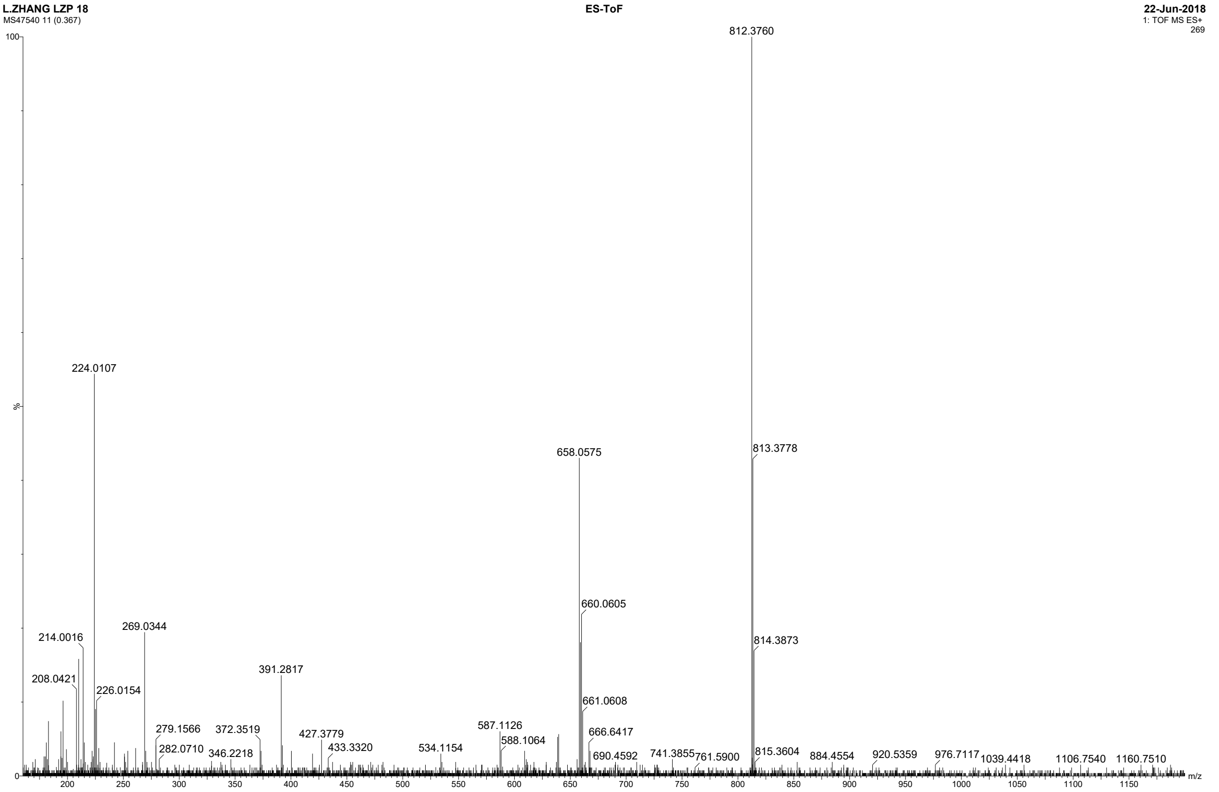

23 Ac-DPhe-Ser(Z)-Dht-Arg-ACC

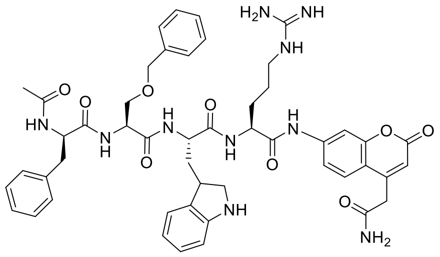

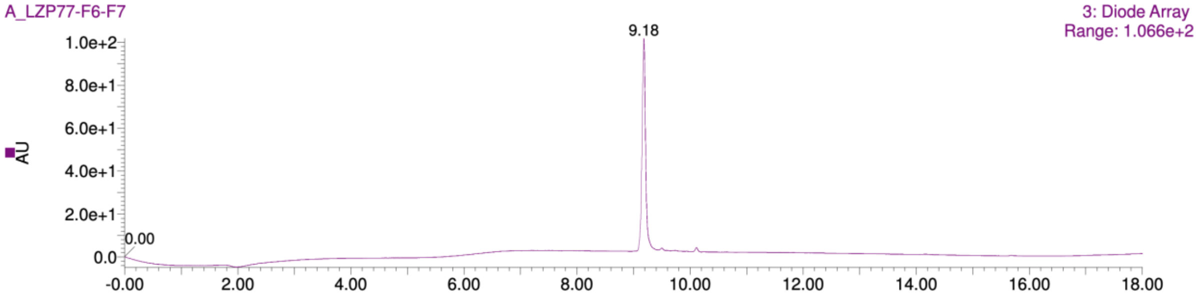

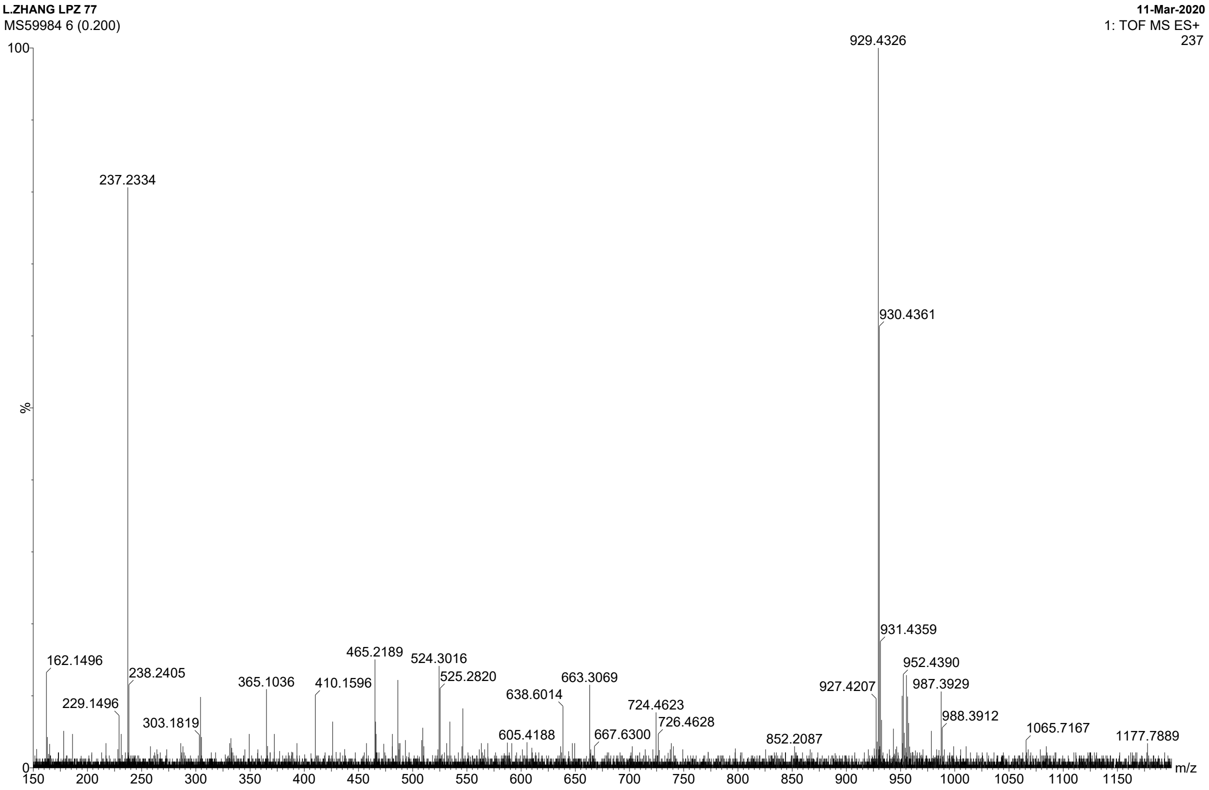

24 Ac-Lys-His-Leu-Phe-ACC

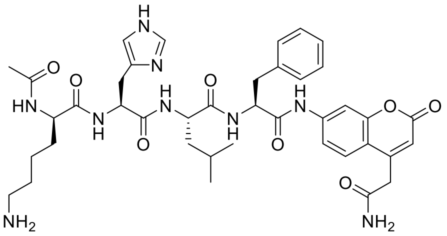

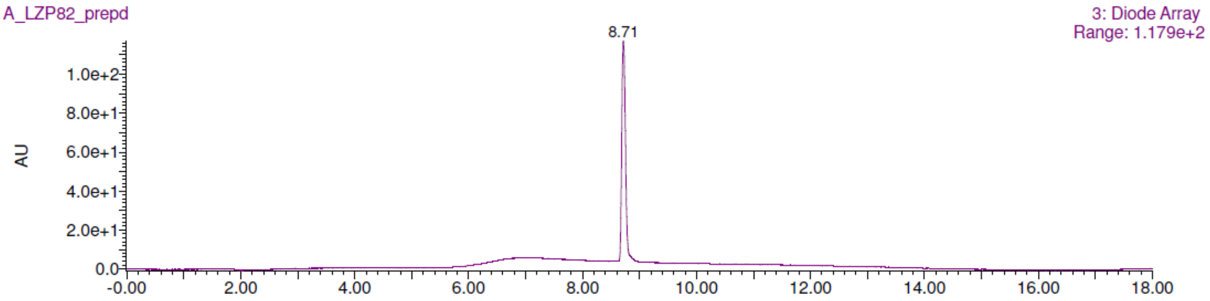

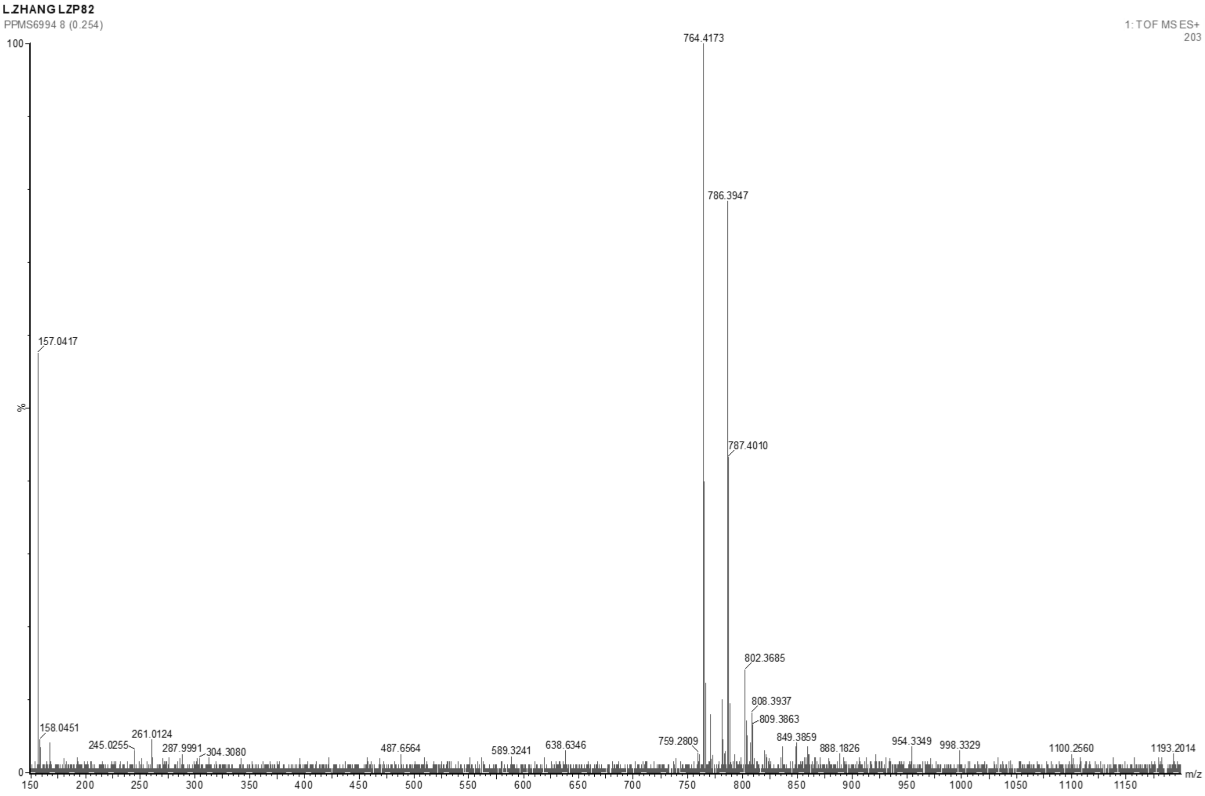

1 Yn-Thr(Z)-Ala-Phg-Phg(4-guan)-DPP

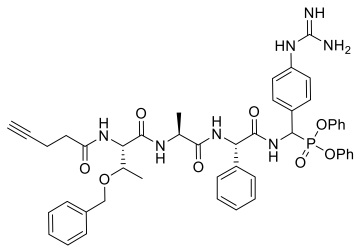

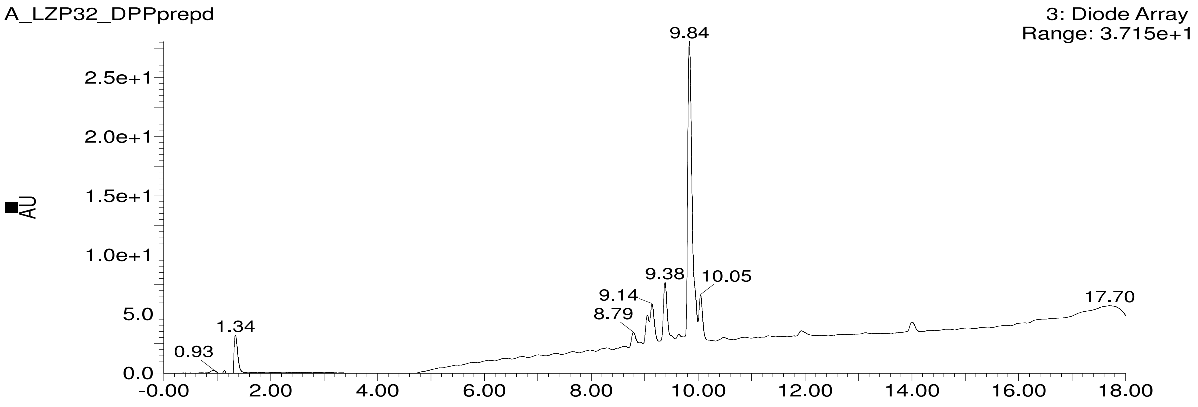

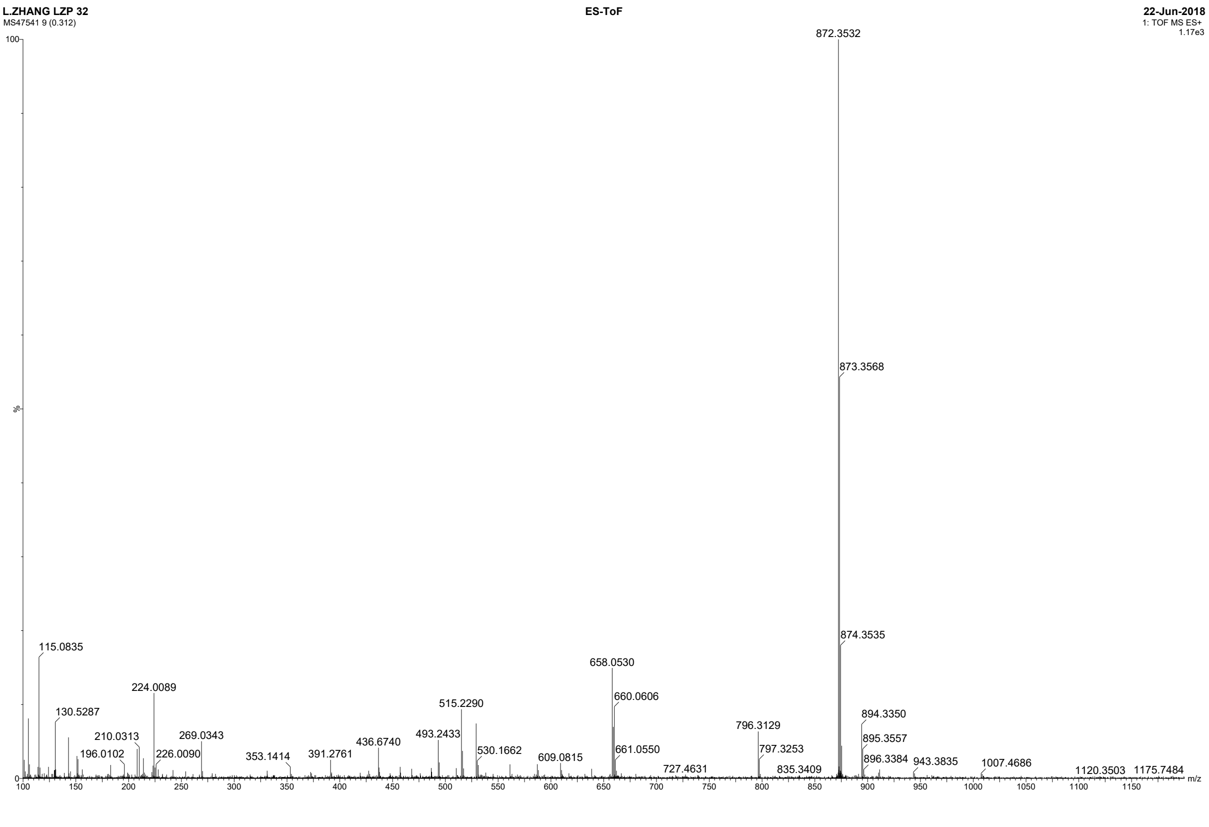

2 Yn-Thr(Z)-Chg-His(Z)-Phg(4-guan)-DPP

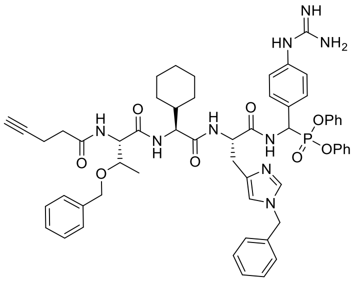

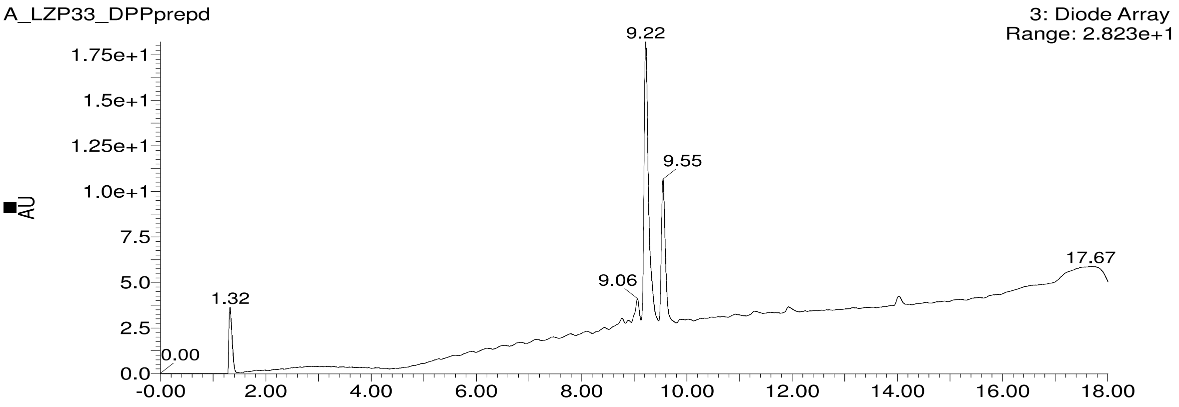

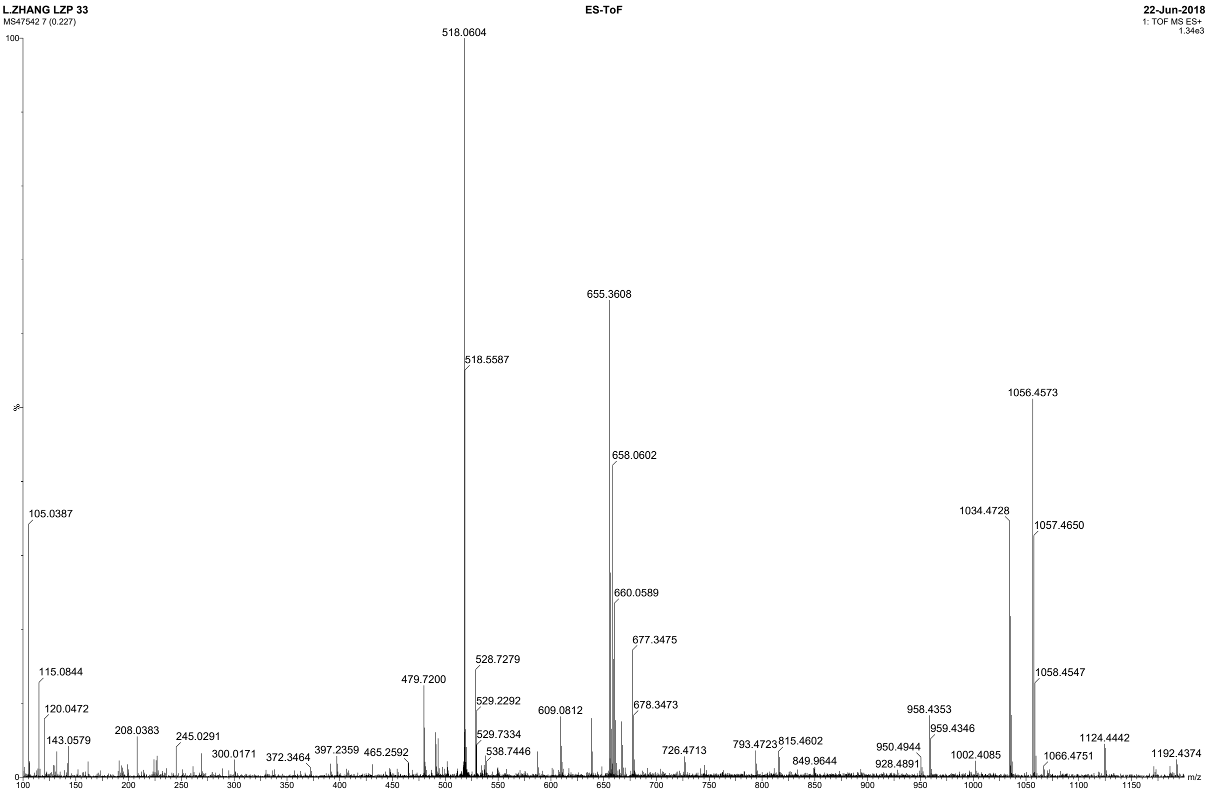

3 Yn-Thr(Z)-Cha-His(Z)-Phg(4-guan)-DPP

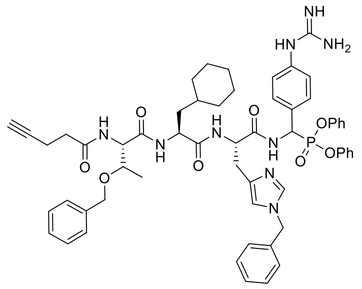

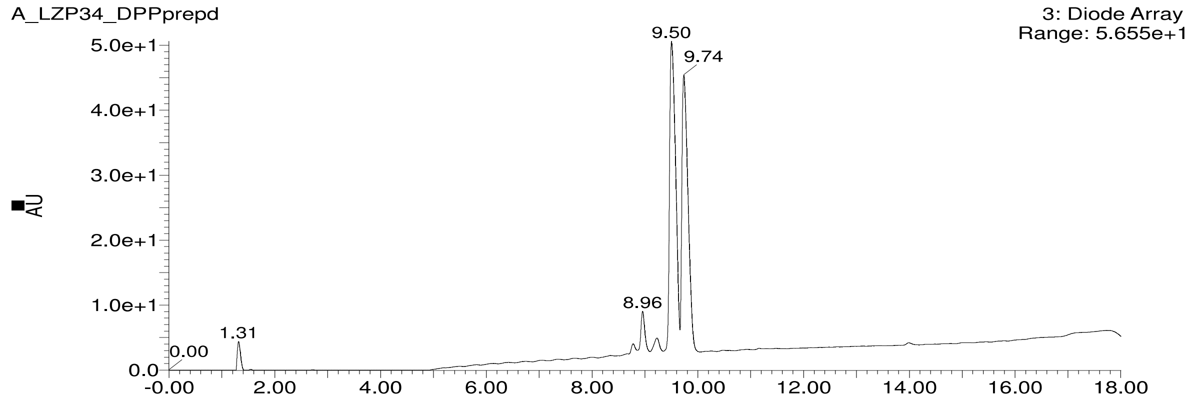

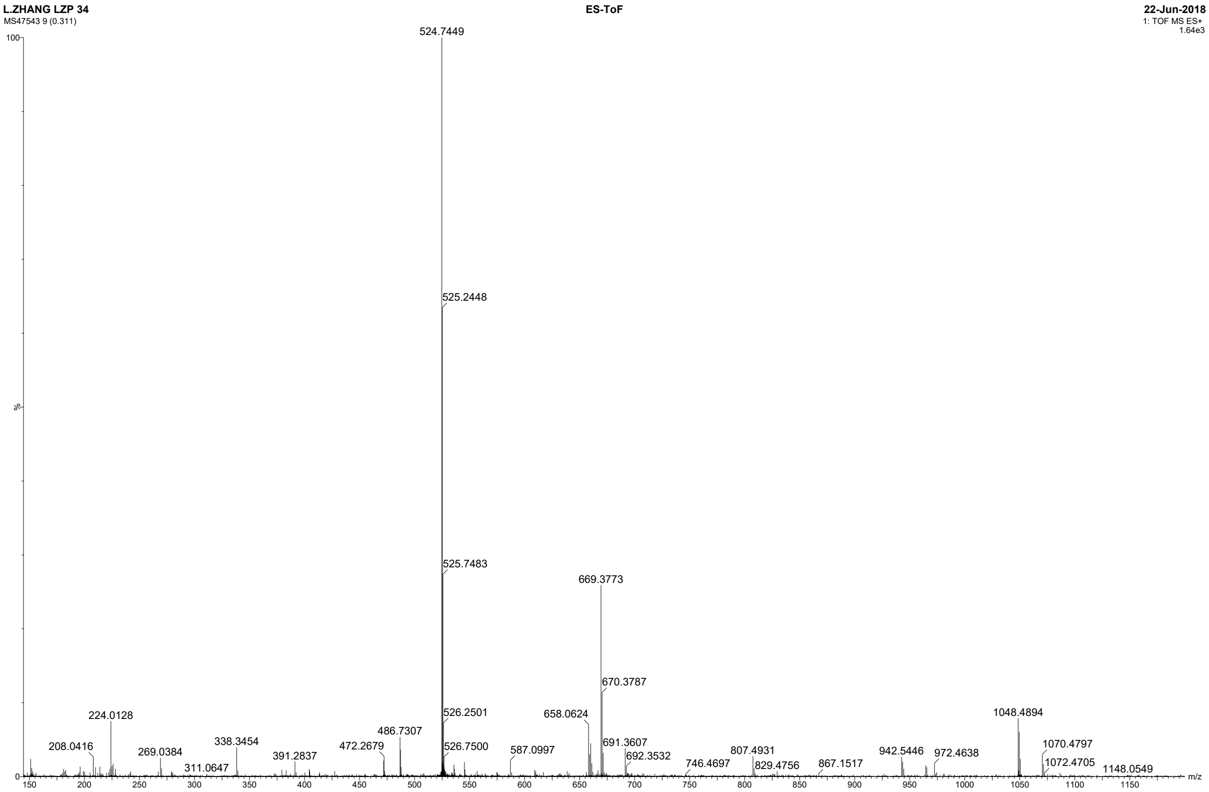

4 Yn-Asp(Chx)-Cha-His(Z)-Phg(4-guan)-DPP

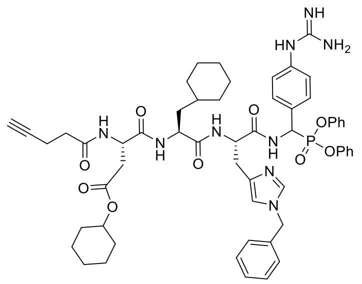

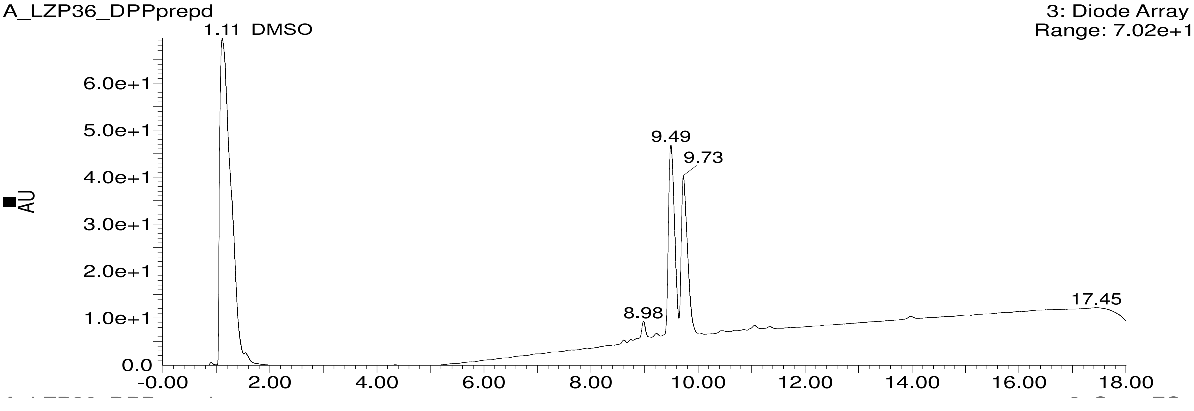

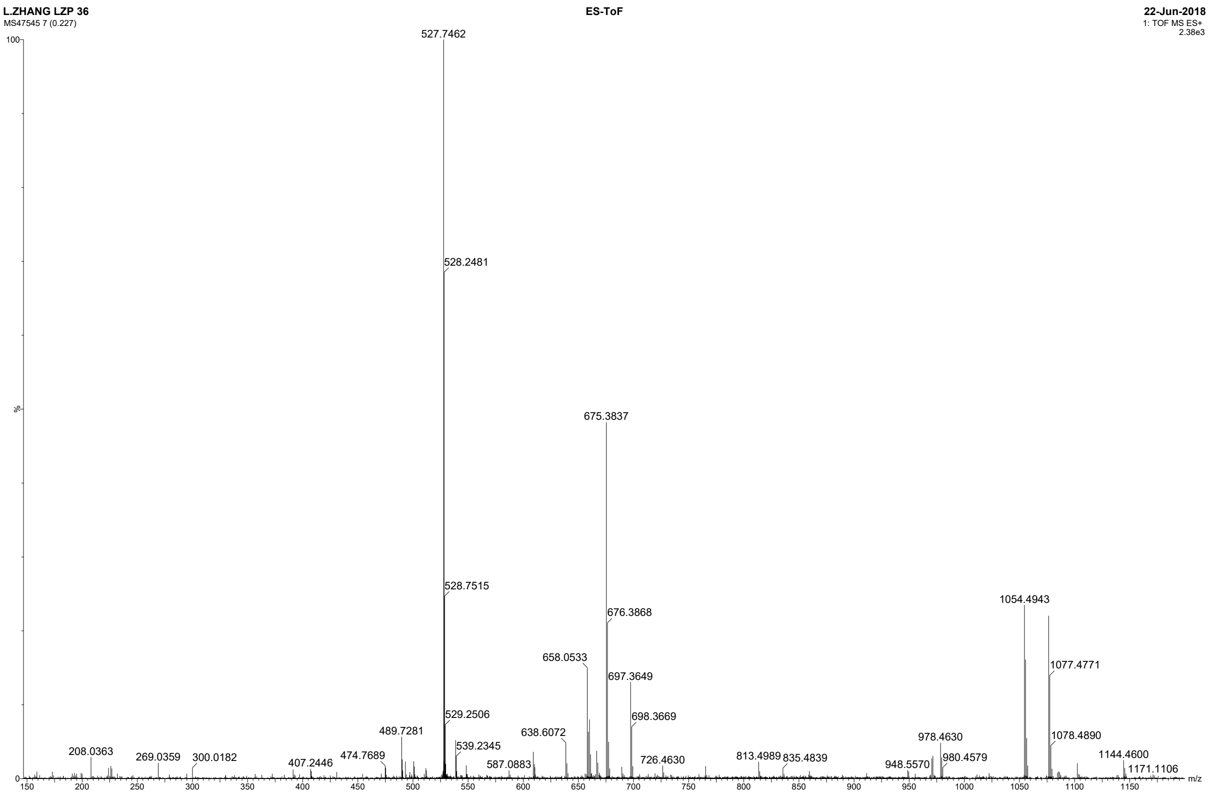

5 Yn-Thr(Z)-Ala-Dht-Phg(4-guan)-DPP

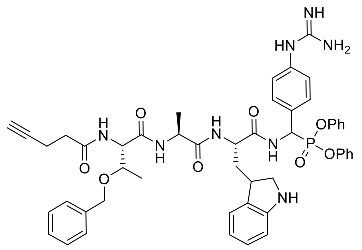

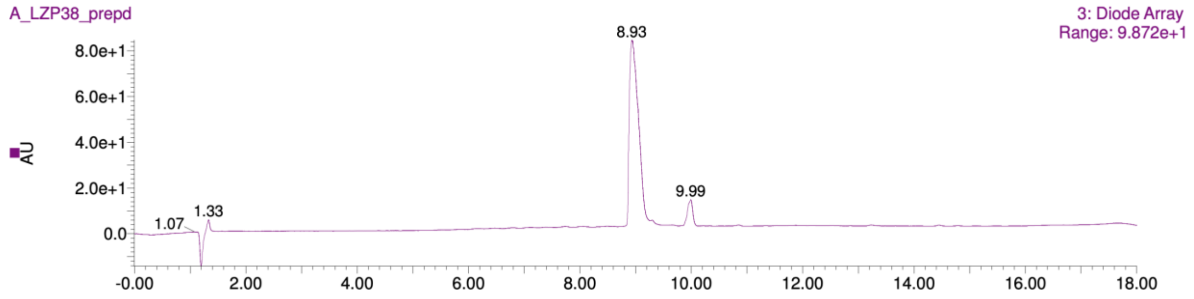

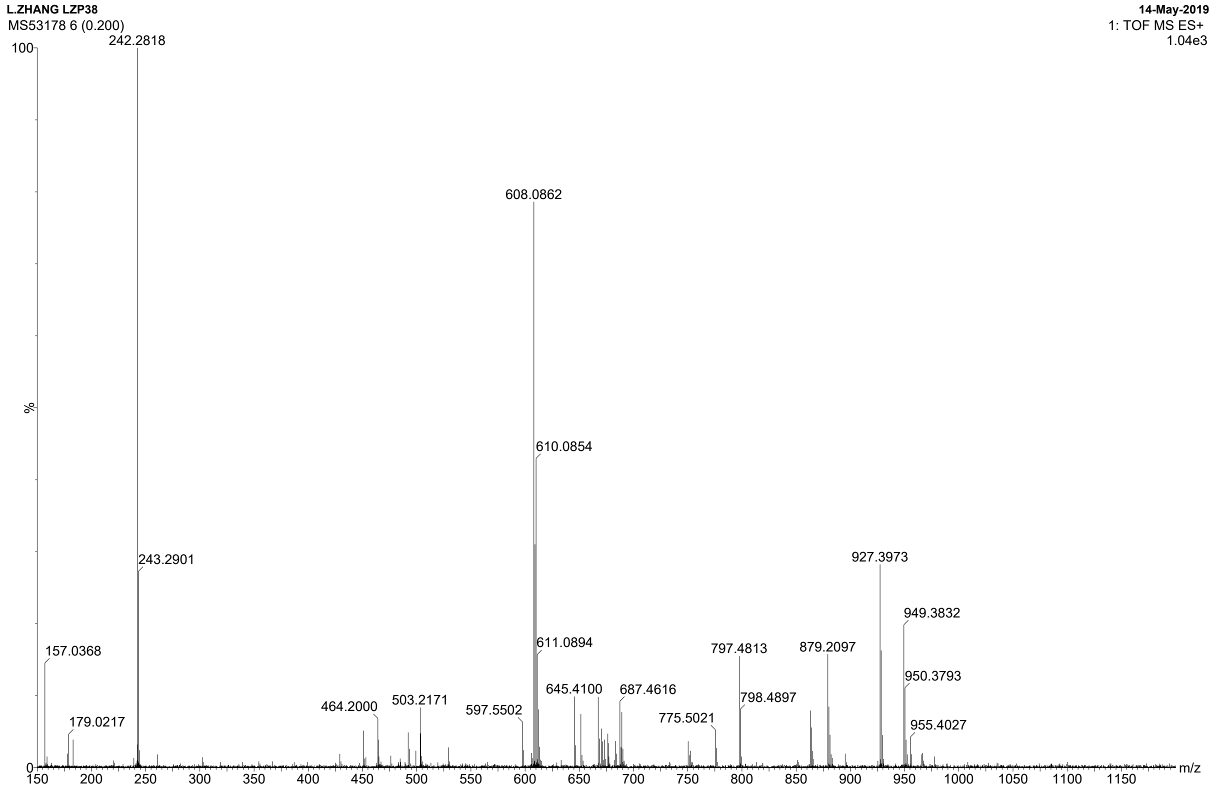

6 Yn-Thr(Z)-Ala-Tyr(Z)-Phg(4-guan)-DPP

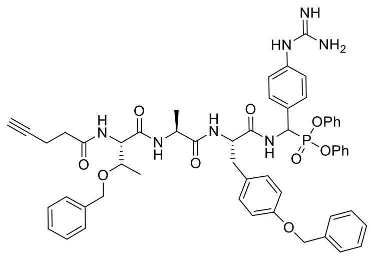

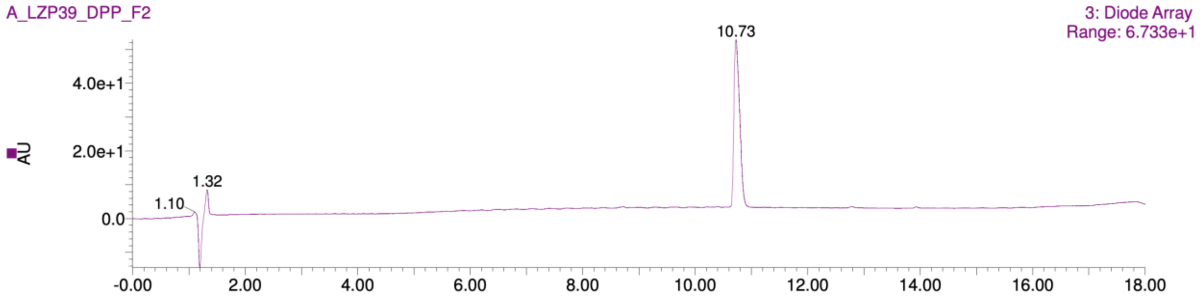

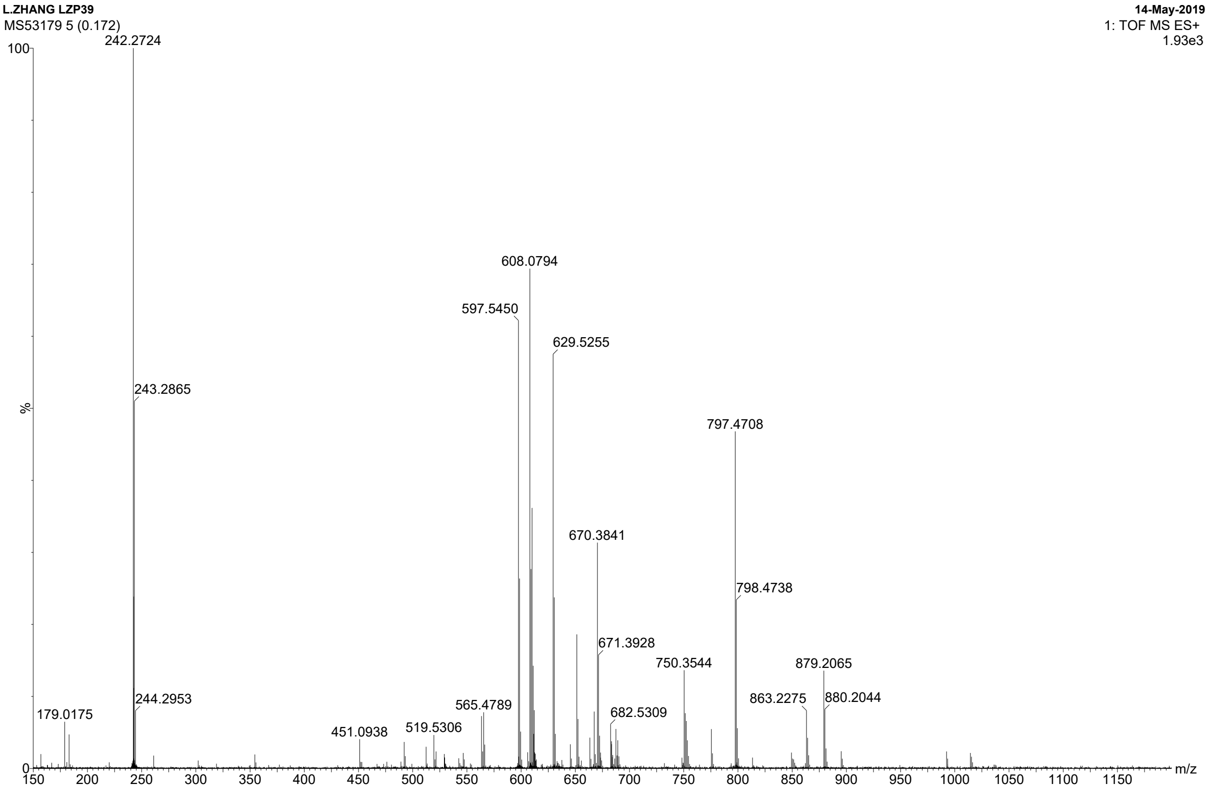

7 Yn-Thr(Z)-Ala-Ser(Z)-Phg(4-guan)-DPP

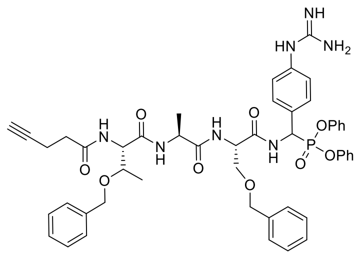

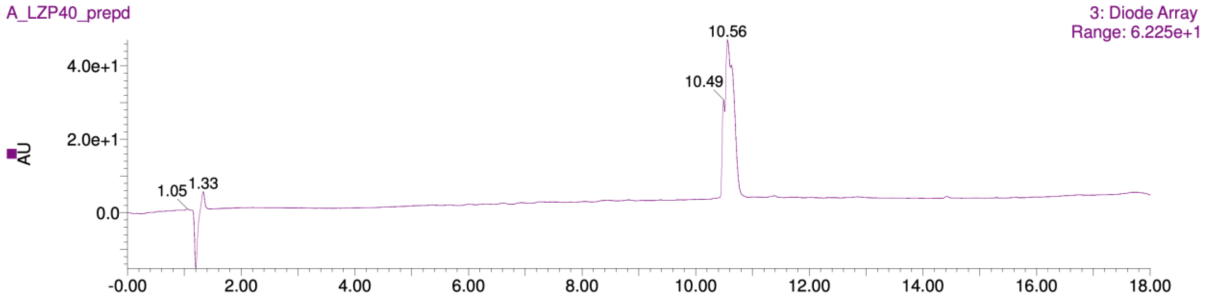

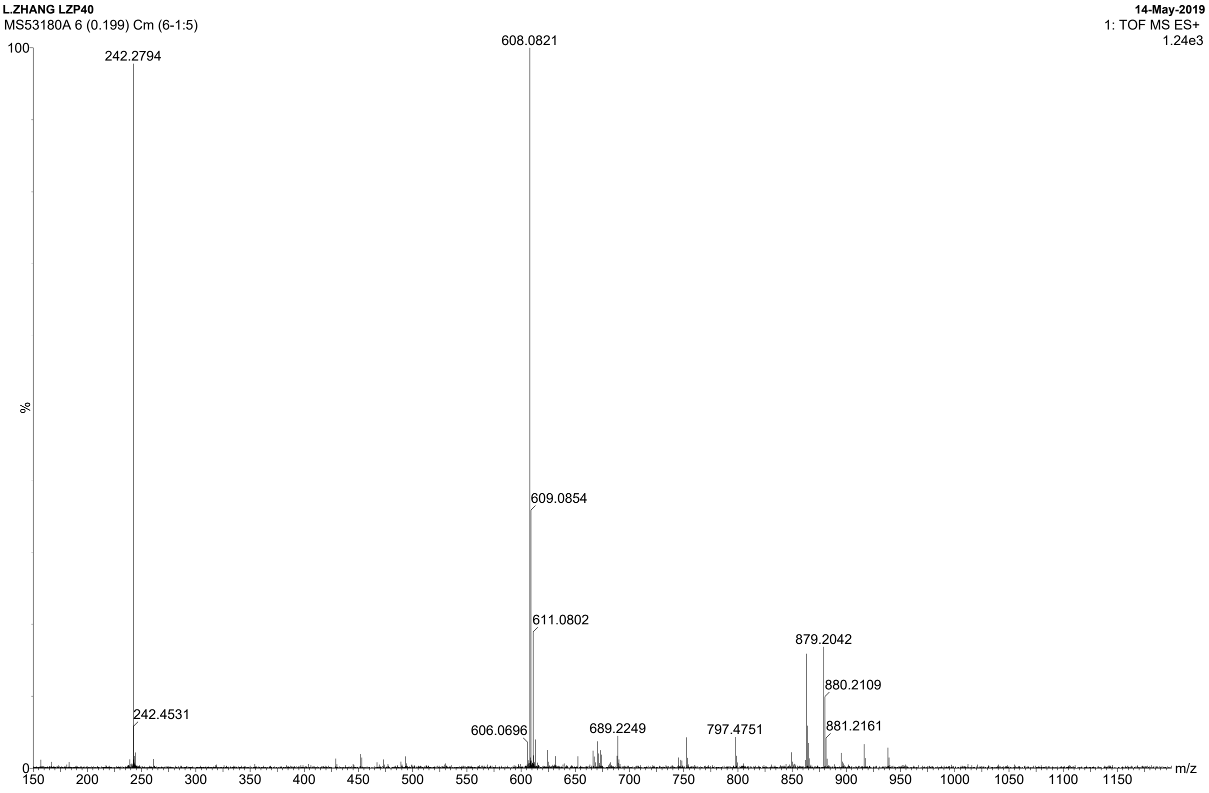

8 Yn-Thr(Z)-Ala-Dab(Z)-Phg(4-guan)-DPP

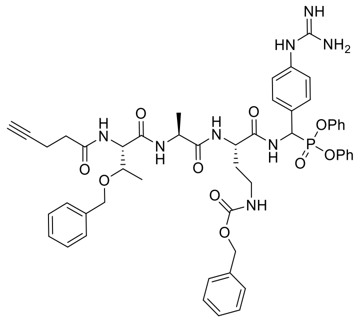

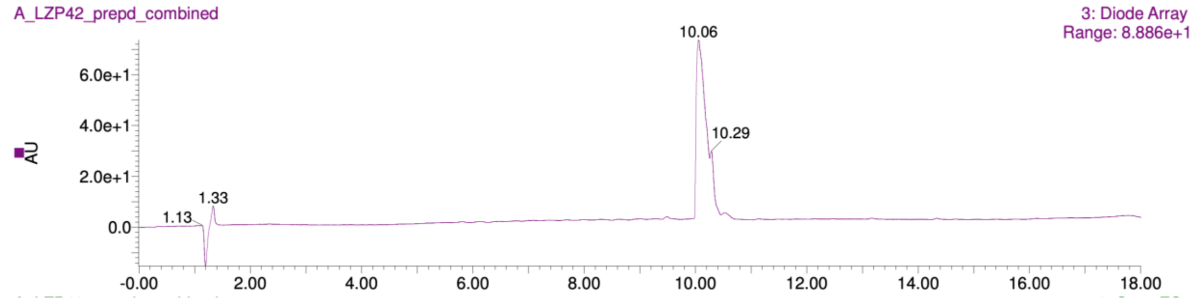

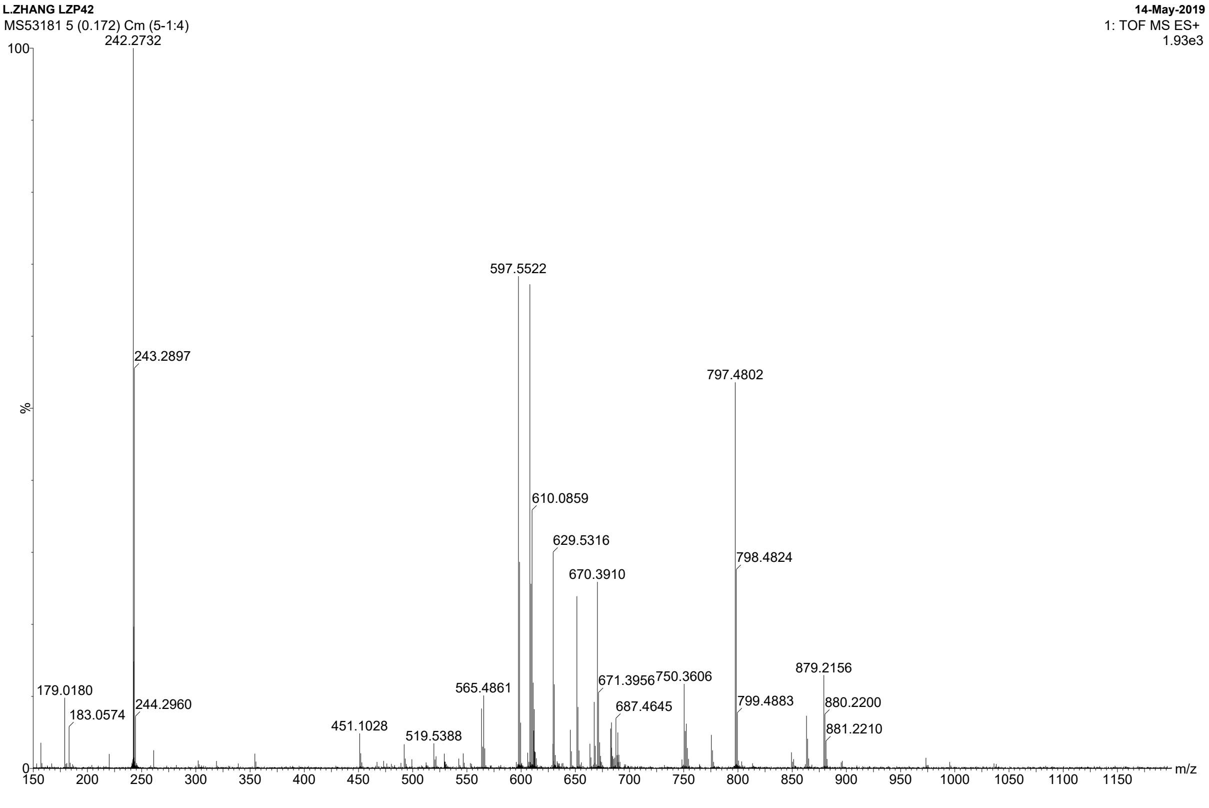

9 Biotin-Ahx-Thr(Z)-Ala-Dht-Phg(4-guan)-DPP

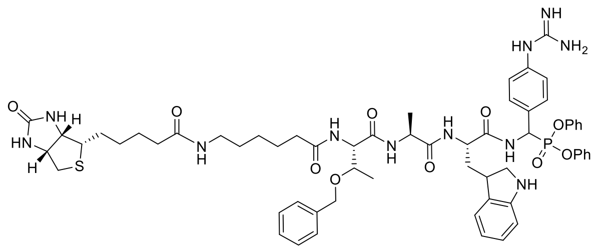

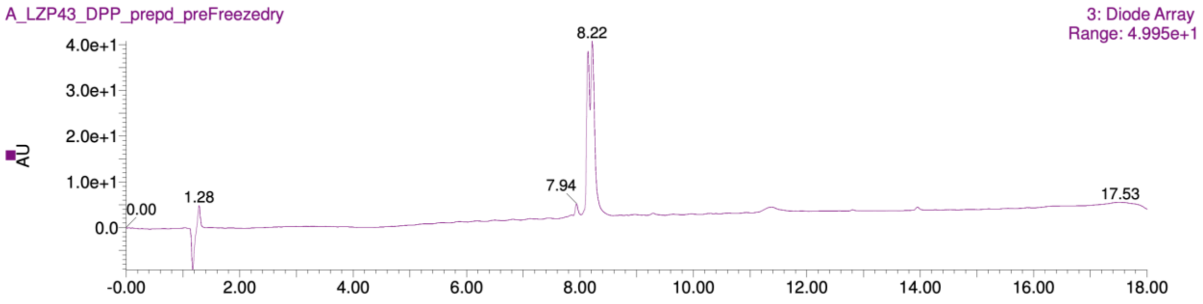

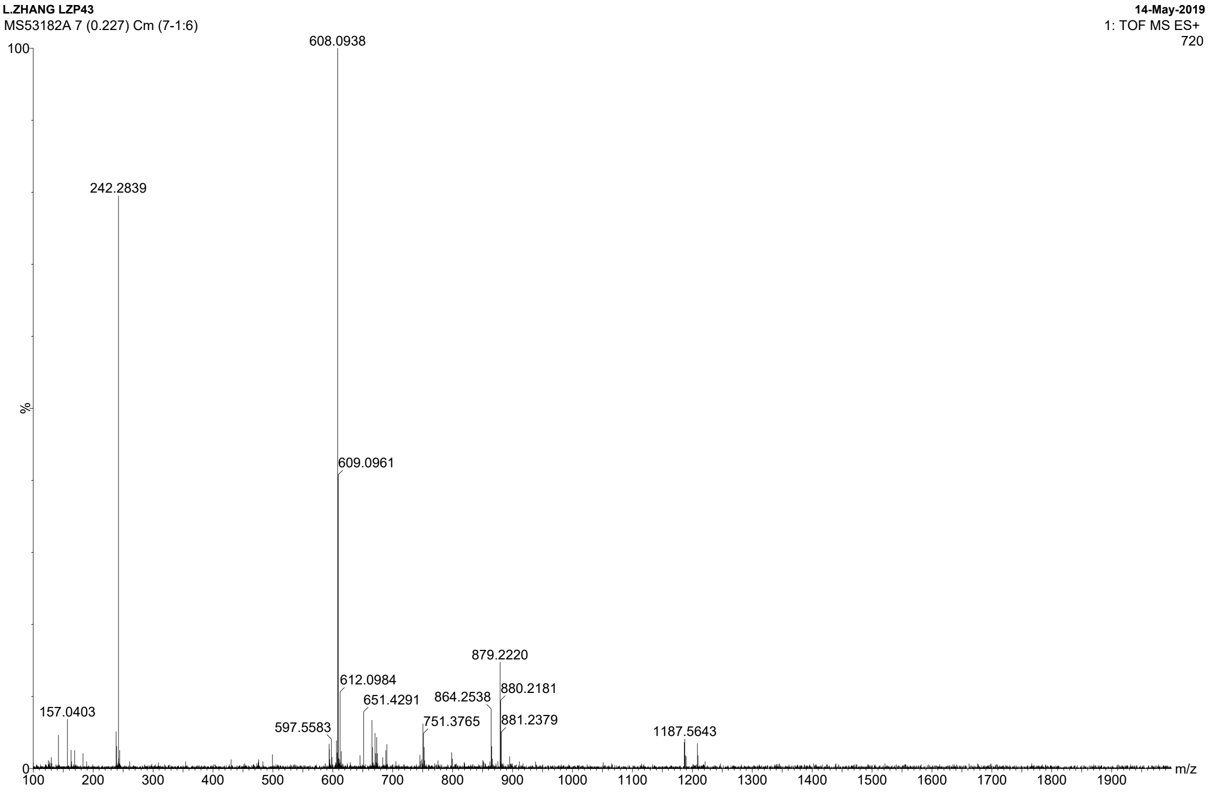

10 Biotin-Ahx-Thr(Z)-Cha-Dht-Phg(4-guan)-DPP

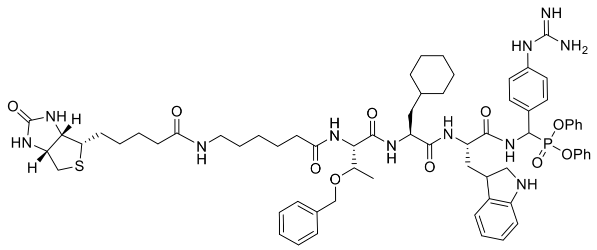

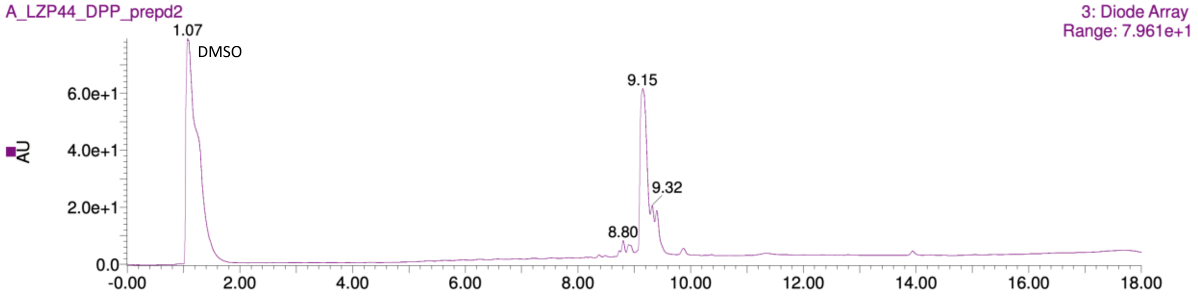

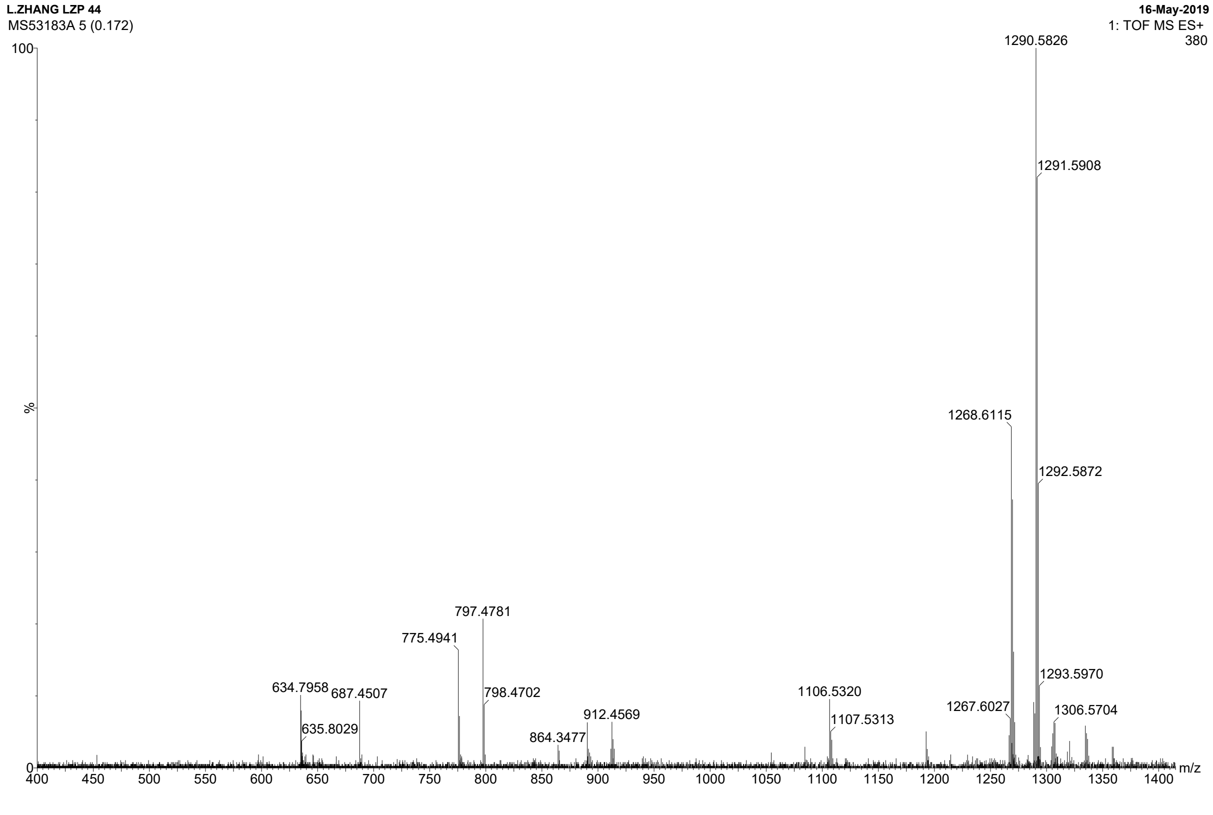

11 Biotin-Ahx-DPhe-Cha-Dht-Phg(4-guan)-DPP

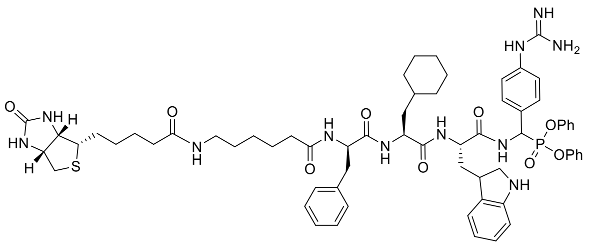

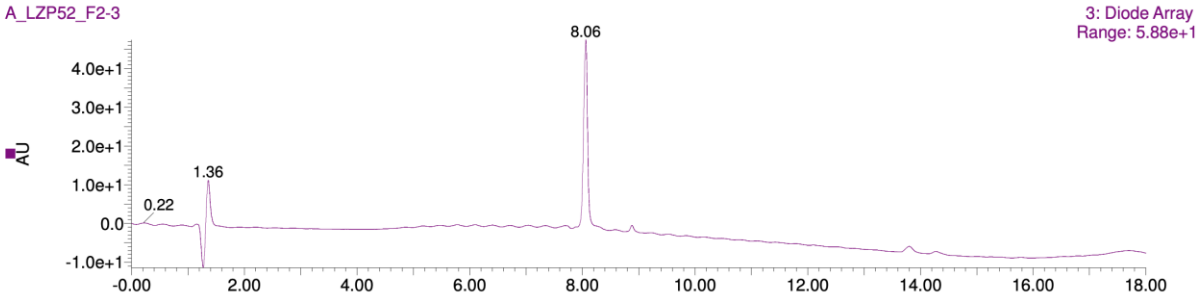

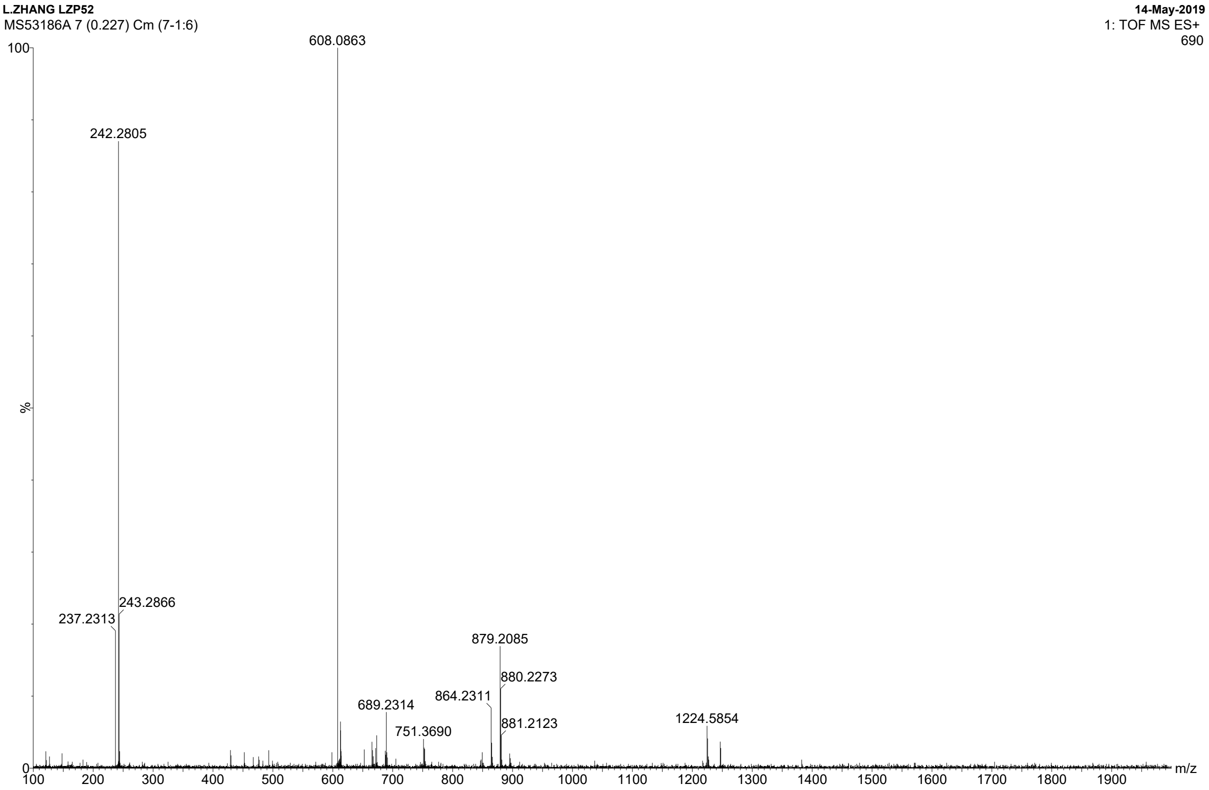

12 Biotin-Ahx-DhPhe-Cha-Dht-Phg(4-guan)-DPP

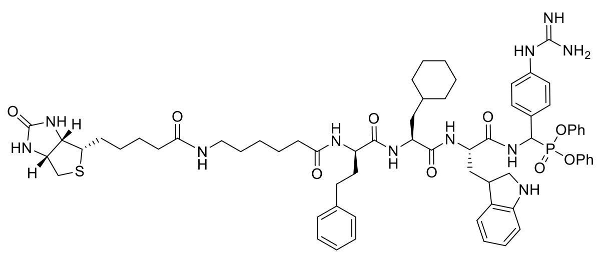

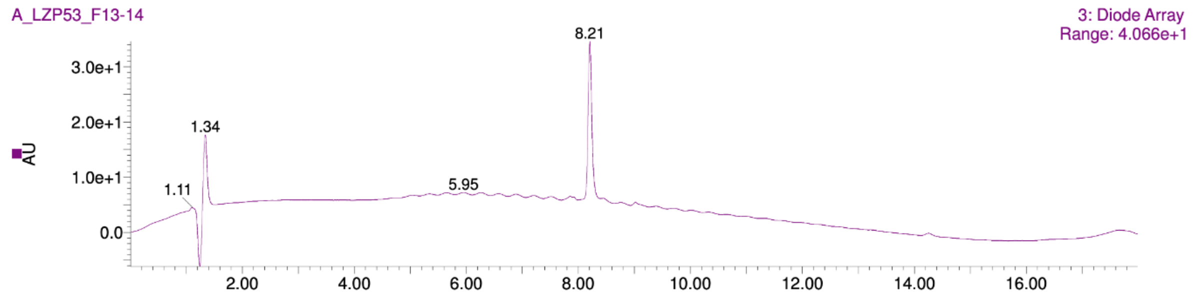

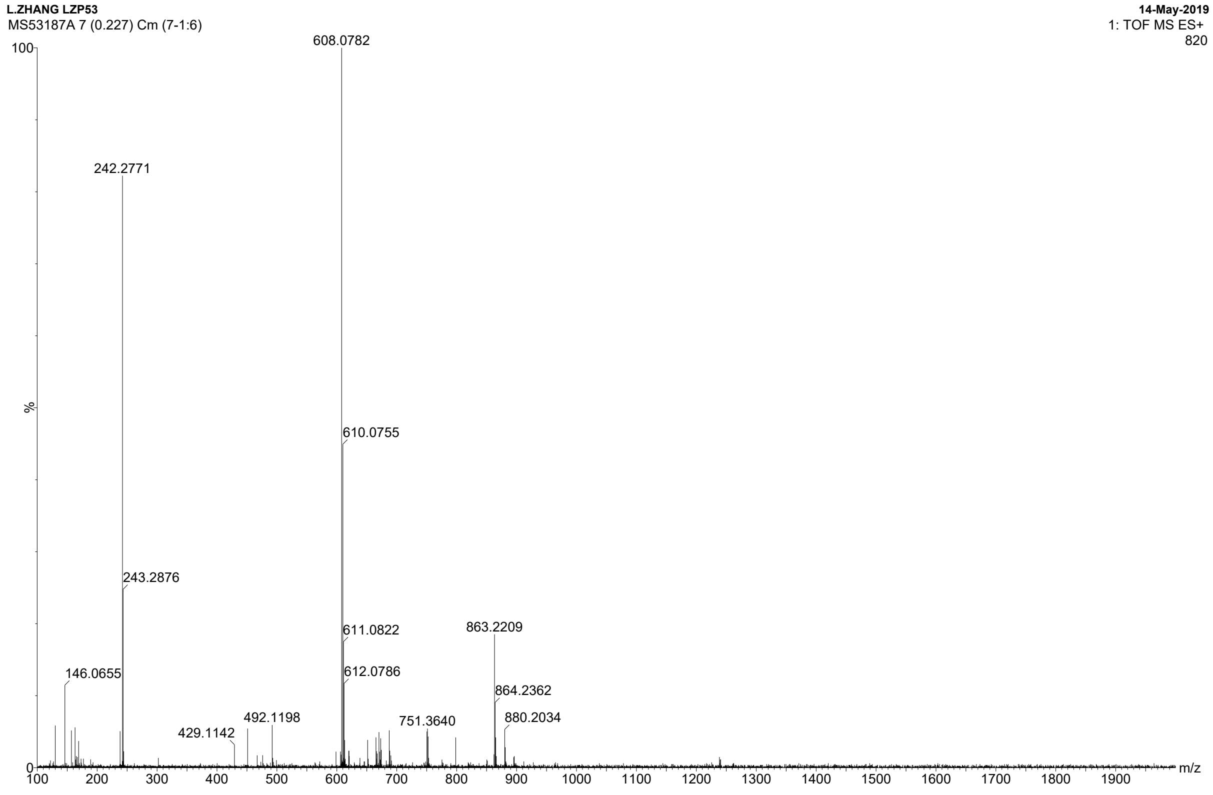

13 Biotin-Ahx-DPhe-Lys-Dht-Phg(4-guan)-DPP

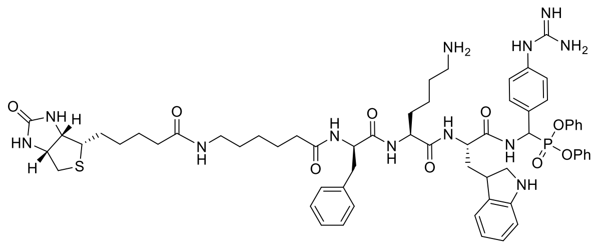

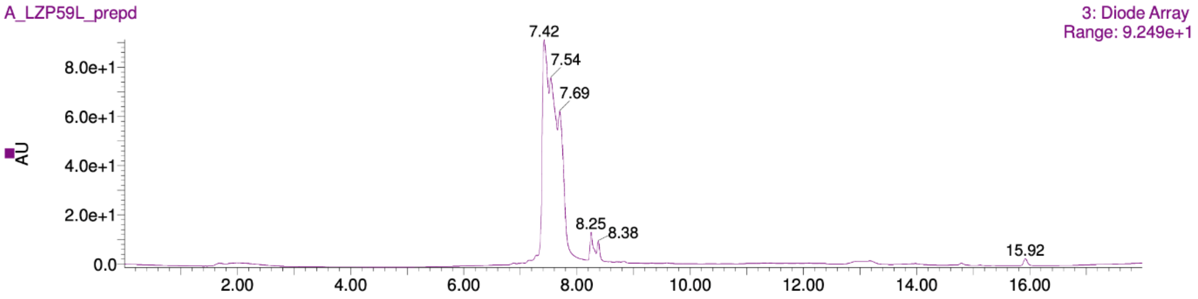

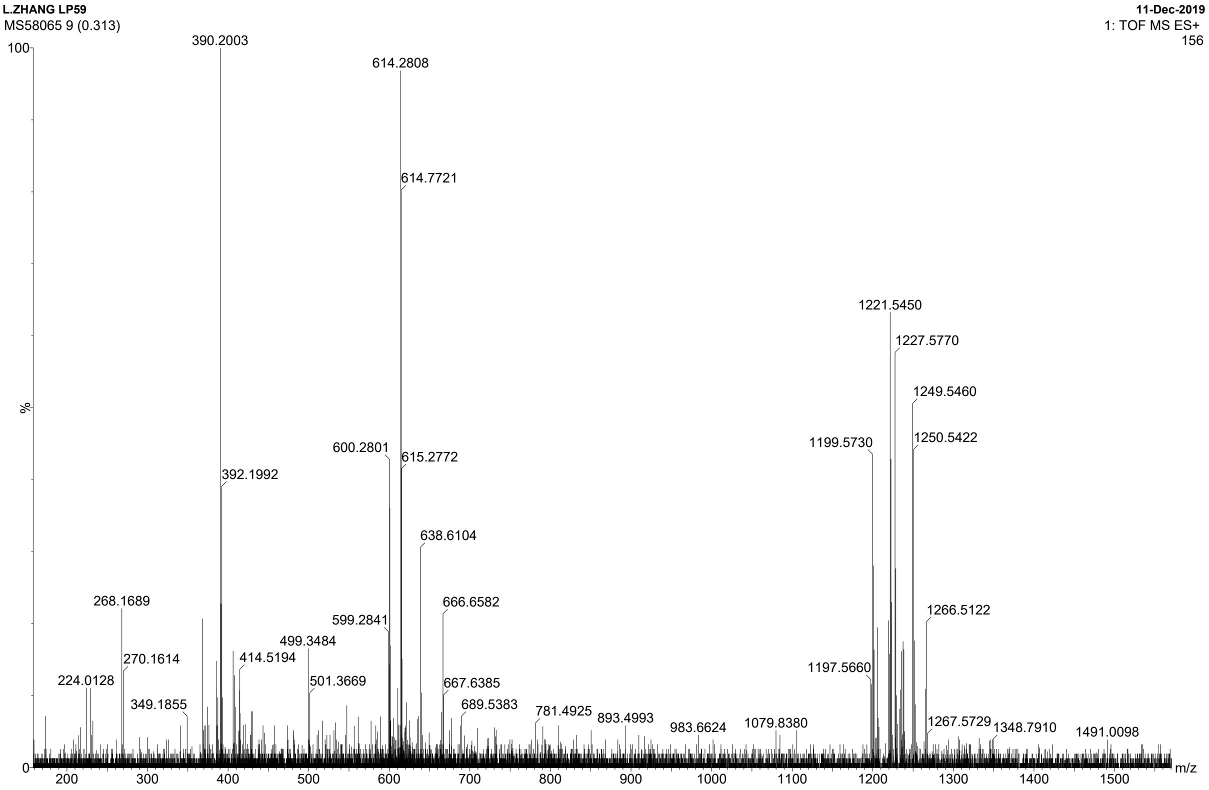

14 Biotin-Ahx-DPhe-Dlys-Dht-Phg(4-guan)-DPP

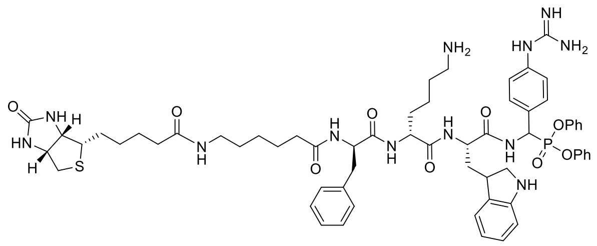

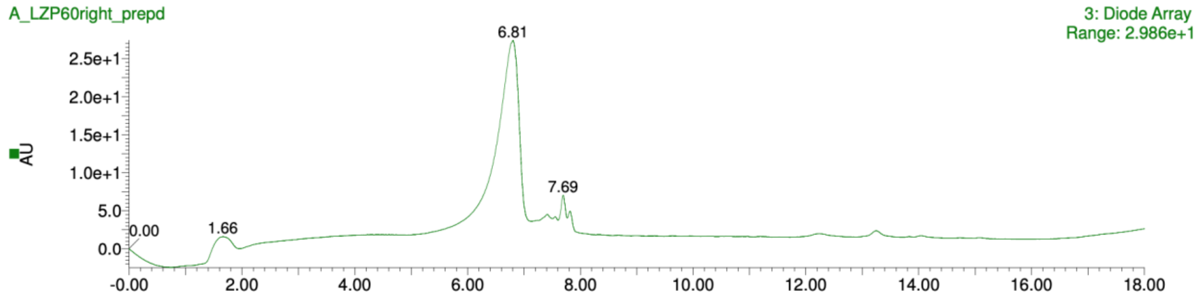

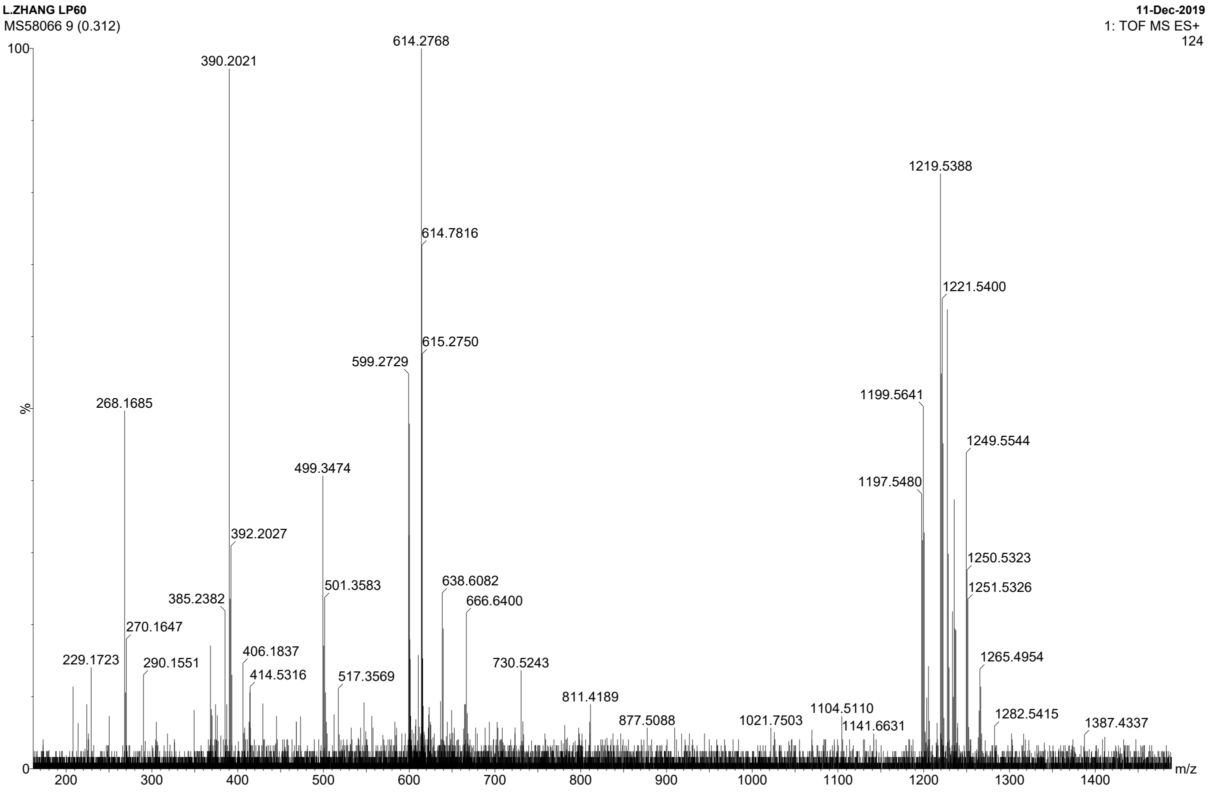

15 Biotin-Ahx-DPhe-Cha-Ser(Ac)-Phg(4-guan)-DPP

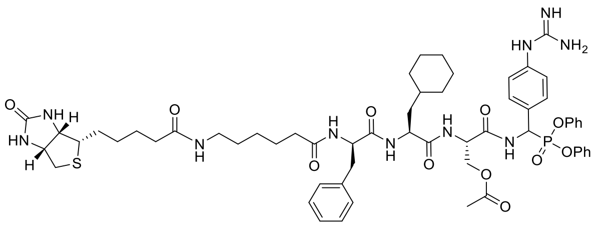

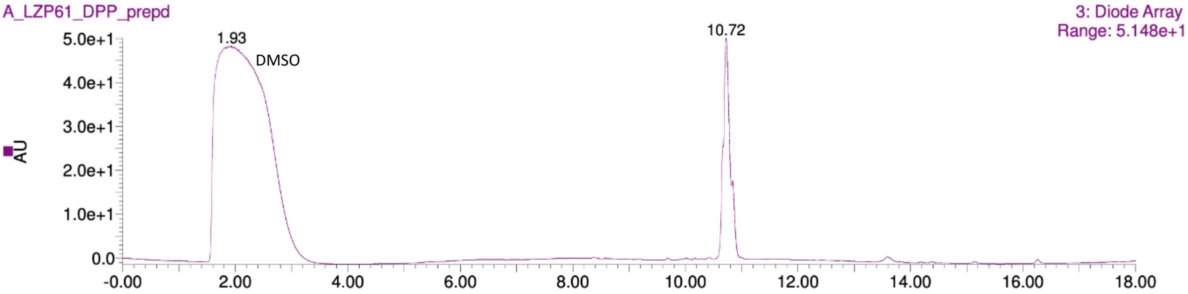

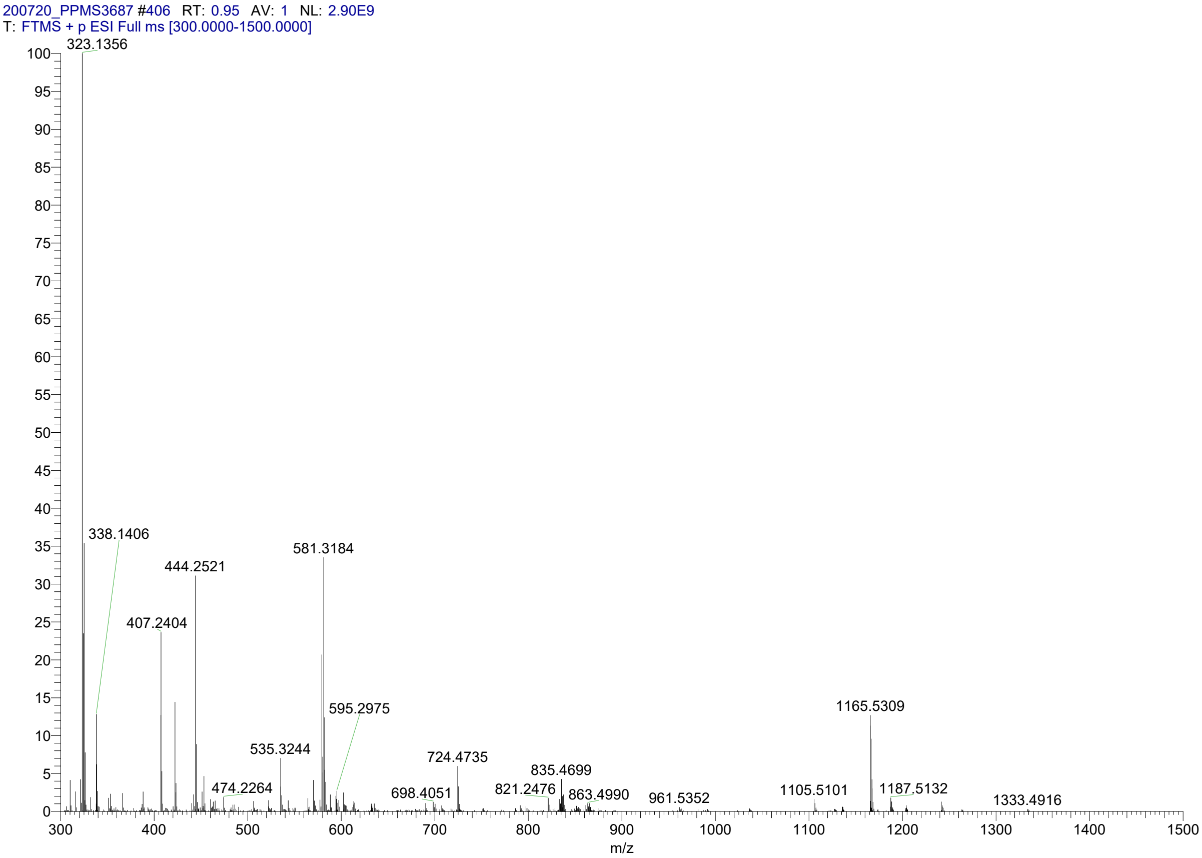

16 Biotin-Ahx-DPhe-Cha-Dht-Arg-DPP

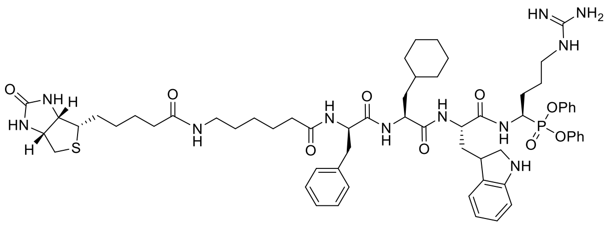

(Double peak may represent separation of the P2 diastereomers)

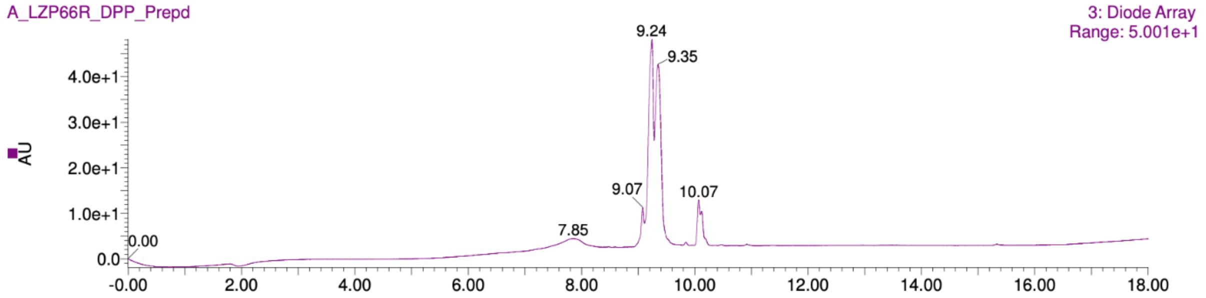

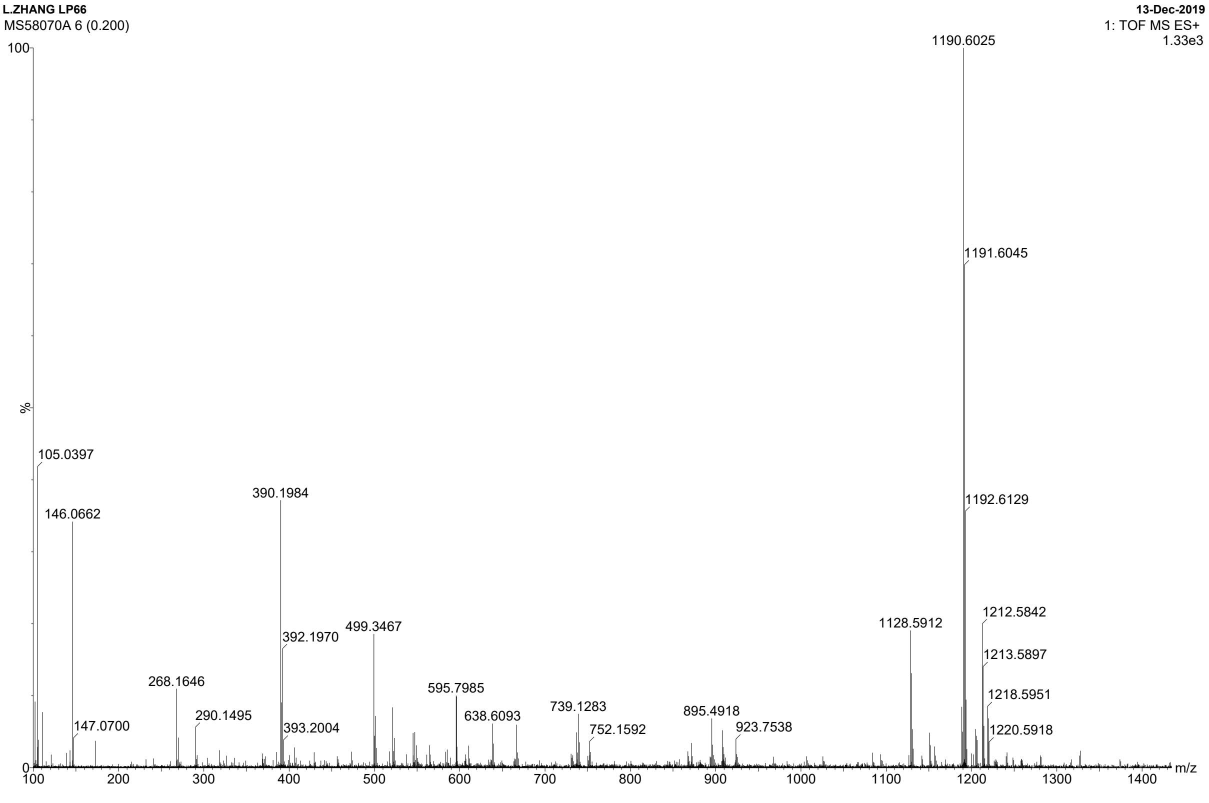

17 Biotin-Ahx-DPhe-Ser(Z)-Dht-Arg-DPP

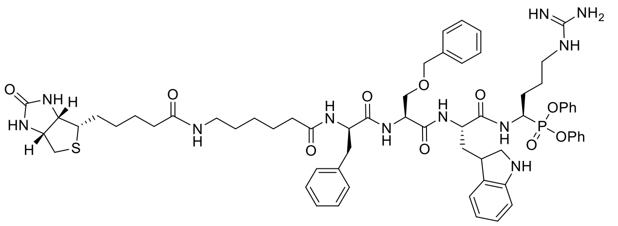

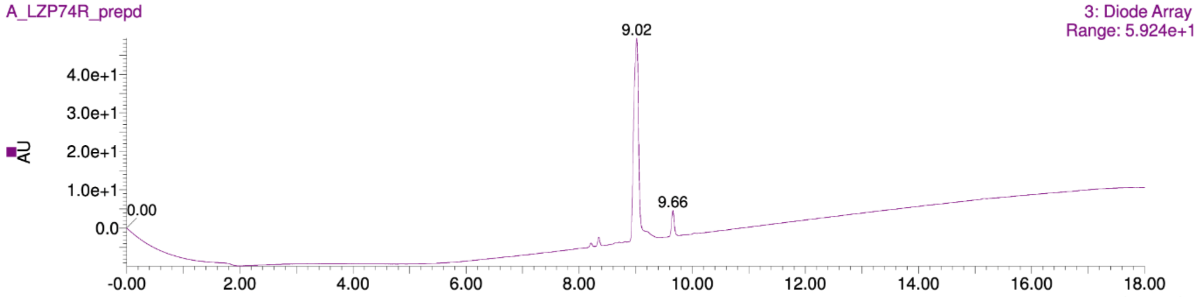

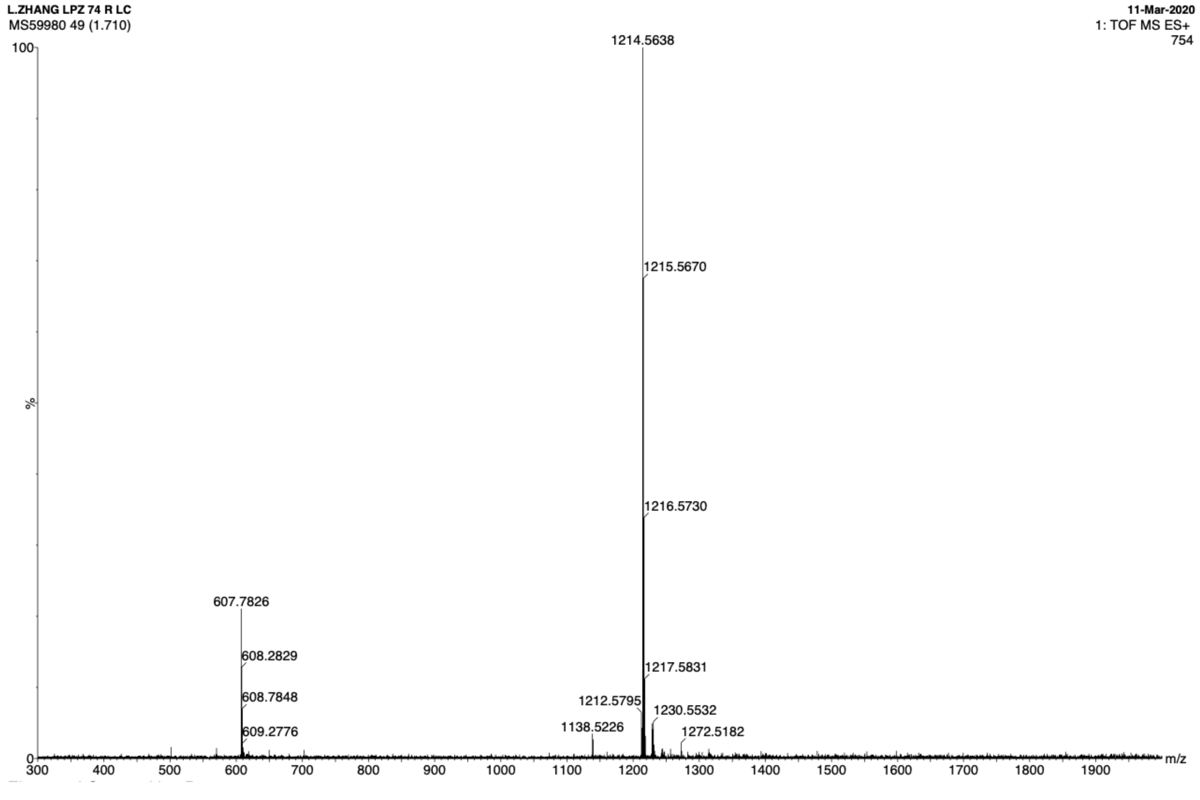

18 Biotin-Ahx-DPhe-Ser(Z)-Dht-DArg-DPP

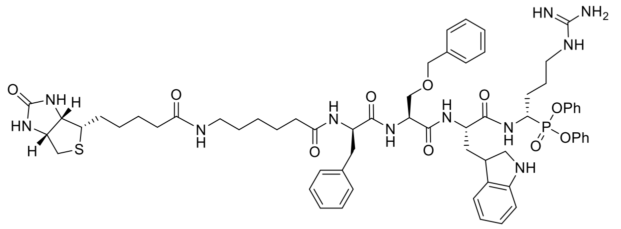

(Double peak may represent separation of the P2 diastereomers)

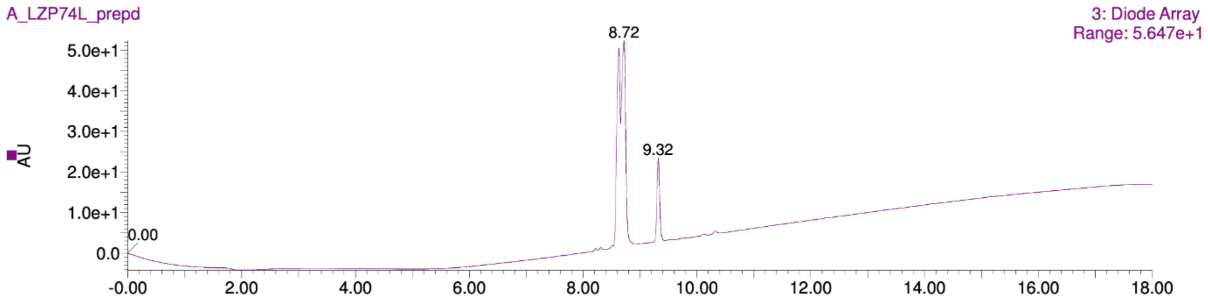

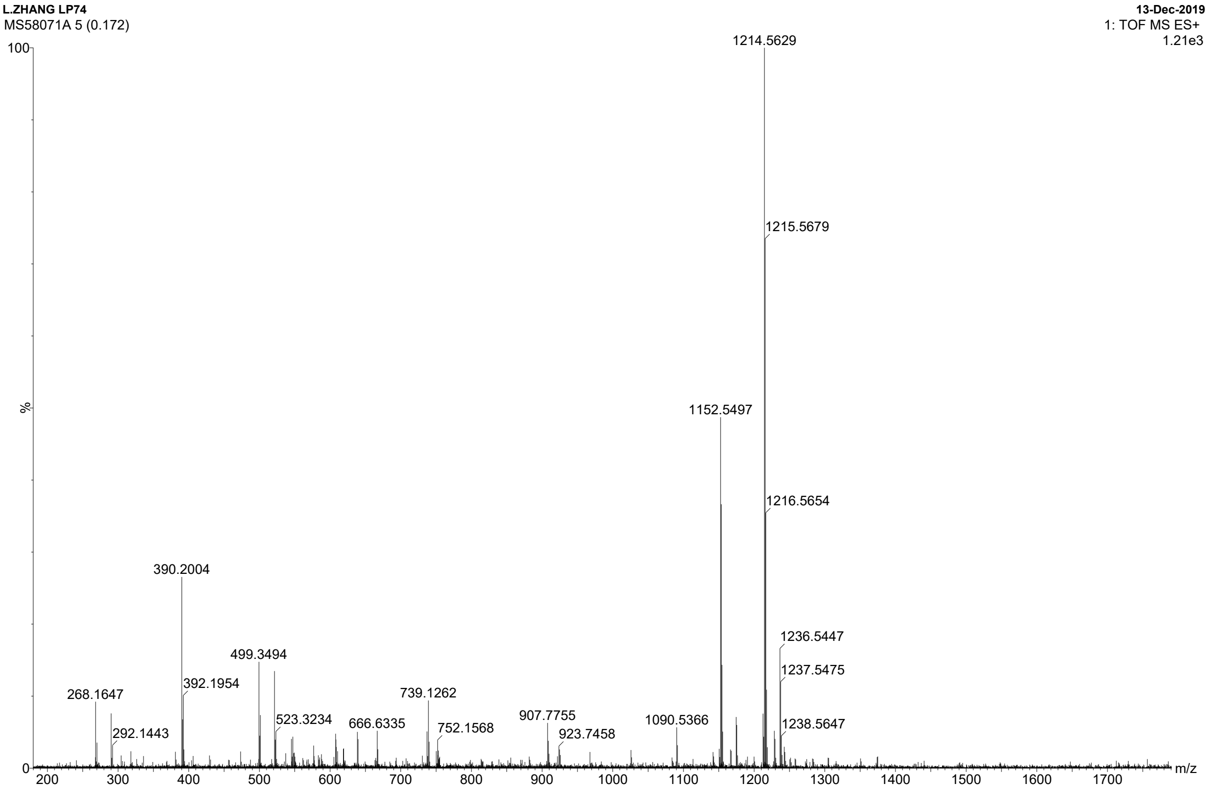

19 Yn-DPhe-Ser(Z)-Dht-Arg-DPP

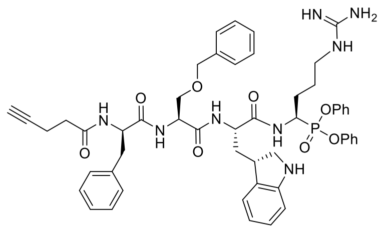

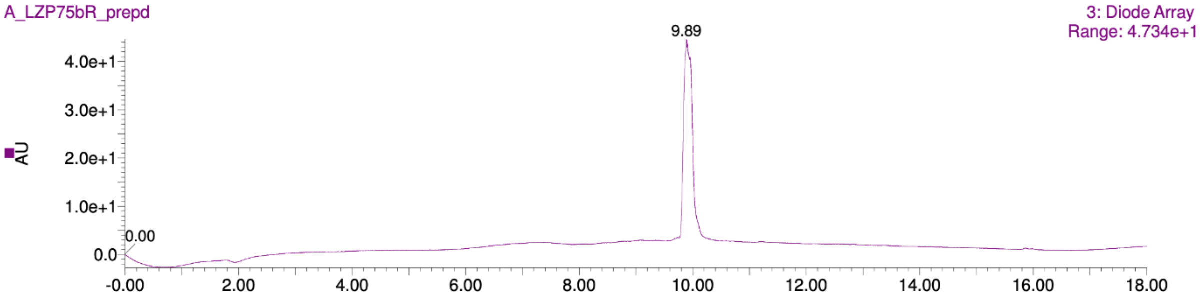

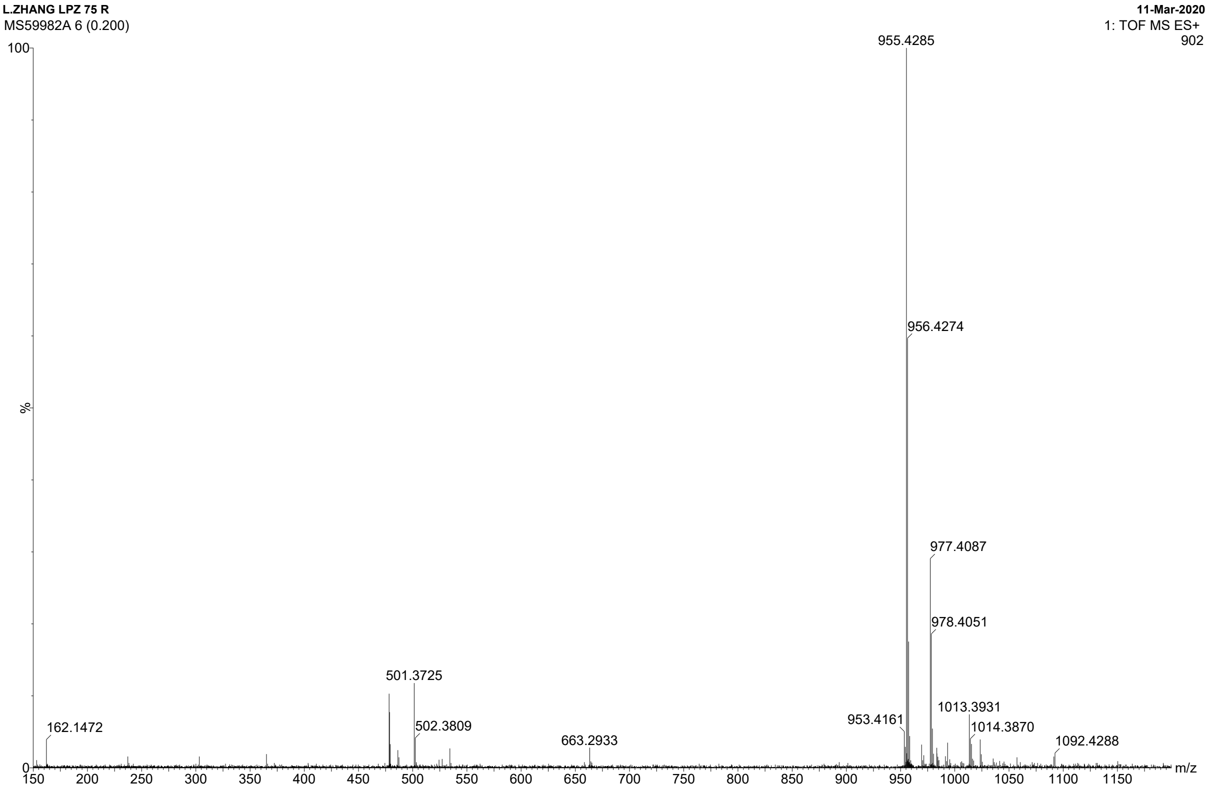

20 Yn-DPhe-Ser(Z)-Dht-DArg-DPP

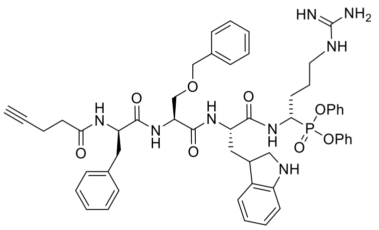

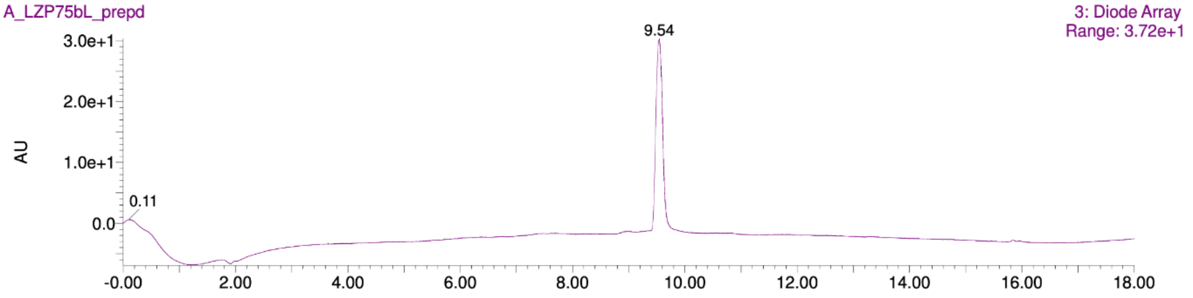

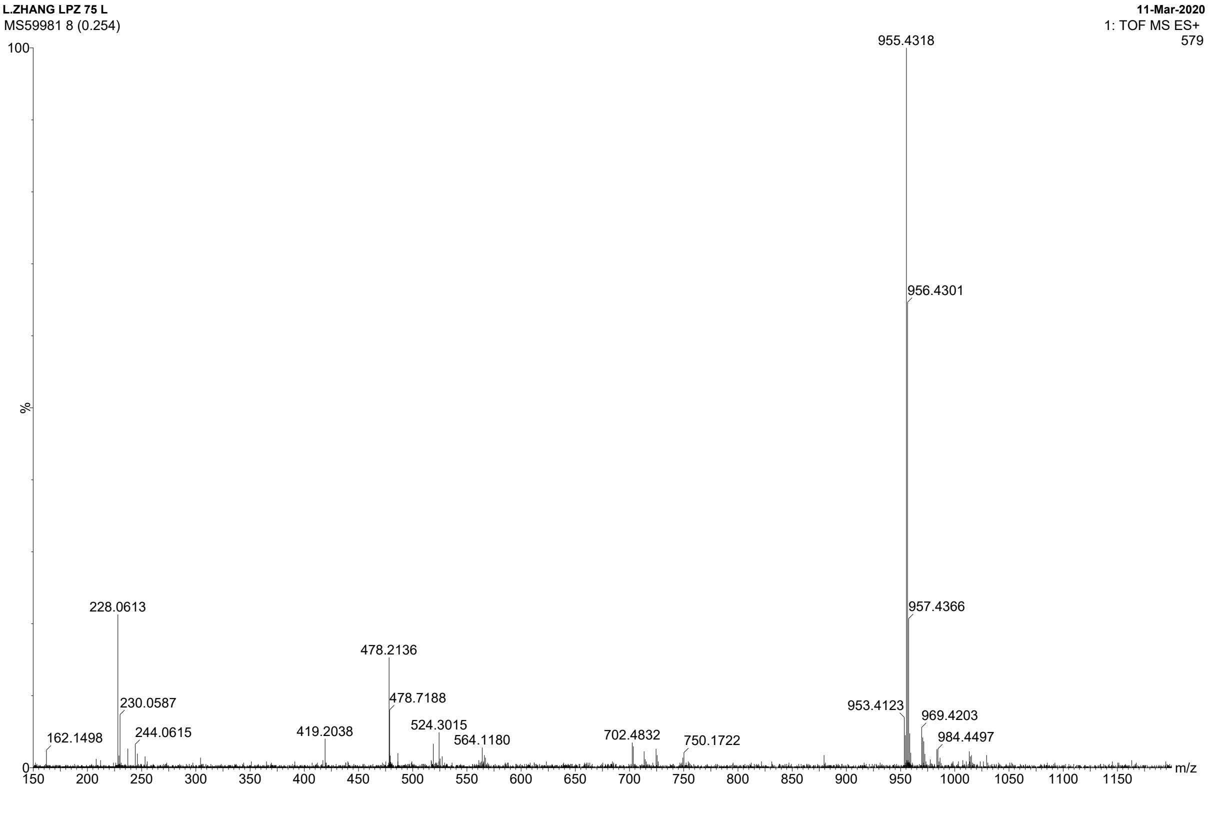

21 Cy5-DPhe-Ser(Z)-Dht-Arg-DPP

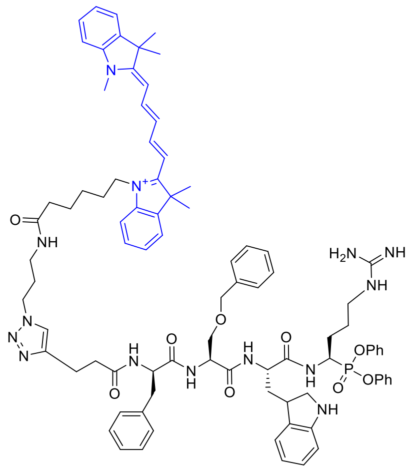

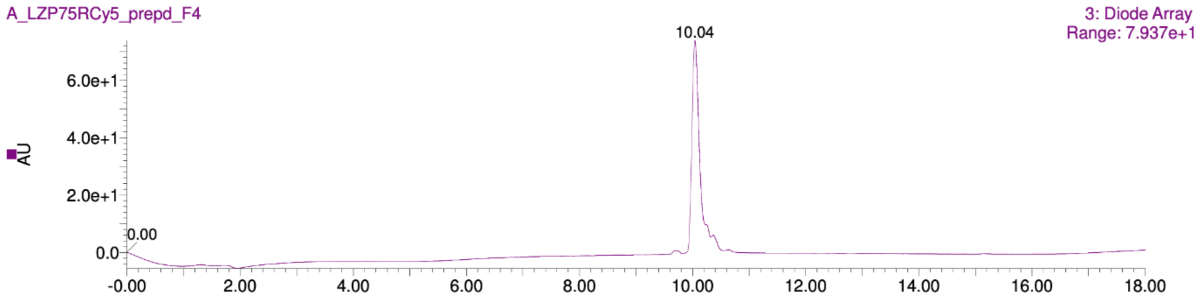

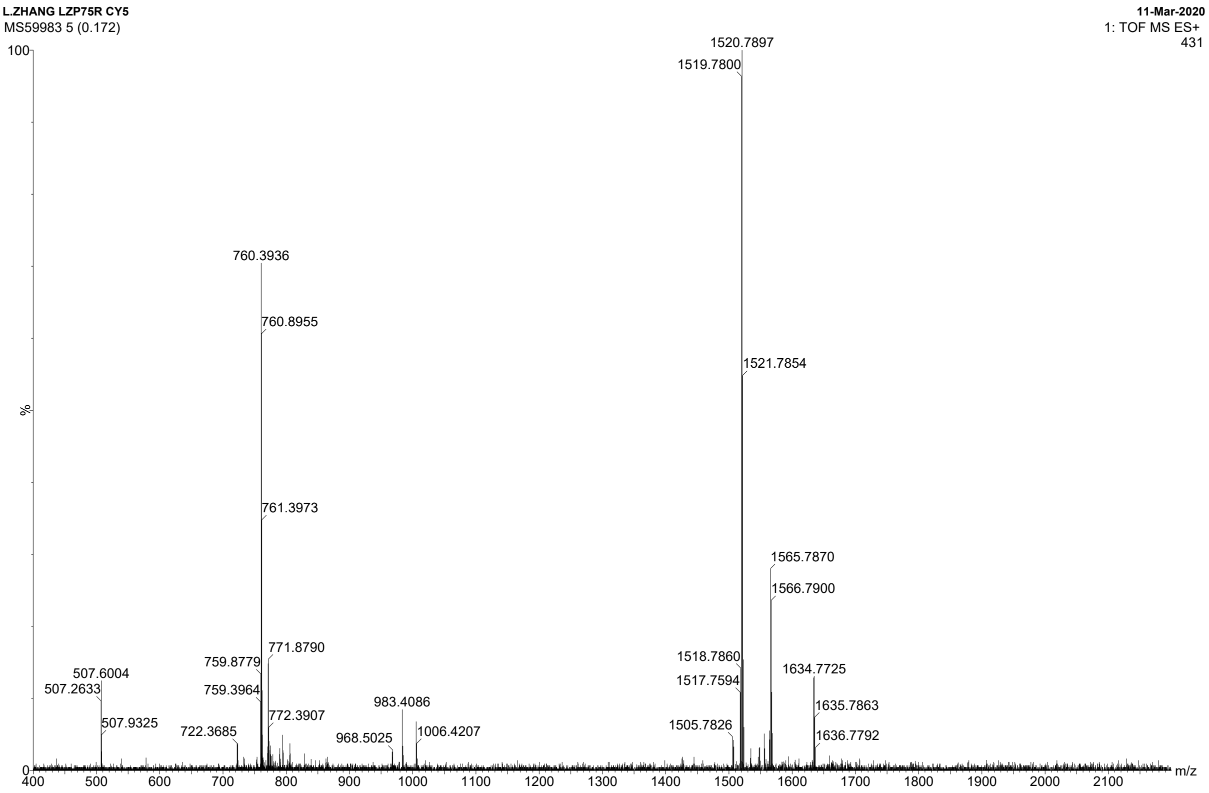

Warheads

25

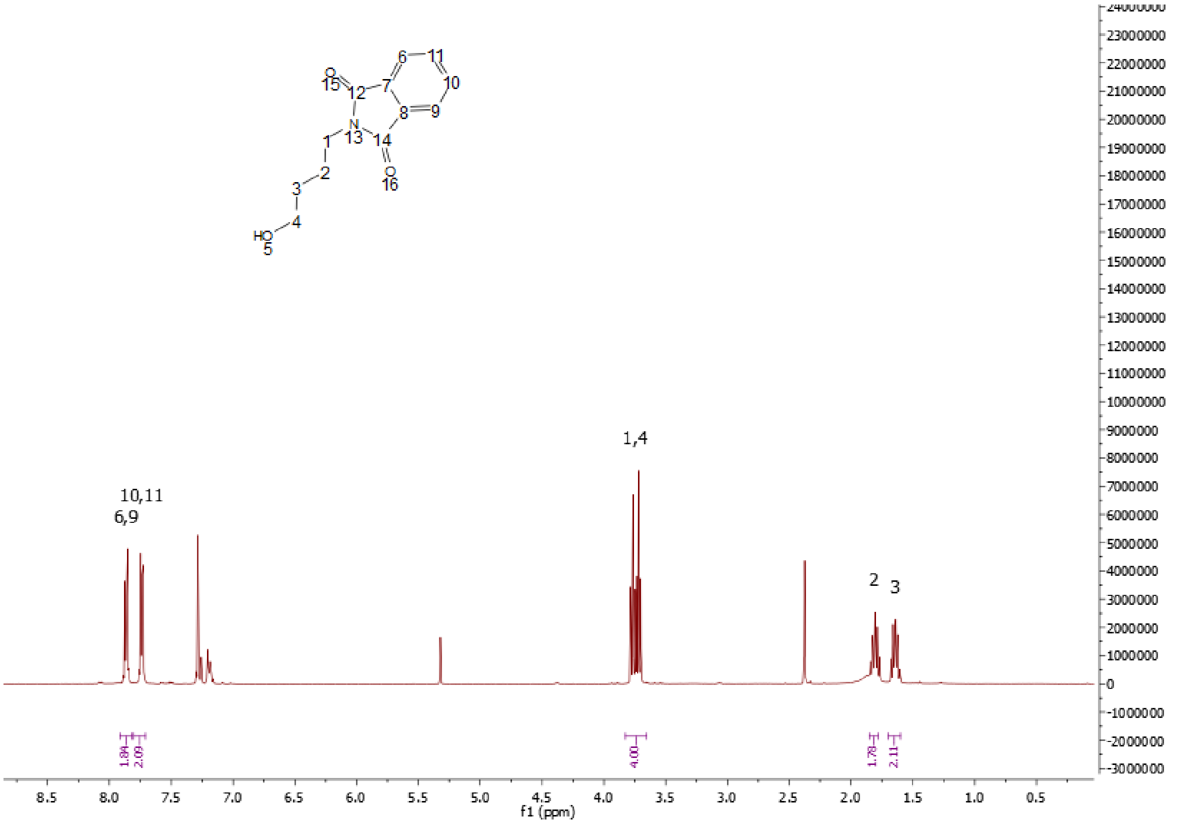

26

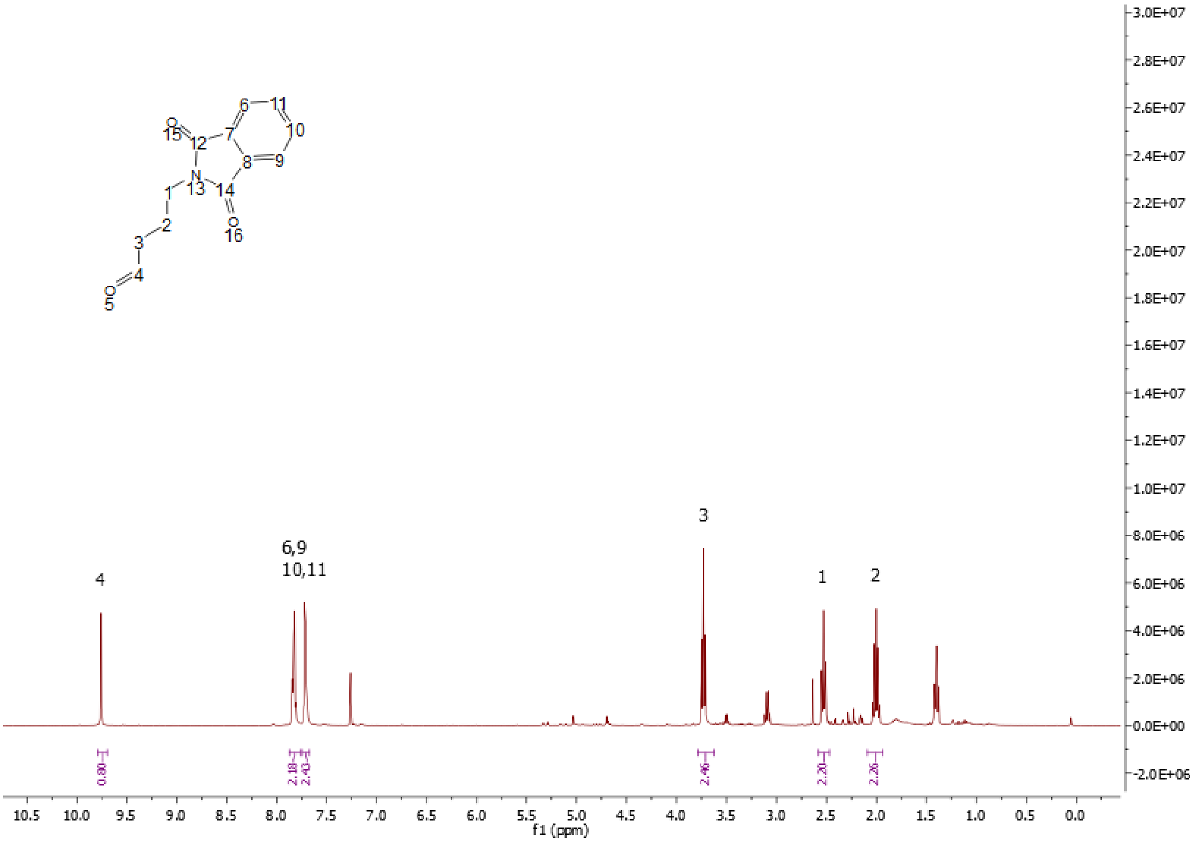

27

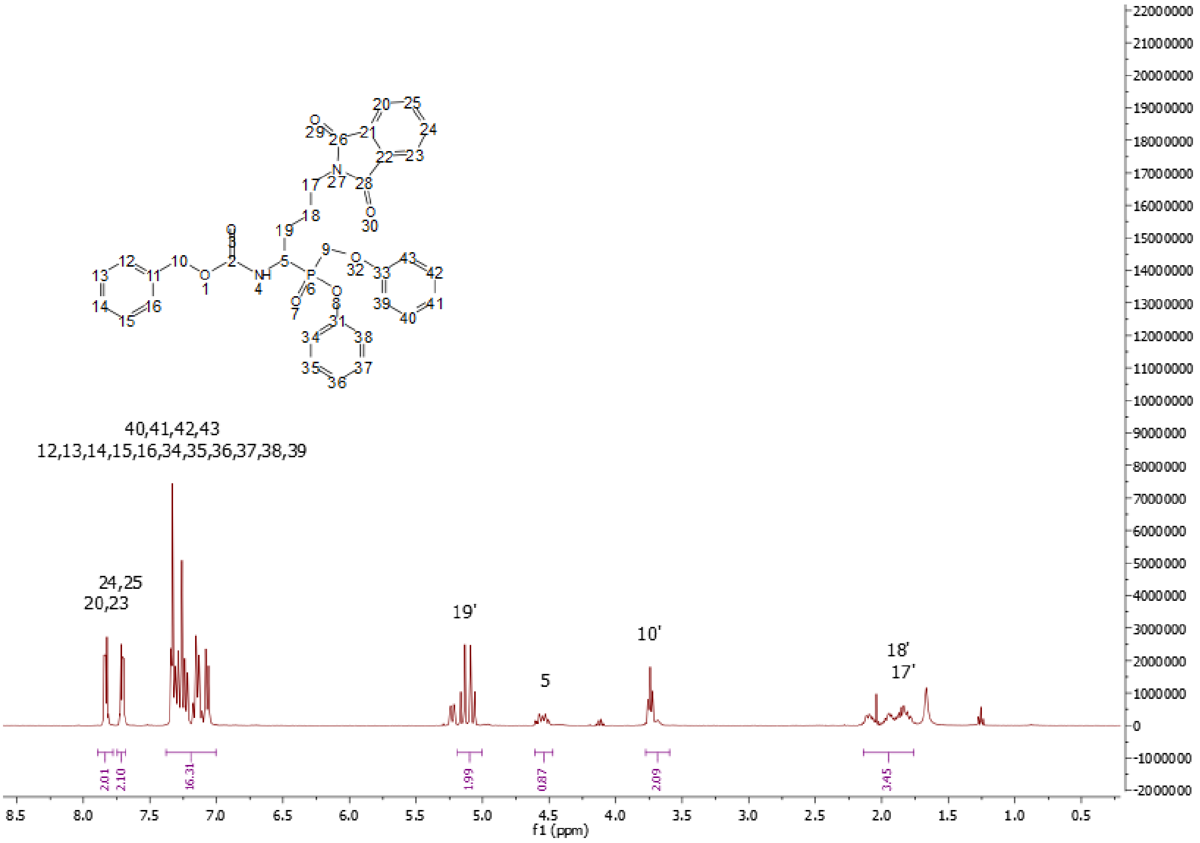
40,41,42,43
12,13,14,15,16,34,35,36,37,38,39
24,25
20,23
19'
5
10'
18'
17'
2.01
2.10
16.31
1.99
0.87
2.09
3.45
f1 (ppm)

28

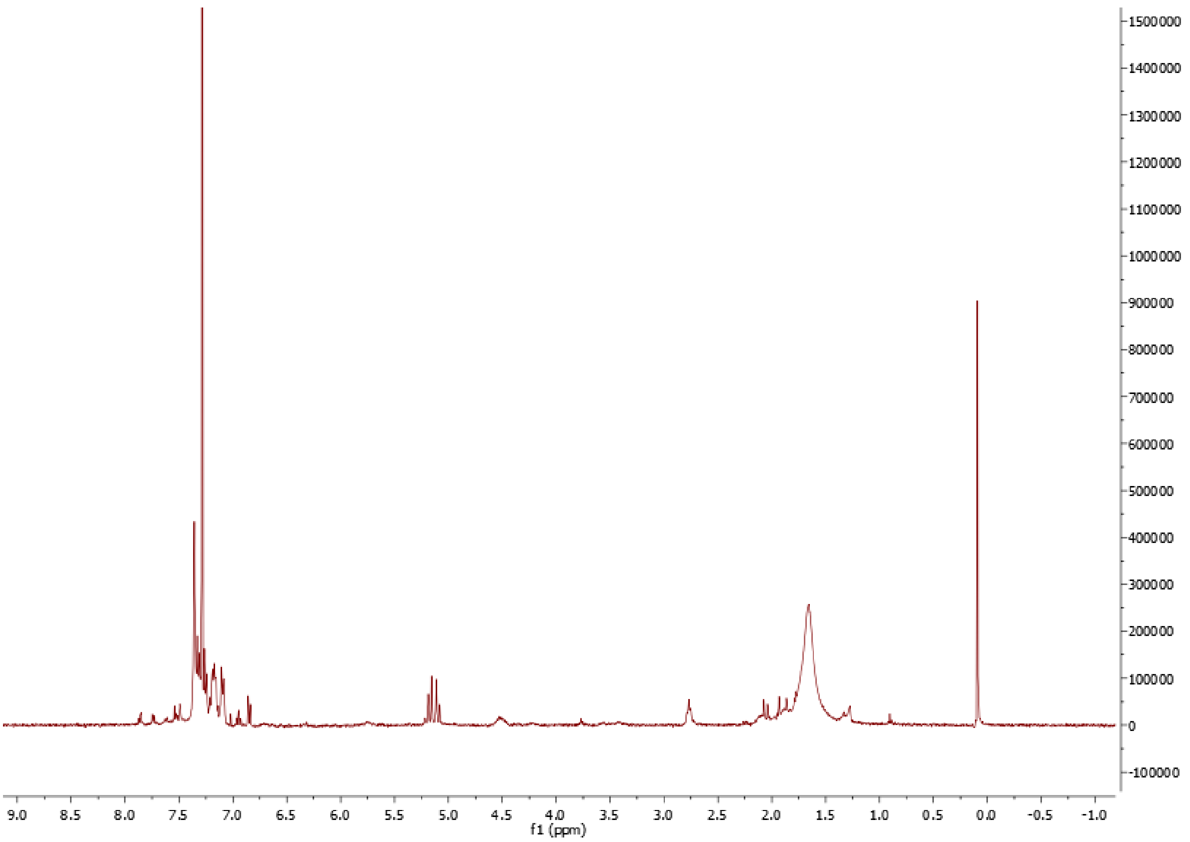
f1 (ppm)

29

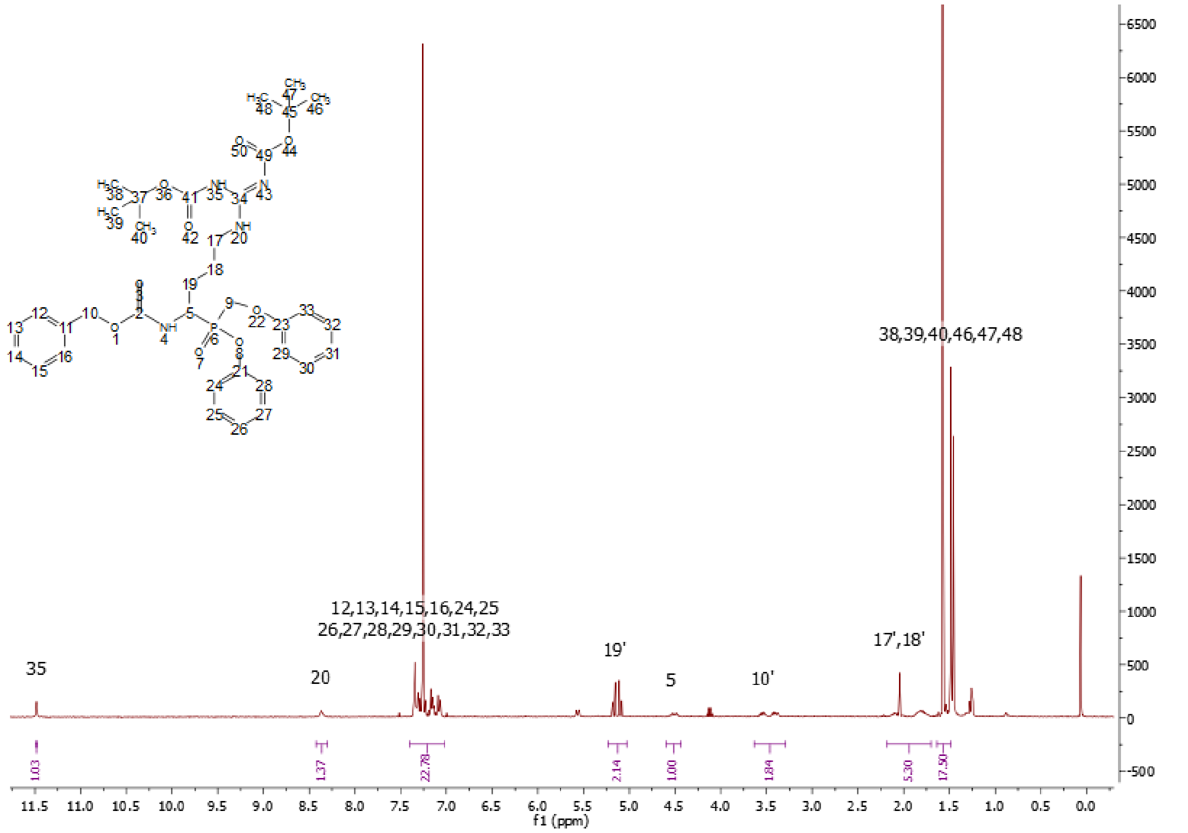

35
20
12,13,14,15,16,24,25
26,27,28,29,30,31,32,33
19'
5
10'
17',18'
38,39,40,46,47,48
1.03
1.37
22.78
2.14
1.00
1.84
5.30
17.50
f1 (ppm)

30

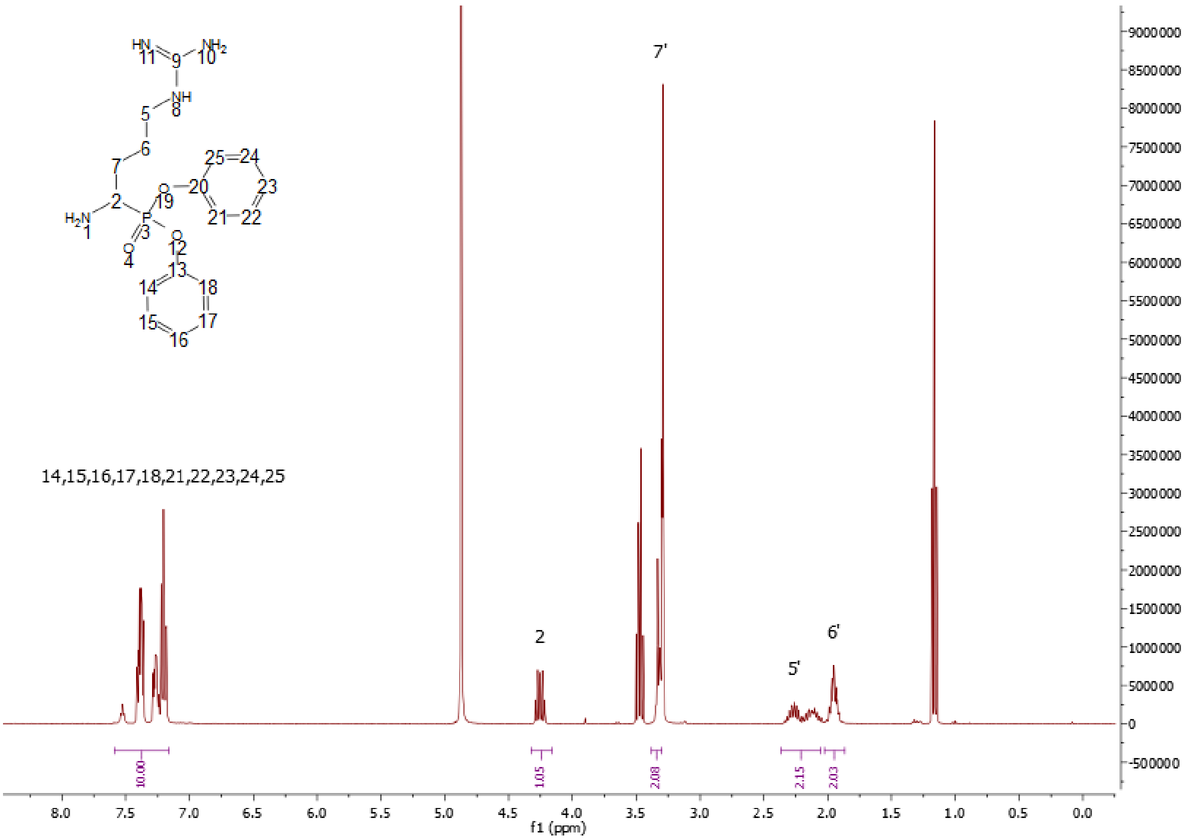

14,15,16,17,18,21,22,23,24,25
2
7'
5'
6'
10.00
1.05
2.08
2.15
2.03
f1 (ppm)

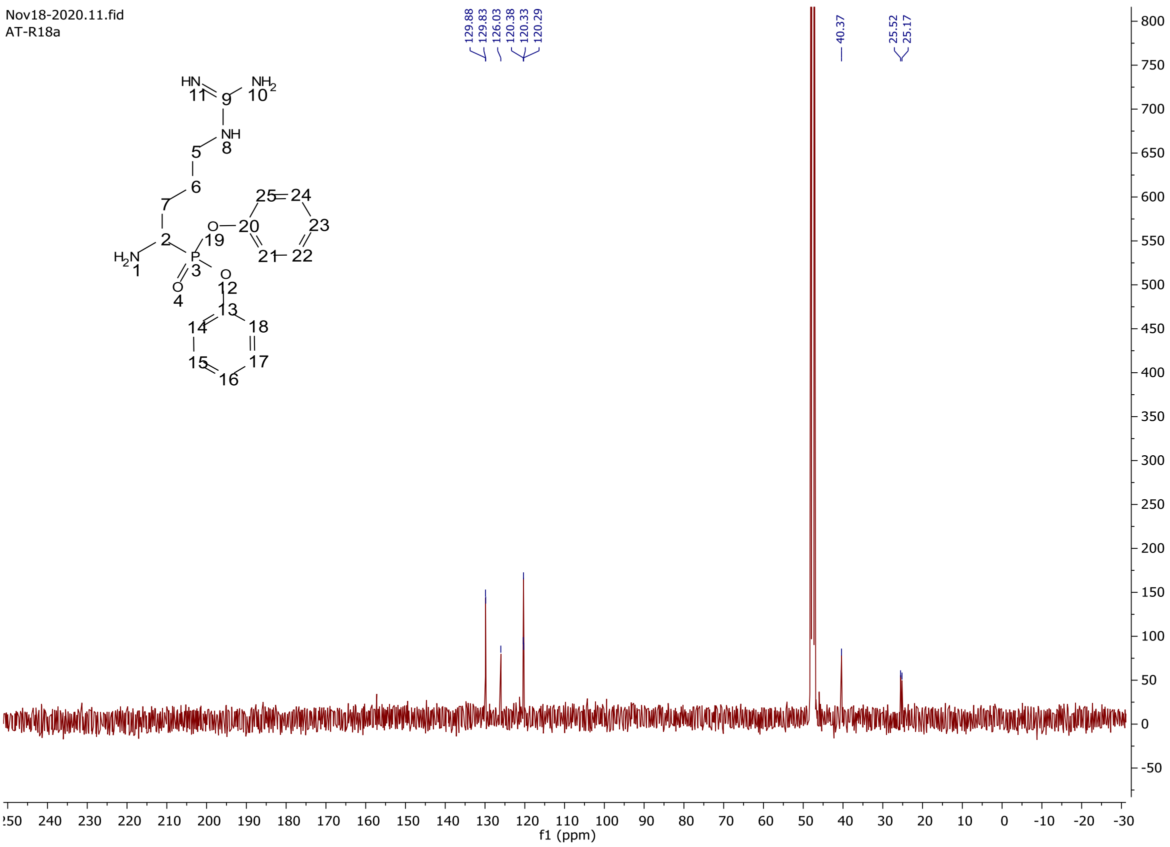
Nov18-2020.11.fid
AT-R18a
129.88
129.83
126.03
120.38
120.33
120.29
40.37
25.52
25.17
f1 (ppm)

31

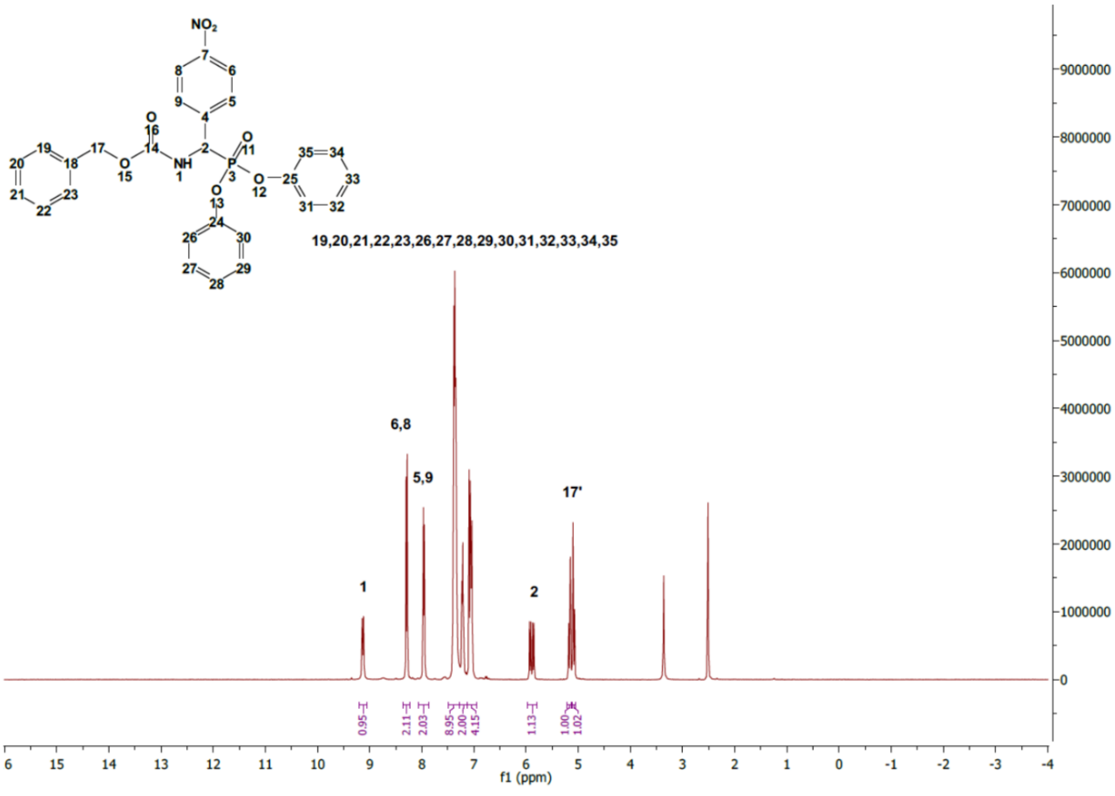
19,20,21,22,23,26,27,28,29,30,31,32,33,34,35
6,8
5,9
17'
1
2
0.95
2.11
2.03
8.95
2.00
4.15
1.13
1.00
1.02
f1 (ppm)

33

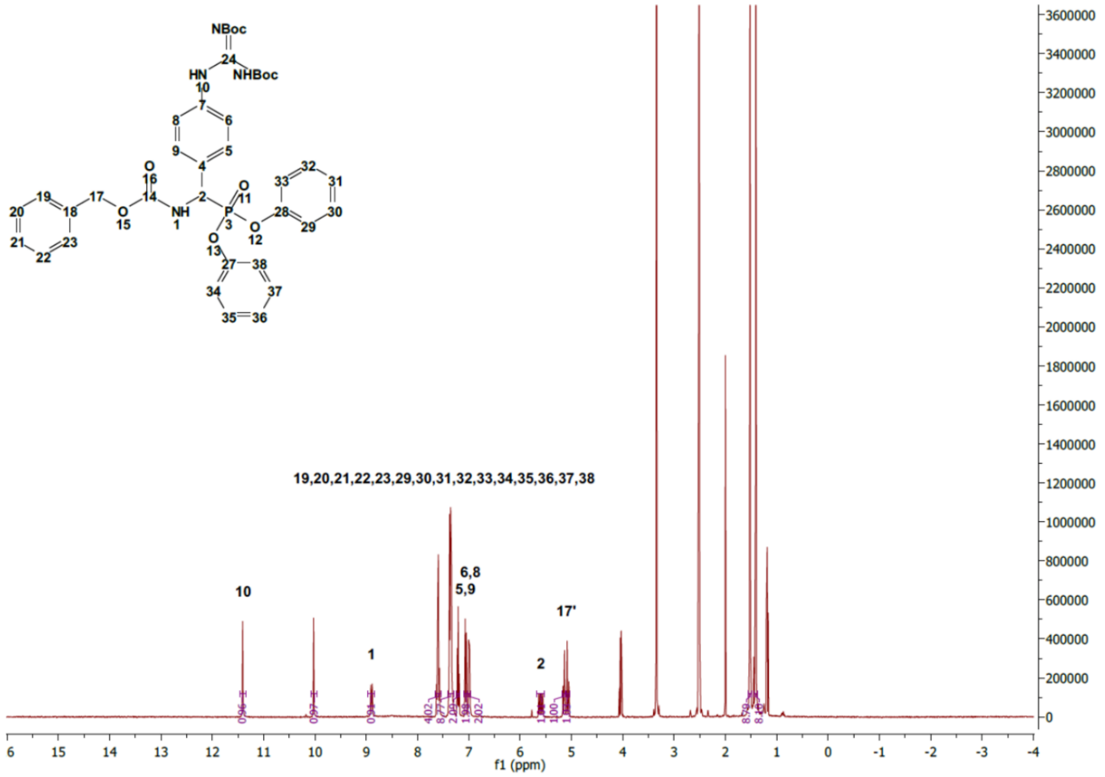
NBoc
NHBoc
HN
19,20,21,22,23,29,30,31,32,33,34,35,36,37,38
10
1
6,8
5,9
2
17'
f1 (ppm)

34

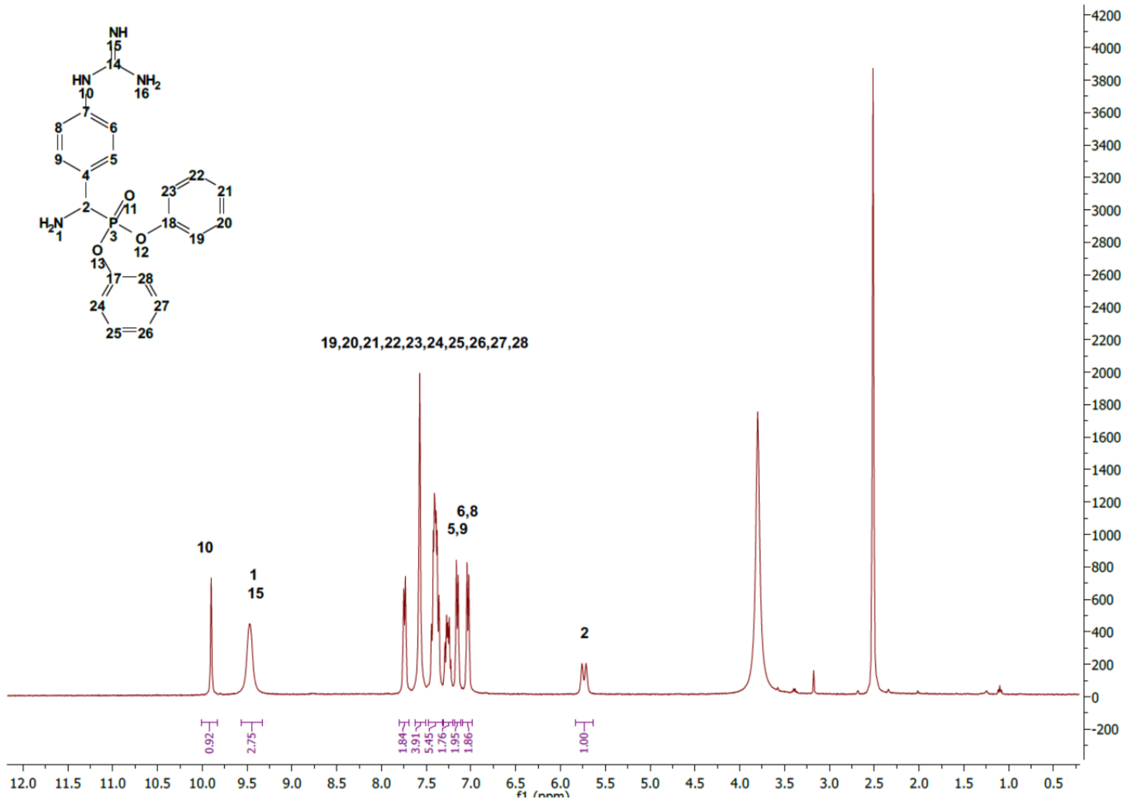
NH
NH2
HN
H2N
19,20,21,22,23,24,25,26,27,28
10
1
15
6,8
5,9
2
f1 (ppm)

4. Original gels and blots

Figure 3A

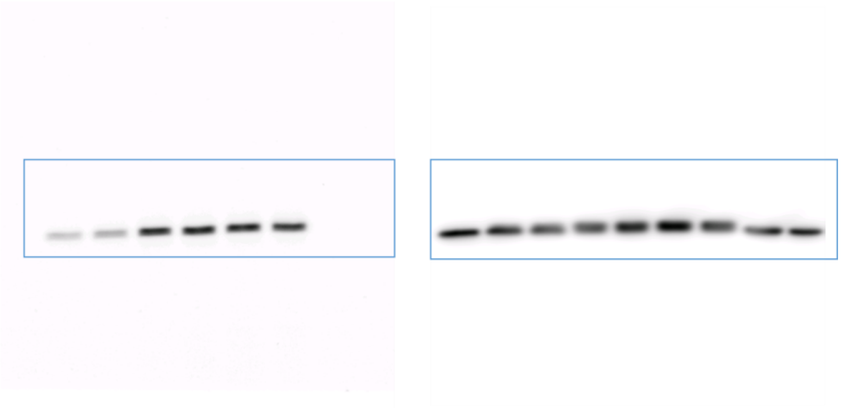

Figure 3B

Before pulldown Pulldown

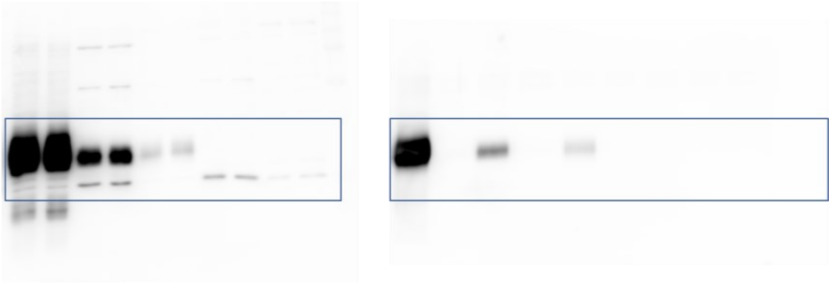

Figure 3C

Before pulldown Pulldown

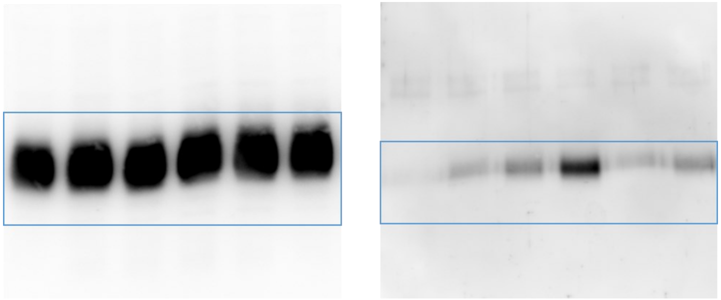

Figure 3D

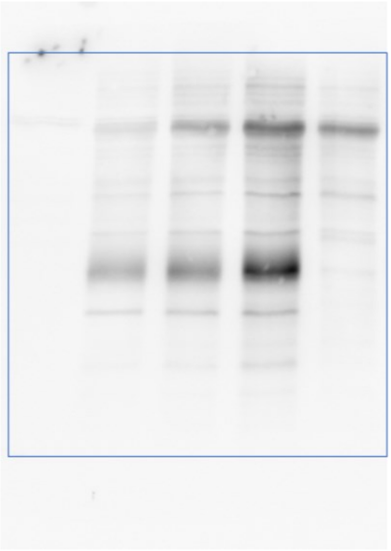

Figure 3E

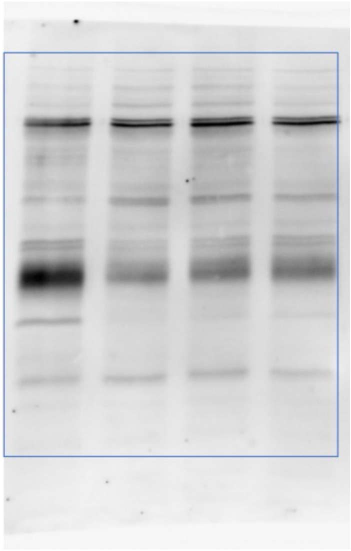

Figure S6

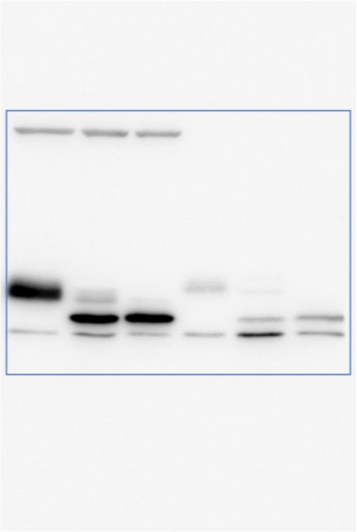

Figure S7

Before Pulldown

Pulldown

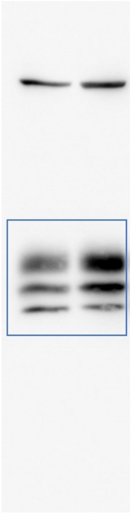

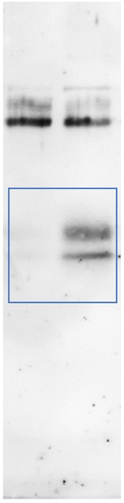

Figure S14

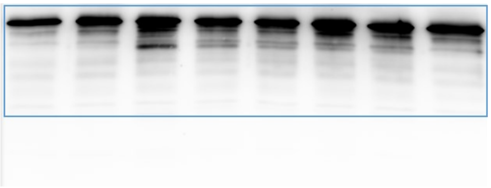

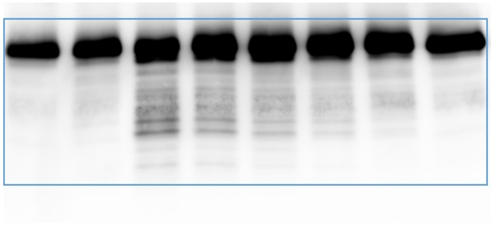

5. References

(1) Silva, L. M. et al. Integration of Two In-Depth Quantitative Proteomics Approaches Determines the Kallikrein-Related Peptidase 7 (KLK7) Degradome in Ovarian Cancer Cell Secretome. *Mol. Cell. Proteomics* **2019**, *18* (5), 818–836.

(2) Kryza, T. et al. The Molecular Function of Kallikrein-related Peptidase 14 Demonstrates a Key Modulatory Role in Advanced Prostate Cancer. *Mol. Oncol.* **2020**, *14* (1), 105–128.

(3) Poreba, M.; Salvesen, G. S.; Drag, M. Synthesis of a HyCoSuL Peptide Substrate Library to Dissect Protease Substrate Specificity. *Nat. Protoc.* **2017**, *12* (10), 2189–2214.

(4) Joossens, J.; Van der Veken, P.; Lambeir, A.-M.; Augustyns, K.; Haemers, A. Development of Irreversible Diphenyl Phosphonate Inhibitors for Urokinase Plasminogen Activator. *J. Med. Chem.* **2004**, *47* (10), 2411–2413.

(5) Weng, S. S. H. et al. Sensitive Determination of Proteolytic Proteoforms in Limited Microscale Proteome Samples. *Mol. Cell. Proteomics* **2019**, *18* (11), 2335–2347.

(6) Liang, G. et al. Human Kallikrein 6 Inhibitors with a Para-Amidobenzylanmine P1 Group Identified through Virtual Screening. *Bioorg. Med. Chem. Lett.* **2012**, *22* (7), 2450–2455.

(7) Kabsch, W. XDS. *Acta Crystallogr. Sect. D Biol. Crystallogr.* **2010**, *66* (2), 125–132.

(8) McCoy, A. J. et al. Phaser Crystallographic Software. *J. Appl. Crystallogr.* **2007**, *40* (4), 658–674.

(9) Emsley, P.; Cowtan, K. Coot : Model-Building Tools for Molecular Graphics. *Acta Crystallogr. Sect. D Biol. Crystallogr.* **2004**, *60* (12), 2126–2132.

(10) Adams, P. D. et al. PHENIX : A Comprehensive Python-Based System for Macromolecular Structure Solution. *Acta Crystallogr. Sect. D Biol. Crystallogr.* **2010**, *66* (2), 213–221.

(11) Partridge, J.; Flaherty, P. An In Vitro FluoroBlok Tumor Invasion Assay. *J. Vis. Exp.* **2009**, *20* (29), 1475.
